# Maternal nutrition intervention and maternal complications in 4 districts of Bangladesh: A nested cross-sectional study

**DOI:** 10.1371/journal.pmed.1002927

**Published:** 2019-10-04

**Authors:** Catherine S. Todd, Zakaria Chowdhury, Zeba Mahmud, Nazia Islam, Sadia Shabnam, Musarrat Parvin, Alissa Bernholc, Andres Martinez, Bachera Aktar, Kaosar Afsana, Tina Sanghvi

**Affiliations:** 1 Global Health, Population, and Nutrition, FHI 360, Durham, North Carolina, United States of America; 2 Alive & Thrive Program Office, Dhaka, Bangladesh; 3 BRAC, Dhaka, Bangladesh; 4 BRAC James P. Grant School of Public Health, BRAC University, Dhaka, Bangladesh; 5 Alive & Thrive Headquarters Office, Washington, District of Columbia, United States of America; Cornell University, UNITED STATES

## Abstract

**Background:**

Maternal morbidity is common in Bangladesh, where the maternal mortality rate has plateaued over the last 6 years. Maternal undernutrition and micronutrient deficiencies contribute to morbidity, but few interventions have measured maternal outcomes. We compared reported prevalence of antepartum, intrapartum, and postpartum complications among recently delivered women between maternal nutrition intervention and control areas in Bangladesh.

**Methods and findings:**

We conducted a cross-sectional assessment nested within a population-based cluster-randomized trial comparing a nutrition counseling and micronutrient supplement intervention integrated within a structured home-based maternal, newborn, and child health (MNCH) program to the MNCH program alone in 10 sub-districts each across 4 Bangladesh districts. Eligible consenting women, delivering within 42–60 days of enrollment and identified by community-level health workers, completed an interviewer-administered questionnaire detailing the index pregnancy and delivery and allowed review of their home-based care register. We compared pooled and specific reported antepartum, intrapartum, and postpartum complications between study groups using hierarchical logistic regression. There were 594 women in the intervention group and 506 in the control group; overall, mean age was 24 years, 31% were primiparas, and 39% reported facility-based delivery, with no significant difference by study group. There were no significant differences between the intervention and control groups in household-level characteristics, including reported mean monthly income (intervention, 6,552 taka, versus control, 6,017 taka; *p* = 0.48), having electricity (69.6% versus 71.4%, *p* = 0.84), and television ownership (41.1% versus 38.7%, *p* = 0.81). Women in the intervention group had higher recorded iron and folic acid and calcium supplement consumption and mean dietary diversity scores, but reported anemia rates were similar between the 2 groups (5.7%, intervention; 6.5%, control; *p* = 0.83). Reported antepartum (69.4%, intervention; 79.2%, control; *p* = 0.12) and intrapartum (41.4%, intervention; 48.5%, control; *p* = 0.18) complication rates were high and not significantly different between groups. Reported postpartum complications were significantly lower among women in the intervention group than the control group (33.5% versus 48.2%, *p* = 0.02), and this difference persisted in adjusted analysis (adjusted odds ratio [AOR] = 0.51, 95% CI 0.32–0.82; *p <* 0.001). For specific conditions, odds of retained placenta (AOR = 0.35, 95% CI 0.19–0.67; *p* = 0.001), postpartum bleeding (AOR = 0.37, 95% CI 0.15–0.92; *p* = 0.033), and postpartum fever/infection (AOR = 0.27, 95% CI 0.11–0.65; *p* = 0.001) were significantly lower in the intervention group in adjusted analysis. There were no significant differences in reported hospitalization for antepartum (49.8% versus 45.1%, *p* = 0.37), intrapartum (69.9% versus 59.8%, *p* = 0.18), or postpartum (36.1% versus 29.9%, *p* = 0.49) complications between the intervention and control groups. The main limitations of this study are outcome measures based on participant report, non-probabilistic selection of community-level workers’ catchment areas for sampling, some missing data for variables derived from secondary sources (e.g., dietary diversity score), and possible recall bias for reported dietary intake and supplement use.

**Conclusions:**

Reported overall postpartum and specific intrapartum and postpartum complications were significantly lower for women in intervention areas than control areas, despite similar rates of facility-based delivery and hospitalization for reported complications, in this exploratory analysis. Maternal nutrition interventions providing intensive counseling and micronutrient supplements may reduce some pregnancy complications or impact women’s ability to accurately recognize complications, but more rigorous evaluation is needed for these outcomes.

## Abstract 2

Catherine Todd and colleagues reveal that nutritional education and micronutrient supplements can reduce intrapartum and postpartum complications in pregnant women in Bangladesh.

## Author summary

**Why was this study done?:**

- Adequate nutrition during pregnancy reduces risks of poor infant outcomes, like low birthweight, but there is relatively little information about whether nutrition is linked to reducing a woman’s risk of pregnancy complications.
- Maternal nutrition interventions starting during pregnancy are being implemented at scale in multiple settings, including in Bangladesh, and determining whether these interventions reduce maternal complications may strengthen efforts to implement similar interventions to optimize maternal and newborn health.

**What did the researchers do and find?:**

- At the time of the endline evaluation for the parent trial—which compared an intervention of home-based intensive maternal nutrition counseling and iron and calcium supplement provision combined with home-based antenatal care to home-based antenatal care alone—we interviewed women who had delivered within the last 60 days to inquire about complications during pregnancy, during delivery, and after delivery.
- This study included 594 women in the intervention group and 506 in the control group across 4 districts in Bangladesh; women in the 2 groups were similar with regard to number of prior pregnancies, age, and household assets.
- Reported complication rates during pregnancy or during delivery were somewhat lower among women from intervention areas, while reported complications after delivery were significantly lower among women from intervention areas, compared to those from control areas.

**What do these findings mean?:**

- Differences in reported complication rates may be due to actual differing levels resulting from intervention exposure, or to different perceptions of what conditions constitute pregnancy complications, which could also result from intervention exposure.
- The intervention did not appear to impact care-seeking during pregnancy or during labor based on similar skilled/facility-based care statistics for the intervention and control groups.
- Community-based care providers recorded key obstetric events accurately and at high rates, but greater understanding of client and provider perceptions regarding what constitutes maternal complications and ensuring provision of appropriate care are needed.
- Given the cross-sectional nature of the study and that complications were self-reported, the findings should be interpreted with caution.

## Introduction

While maternal mortality has decreased globally over the last 2 decades, the maternal mortality ratio (MMR) and associated maternal morbidity remain high and disproportionately occur in developing contexts, including those where malnutrition and micronutrient deficiencies are common among children and adults [1–3]. The most common direct causes of maternal mortality are hemorrhage (27%), hypertensive disorders of pregnancy including eclampsia/pre-eclampsia (14%), and sepsis (11%); aggregated indirect causes also contribute substantively, comprising approximately 25% of maternal deaths [3]. Malnutrition and micronutrient deficiencies both potentially contribute to indirect causes of maternal mortality and exacerbate direct causes [3,4].

The impact of nutrition on pregnancy can result from sequelae of childhood malnutrition, such as short stature and pelvic contracture leading to obstructed labor and potential uterine rupture [4–6]. Maternal malnutrition and underweight status, measured by mid-upper arm circumference (MUAC) at 19 weeks gestation, was associated with a 25% increased risk of death for every 1-cm decrement from the median (21 cm) in MUAC in Nepal [7]. In Bangladesh, a MUAC less than 21.5 cm was associated with an increased risk of puerperal sepsis and hemorrhage [8]. A larger historical population-based analysis in Germany during and after a grain price increase in the 1700–1800s detected higher maternal mortality rates following the increase, suggestive of increased maternal risk with poor overall nutrition [9]. Specific micronutrient deficiencies are also associated with poor maternal outcomes; night blindness, suggestive of vitamin A deficiency, was predictive of a 3.8 times greater risk of maternal death in Nepal [10].

Nutrition-focused and micronutrient supplementation interventions during pregnancy have been implemented at scale in a variety of settings. However, most of these interventions focused on neonatal and child health indicators, such as low birth weight, as the primary outcome measures, with relatively fewer assessing the impact of improved overall nutrition status and/or caloric intake on reducing risk of maternal complications [4]. Of evaluated maternal nutrition interventions, one found reduced rates of eclampsia and pre-eclampsia [11] and another reported reduced rates of iron-deficiency anemia [12]. Some interventions that included calcium and iron supplementation also reported reductions in postpartum hemorrhage (PPH) [4,13,14]. Vitamin A supplementation trials reduced night blindness and mortality rates in Nepal, a context with high MMR and widespread vitamin A deficiency [10]. In a larger meta-analysis, vitamin A supplementation did not reduce overall maternal mortality but was associated with lower rates of postpartum infection and anemia in smaller studies with low-quality evidence [15]. By contrast, zinc, iodine, and folate supplementation did not have specific positive impacts on maternal outcome measures [16–18].

In Bangladesh, the MMR decreased substantially over a 2-decade period, with this decrease partly attributed to improved care access with expanded levels of antenatal care (ANC) coverage, facility-based birth and skilled birth attendance, and postnatal care (PNC) provision [19,20]. Also contributing to this rapid improvement are programmatic approaches to improve maternal, newborn, and child health (MNCH) care, particularly in rural areas, implemented at large scale [21,22]. Bangladesh was named 1 of 10 fast-track countries for achievement of MNCH Millennium Development Goals included in a comparative analysis conducted in 2016, due to large-scale implementation of integrated strategies for MNCH care delivery [20]. These integrated programs include several large-scale maternal and child nutrition interventions [23–26]. However, of the maternal nutrition interventions implemented in Bangladesh to date, very few have included maternal complications as an outcome measure [8,22]. Further, a recent report found that MMR has plateaued over the last 6 years in Bangladesh and that hemorrhage and hypertensive disorders remain the main causes of maternal mortality [27], suggesting that programmatic approaches resulting in the initial substantive decrease may need to be supplemented by other actions to further reduce maternal mortality and morbidity.

Several large-scale maternal nutrition interventions have been conducted in Bangladesh in the last decade, with most focusing on neonatal and child nutrition and developmental outcomes. We present an analysis nested within one such study, a maternal nutrition intervention [28–32]. The parent intervention combined intensive antenatal and postpartum nutrition counseling, home visits by community-level health workers and volunteers, weight monitoring, and provision of calcium and iron/folic acid (IFA) supplements with a community-based MNCH program operated by BRAC in 10 *upazilas* (sub-districts) of 4 districts of Bangladesh. Findings from this parent study are notable for increased maternal supplement intake, dietary diversity, and exclusive breastfeeding rates [28]; greater engagement by husbands in nutrition and food selection for their wives [29]; and reduced household food insecurity [30]. The parent study endline report also found significantly increased maternal knowledge between cross-sectional baseline and endline assessments of pregnant women or women with an infant less than 6 months of age regarding reasons to take calcium and IFA supplements, specifically as a means of preventing maternal complications like pre-eclampsia/eclampsia and anemia [32]. However, these parent study assessments did not include maternal obstetric complications as an outcome measure. The purpose of this nested analysis was to compare rates of reported antepartum, intrapartum, and postpartum maternal morbidities between postpartum women residing in an area exposed to the parent intervention and those residing in control areas in 4 selected districts (Mymensingh, Rangpur, Lalmonirhat, and Kurigram) of Bangladesh.

## Methods

### Parent intervention

The parent maternal nutrition intervention, Alive & Thrive II, has been previously described in several publications [28–32]. Briefly, the primary objective of the parent intervention was to demonstrate the feasibility of integrating a package of maternal nutrition interventions into a large-scale community-based MNCH program. The community-based MNCH program has trained salaried health workers (Shasthya Kormi [SKs]) who conduct ANC visits at least monthly and 4 PNC visits at the household level. The SKs motivate pregnant women and their families to seek facility-based delivery, sell micronutrient powders for children, provide supplements for pregnant and lactating women, and provide facility referrals for testing and suspected complications throughout the pregnancy and postpartum period. These visits are augmented by visits from the volunteer community health workers (Shasthya Shebika [SSs]), a cadre chosen from and by their communities who receive a 15-day training course followed by monthly refresher training and ad hoc 3-day intensive training for new content areas. These workers have a catchment area of 150 households, and are largely responsible for home-based counseling, ensuring nutrient intake in meals and intake of micronutrient supplements, identification of any pregnancy-related problems and referral for facility-based care, and referrals for care by facility-based or community providers. The SS engages women at the household level in her community and reports pregnancies following urine rapid test screening to the SK for home-based care and also provides facility referrals. The parent intervention concentrated on improving dietary practices, specifically dietary diversity and energy content of daily meals for pregnant women, and improved intake of calcium and IFA supplements. For the intervention, SKs working in the intervention clusters received additional training in nutrition and supplement counseling, measurement of cooked food, how to select diverse foods from available sources, and conducting pill counts. The parent trial conducted 2 rounds of sampling approximately 1 year apart with cross-sectional samples of eligible women, i.e., women who were pregnant or with a child less than 6 months of age. SKs in intervention and control areas did not differ based on level of training prior to the parent trial. In addition to the supplemental training in the intervention areas, SKs in those areas were expected to administer food recall questionnaires, provide IFA and calcium supplements, and conduct intensive nutrition counseling during their home visits, in addition to routine SK maternal and newborn healthcare. There was no difference in salary between intervention and control area SKs. To realize these objectives, the intervention added the following components to BRAC’s community-based MNCH program:

- intensified counseling with emphasis on coaching and demonstration of actual food preparation by SKs and SSs; SSs were incentivized to conduct follow up counseling;
- adequate supply of free IFA and calcium tablets provided by BRAC, which SKs delivered each month during the ANC and PNC services provided at the houses of pregnant or delivered women; compliance with taking supplements was emphasized through home visits and husband mobilization;
- strengthened supervision, monitoring, and problem-solving by SKs and SSs regarding proper nutrition intake during pregnancy and the postpartum period, early initiation of breastfeeding, and exclusive breastfeeding;
- community-based husbands’ forums held for male mobilization; and
- interactive media events conducted for the entire community, using rural activation methods to change misperceptions about maternal nutrition and promote model family and community support for pregnant women.

### Study design and participants

This cross-sectional study was nested within the parent cluster-randomized trial (NCT02745249), with sampling of women divided equally between 10 randomly selected intervention and 10 control sub-district clusters determined by the parent intervention trial in Mymensingh, Rangpur, Kurigram, and Lalmonirhat districts. Data were collected from a non-probabilistic sample of postpartum women meeting eligibility criteria between January and March 2017, approximately 6 months after the endline survey for the parent study (please see S1 STROBE Checklist). Two SKs were purposefully selected from each of the sub-districts based on high client volume to introduce study staff during PNC visits for participant recruitment. Eligible participants were Bangla-speaking women who received ANC from selected SKs living in the designated SK catchment area and delivering within the last 42 to 60 days. We constrained eligibility at 42 to 60 days postpartum for 2 reasons. First, PNC visits were scheduled within 48 hours of delivery and then at 7, 28, and 42 days; the eligibility range was meant to synchronize with the last PNC visit. Early (<42 days) PNC visits often do not occur as, traditionally, many women choose to deliver in their mother’s home and are not accessible for PNC visit access until after 40 days postpartum. Second, we ended the eligibility period at 60 days to allow extra time to complete the 42-day visit but to limit the interval since birth to reduce recall bias when eliciting and validating complication reports.

### Measures

We collected data for this assessment predominantly from 3 sources: direct interview with postpartum women, abstraction of data from the participant MNCH handbook (paper records), and abstraction of data from the SK register (S1 Study Instruments).

A questionnaire was developed and pretested in sub-districts not involved in the study. For the instrument pretest, field staff met with the SK for the selected non-study area, who introduced the staff at homes of postpartum women. Field staff obtained verbal consent to administer the study instrument to approximately 20 volunteers, who, in addition to answering the study instrument questions, were asked to advise if questions were difficult to understand or too sensitive to answer, if they became fatigued during the interview, or periodically how they interpreted a specific question. Field staff similarly tested the abstraction form with the SK register of the SK serving the selected pretest area. These inputs were recorded and discussed following pretesting to refine and produce the final instrument versions.

We asked intervention and control participants about presence of and specific type of any antepartum, intrapartum, and postpartum complications they had experienced during their index pregnancy. We also gauged severity of the complication by asking whether care at a health facility was sought, whether the participant was hospitalized, and about receipt of either a transfusion or medication for the event. All complications were elicited as an open-ended question. Frequently named complications during the pretest were compiled into a list for ease of entry, but field staff did not read the list to or prompt participants. We collected data regarding socioeconomic and household economic characteristics, participant height, and a detailed medical and obstetric history, particularly on complications in pregnancies prior to the index pregnancy through participant report. In sub-districts randomized to the control condition, we measured nutrition adequacy with reported 24-hour food recall and IFA/calcium supplement intake through participant interview. We did not inquire about dietary intake measures or IFA or calcium intake among intervention group participants during participant interview due to concerns about socially desirable response, as these measures were routinely queried by SKs during home visits. We obtained data on dietary diversity scores and supplement consumption among intervention participants from the SK register: We abstracted data for dietary diversity scores from the first and last ANC visits and from the most recent PNC visit from the SK register during the single household visit at which the woman consented and completed the interview.

The data abstraction form was developed to record information directly from the patient’s booklet (MNCH handbook), the SK register, the hospital certificate, and, in the event of a maternal death, a verbal autopsy report. Maternal deaths occurring before 60 days postpartum were specifically queried by requesting BRAC field officers to review monthly records from the SK catchment areas included in this assessment during the data collection period. The BRAC program has a Maternal and Perinatal Death Surveillance and Response committee in each sub-district office that identifies, investigates, and presents case facts and contributing factors for maternal deaths to field and facility staff as part of care quality improvement activities. Maternal deaths reported during the data collection period were identified first through this mechanism and then corroborated through the SK register and facility record, as applicable. The MNCH handbook, developed by BRAC, is provided to pregnant women at the time they register with SKs for home-based care. The MNCH handbook has a dual purpose: recording physical examination results and providing pictorial information on pregnancy, delivery and postpartum care, newborn care, and child care, which the SK uses to counsel pregnant and postpartum women. For this study, key messages on maternal nutrition were incorporated into the MNCH handbook for intervention areas, while women in control areas received the original version. The handbook also contains laboratory measures, medical history, and any hospital referrals. It stays with the patient, serving as a record to ensure that providers from different service points (e.g., home, hospital, clinic) are able to know what care was provided since their last encounter with the patient. The handbook may contain a hospital certificate, which is issued at discharge and contains information from facility-based care, including birth weight and any complications for births. The SK register is a record-keeping book that tracks examination findings and key health information (e.g., blood pressure at each visit, dietary diversity measures in intervention areas) at all household visits. The SK should always possess this register and carry it into the field for direct data entry. In practice, the registers are sometimes kept at home by the SK for data entry at the end of the day; validating data entry is one purpose of this assessment. Last, in the event of a reported maternal death, verbal autopsy reports completed by BRAC medical officers were accessed and reviewed from BRAC area offices.

**Fig 1:**
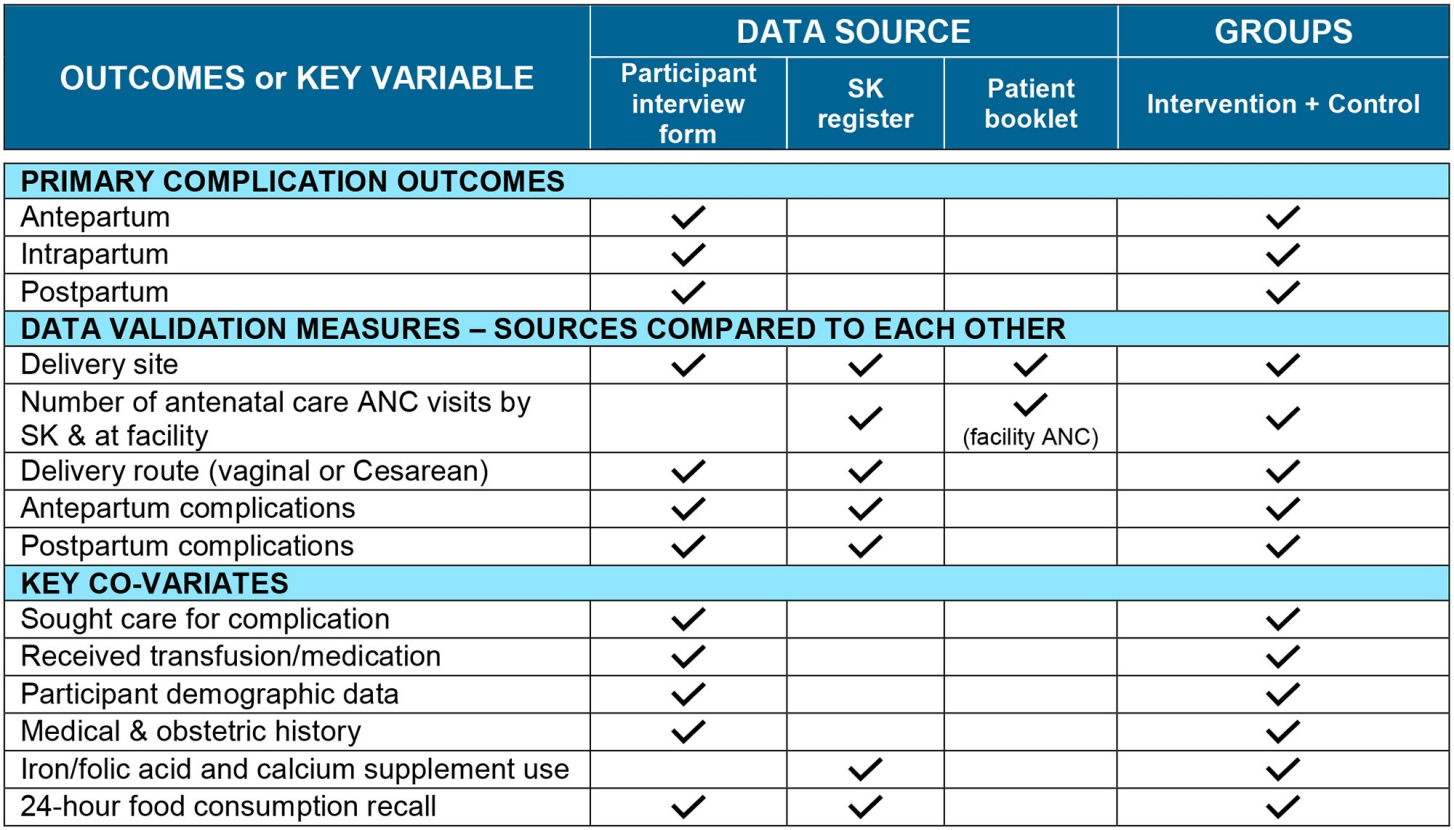
Data sources used for primary outcomes and key variables among postpartum women, in 4 districts of Bangladesh. ANC, antenatal care; SK, Shasthya Kormi.

We made a concerted effort to avoid extensive duplication of data routinely recorded in written records within the interview instrument, to ease respondent burden. However, we did collect duplicate information for several key indicators (e.g., date of delivery) between the 2 data sources. The principal reasons for this duplication were to determine the level of recording of important information (e.g., maternal complications) in written records by SKs and other providers and to determine the agreement between the 3 sources: the participant interview, the SK register, and the MNCH booklet. The rationale for this comparison was to determine whether and to what degree SKs are collecting complete information, as their registers are used to determine program performance and make decisions regarding design of and implementation guidelines for the community MNCH program. The data abstraction form collected participant weight and blood pressure values at first and last ANC visits, date and blood pressure at first PNC visit, any reported complications, any referrals or hospitalizations for complications in pregnancy or the postpartum period, date and details (e.g., location, skilled attendant, birthweight) of delivery, IFA and calcium supplement intake, and, in intervention areas, recorded dietary diversity by 24-hour recall. To ensure clarity during data interpretation, Fig 1 illustrates data sources for the outcome measures for both groups and for variables for which sources differed between intervention and control group.

All data collection instruments were translated into Bengali and pretested with female volunteers in an area not involved in the study.

### Data collection procedures

Female research assistants (RAs) were trained in human participant research, informed consent, questionnaire administration, and health booklet audit and data recording in both classroom and field settings prior to initiating data collection. BRAC MNCH program health officers introduced the RAs to the designated SKs serving a specified sub-district during the pretesting activities. Each RA then provided information about the study to the SK and requested permission to accompany the SK to see women at a PNC visit between 42 and 60 days postpartum and to review the SK’s register and the woman’s MNCH handbook, the patient medical record. All selected SKs assented to having RAs accompany them to the field and offer potential participants enrollment. To reduce time burden, each SK identified women in her catchment area meeting the 42- to 60-day postpartum criterion and asked the SS in that village to introduce the RA to the family in their home. Upon reaching each household with a postpartum mother, the SK or SS, as available, introduced the RA to the woman and her family. Following introductions, the RA provided a brief study overview and, if the woman was interested, obtained witnessed verbal informed consent.

RAs verbally administered the questionnaire form to participants in a private location within the participant’s home. Participants were asked if they had any questions regarding health issues raised in the questionnaire and, if so, were counseled to re-engage the SK or SS for care-related queries or needs. The RA then asked to see the participant’s MNCH handbook, SK register, and any hospital certificates, as applicable, to complete the abstraction form.

If the SK register was located off-site or the SK was absent, the RA met the SK at her home or at the BRAC office at the completion of that working day and completed the abstraction form for the corresponding participant at that time. All information added to data collection forms was taken from written records or directly from participant interview.

### Analysis

We estimated the probable number of postpartum women available for enrollment based on SK register data as approximately 5 women delivered in each SK catchment area/month in the quarter prior to protocol development. For the parent study coverage areas, we estimated 600 postpartum women could be enrolled in each study arm area (10 *upazilas*/study arm area × 5 deliveries/month/SK × 2 SKs/*upazila* × 6 months of data collection). Using the baseline cesarean section rate of 22.3% as a proxy for intrapartum complications [28], and assuming an intraclass correlation coefficient of 0.1 at the *upazila* level, we estimated that we would have 80% power to detect a 17-percentage-point difference between the intervention and control study groups with 600 women per study group.

Data were entered into a SPSS version 24.0 (IBM, Armonk, NY, US) database with internal parameters for error prevention. Following validation checks, 5% of data were double-entered for quality assurance. After data entry was completed, a detailed round of data cleaning and verification was done in Dhaka, with validation assessment and coding, prior to analysis in North Carolina. Regarding missing data, we dropped any cases missing the main outcome from the analysis and, for the regression models, we also dropped any cases with missing values in the covariates. In doing so, we tracked the percentage of cases lost due to missing data, which did not exceed 5%.

Data analysis was conducted in SAS version 9.40 (SAS Institute, Cary, NC, US) guided by a statistical analysis plan developed prior to data collection. We conducted a descriptive analysis of all participants and studied the differences between the intervention and control groups, accounting for the clustering of women within *upazilas*, and controlling for some key covariates. Statistical testing for differences between the 2 groups was done using chi-squared test, *t* test, or ANOVA. Participants in the intervention and control groups were compared across a series of variables based on participant report, including percentage who had any complication, anemia, severe vomiting, severe edema, high blood pressure, vaginal bleeding, pre-eclampsia, and diabetes. Comparisons were made separately for antepartum, intrapartum, and postpartum complications, with most analyses confined to a composite variable of “any complication” for each of the 3 time periods. We also studied the association between having a complication in each of the time periods and the intervention, after controlling for key respondent characteristics including obstetric/medical history and socioeconomic status. For each outcome, we used a hierarchical logistic model (also known as a multilevel logistic model). This modeling approach is needed to account for the dichotomous outcome and for the non-independence of observations from the same *upazila* (i.e., to account for the clustering of observations within *upazilas*). The first model (crude model) included an indicator only for exposure. In the second model (adjusted model), we added the medical history and socioeconomic variables associated with the complication either empirically (e.g., age, parity) or in bivariate analysis (e.g., complications in a prior pregnancy).

Differences between routine care measures by participant report and recorded information in the SK register were measured using the Kappa statistic for both intervention and control participants.

### Ethical approvals

Ethical approval was obtained from the James P Grant School of Public Health Ethical Review Committee, BRAC University, in Bangladesh, and the Protection for Human Subjects Committee, FHI 360, in the US, prior to any data collection. Witnessed verbal informed consent was obtained from all women to participate in the study.

## Results

### Participant characteristics

We enrolled 596 (99.3% of planned sample size) intervention and 507 (84.5% of planned sample size) control participants in this cross-sectional study during the approximately 3-month data collection period (Table 1). No women approached for study entry declined participation. Women from the intervention and control areas had similar time since delivery and similar socioeconomic features (Table 1), with high rates of mobile phone and home ownership.

**Table 1:** Participant household and socioeconomic characteristics from participant interviews in postpartum women from intervention and control areas in 4 districts of Bangladesh.

Characteristic	Intervention (*n* = 596)	Control (*n* = 507)	*p*-Value*
Age, years^†^			0.38
Mean (SE)	24.0 (0.33)	23.7 (0.27)	
Timing of interview, days postpartum			0.76
Mean (SE)	51.5 (0.62)	51.3 (0.50)	
Number in household^†^			0.07
Mean (SE)	5.2 (0.1)	5.0 (0.1)	
Monthly household income, taka			0.48
Mean (SE)	6,552.0 (647.83)	6,016.8 (379.92)	
Source of drinking water			0.61
Own tube well	516 (86.6%)	421 (83.0%)	
Other’s tube well	69 (11.6%)	66 (13.0%)	
Other	11 (1.8%)	20 (3.9%)	
Property ownership			
Own house	550 (92.3%)	461 (90.9%)	0.78
Own land	259 (43.5%)	226 (44.6%)	0.86
Household has electricity			
Yes	415 (69.6%)	362 (71.4%)	0.84
Main floor material			0.46
Concrete	50 (8.4%)	27 (5.3%)	
Brick/cement	49 (8.2%)	38 (7.5%)	
Dirt	497 (83.4%)	442 (87.2%)	
Household assets			
Television	245 (41.1%)	196 (38.7%)	0.81
Bicycle	230 (38.6%)	218 (43.0%)	0.66
Motorcycle	46 (7.7%)	29 (5.7%)	0.24
Mobile phone	555 (93.1%)	474 (93.5%)	0.79
Cow/buffalo	241 (40.4%)	223 (44.0%)	0.60
Chicken/duck	426 (71.5%)	345 (68.0%)	0.52

*Accounts for clustering at *upazila* level.

^†^For age and number in household: intervention *n* = 595, control *n* = 504.

In this cohort, approximately one-third of participants were primiparas (the index pregnancy being their first delivery), with no significant difference between intervention and control groups. Participants reported general good health prior to the index pregnancy, with about 17% reporting any prior health problem, with similar prevalence between the intervention and control groups. The most commonly reported preexisting condition was anemia. Specific conditions did not differ significantly between the intervention and control groups, with the exception of heart disease and malnutrition, which were more commonly reported among control area participants, with malnutrition being the second most common reported preexisting medical condition in this group (Table 2).

**Table 2:** General and reproductive health history and co-morbid conditions from participant interviews (except where noted) in postpartum women from intervention and control areas in 4 districts of Bangladesh.

Variable	Intervention	Control	*p*-Value*
Height^†^			0.90
Mean (SE) (meters)	1.5 (0.01)	1.5 (0.01)	
Total *n*	596	507	
BMI at first ANC visit^‡^ (*n* = 588)			—
Mean (SE) (kg/m^2^)	20.5 (0.32)	—	
<18.5 kg/m^2^	171 (29.1%)	—	
18.5 to <25 kg/m^2^	363 (61.7%)	—	
≥25 kg/m^2^	54 (9.2%)	—	
Number of pregnancies^†^			0.45
Mean (SE)	2.4 (0.09)	2.3 (0.09)	
1	172 (29.0%)	170 (33.6%)	
2	202 (34.0%)	147 (29.1%)	
3	121 (20.4%)	116 (22.9%)	
>4	99 (16.7%)	73 (14.4%)	
Number of prior term deliveries^†^			0.19
0	11 (1.9%)	20 (4.0%)	
1	214 (36.0%)	186 (36.8%)	
2	205 (34.5%)	170 (33.6%)	
3	104 (17.5%)	93 (18.4%)	
>4	60 (10.1%)	37 (7.3%)	
Number of prior preterm deliveries^†^			0.82
0	511 (86.0%)	446 (88.1%)	
1	65 (10.9%)	46 (9.1%)	
2	13 (2.2%)	12 (2.4%)	
>3	5 (0.9%)	2 (0.4%)	
Number of prior pregnancy losses at <20 weeks^†^			0.35
0	508 (85.5%)	416 (82.2%)	
1	73 (12.3%)	77 (15.2%)	
>2	13 (2.2%)	13 (2.6%)	
Number of living children^†^			0.33
Mean (SE)	2.0 (0.07)	1.9 (0.08)	
0	4 (0.7%)	3 (0.6%)	
1	214 (36.0%)	193 (38.1%)	
2	216 (36.4%)	187 (37.0%)	
3	103 (17.3%)	94 (18.6%)	
>4	57 (9.6%)	29 (5.7%)	
Participants reporting medical conditions prior to pregnancy, percent (95% CI)			0.34
Any condition	15.0 (9.7–20.2)	18.6 (12.5–24.6)	
Diabetes	1.2 (0.7–1.7)	0.4 (0.0–0.9)	0.07
High blood pressure	0.8 (0.1–1.6)	0.6 (0.0–1.2)	0.58
Malnutrition	1.8 (1.0–2.7)	5.1 (1.6–8.7)	<0.01
Underweight	0.7 (0.0–1.4)	0.6 (0.0–1.2)	0.86
Short stature	1.3 (0.2–2.5)	2.4 (1.1–3.7)	0.20
Asthma	2.4 (0.5–4.2)	2.4 (0.3–4.4)	0.99
Anemia	5.7 (0.1–11.3)	6.5 (0.8–12.2)	0.83
Heart condition	0.2 (0.0–0.5)	1.2 (0.0–2.4)	0.01

Data given as number (percent), unless otherwise indicated.

*Accounts for clustering at *upazila* level.

^†^Intervention *n* = 594 with information from participant interview.

^‡^Intervention BMI *n* = 588 women, based on weight at first ANC visit recorded in SK register; only 4 control participants had weight recorded, so these data are not presented.

ANC, antenatal care; CI, confidence interval; SE, standard error.

### Care utilization during index pregnancy and delivery

All participants reported having at least 1 ANC visit with a SK, though 26% of women reported no facility-based visits, with no significant difference between intervention and control sites (22.3% versus 29.7%, *p* = 0.48; Table 3). Nearly half (42%) of participants reported having their first ANC visit at or before 13 weeks gestation; first trimester ANC initiation did not differ between intervention and control areas. In the seventh and eighth months of pregnancy, nearly all women reported having a visit from the SK, while about half reported a visit from the SS. Overall, participants reported an average of 7.3 SK and 9.8 SS visits through the antepartum and postpartum periods. Women living in intervention sub-districts had significantly more visits, both in number and in critical periods (seventh–eighth month), from community-based providers than did women living in the control areas (Table 3). Most (81.2%) participants planned a home delivery, and nearly all (98.3%) reported having a birth plan.

**Table 3:** Index pregnancy care and delivery from participant interviews (except as noted) in postpartum women from intervention and control areas in 4 districts of Bangladesh.

Variable	Intervention	Control	*p*-Value*
Gestational age at delivery (weeks)	39.4 (0.29)	40.1 (0.32)	0.11
Gestational age at first ANC visit (weeks)^†^	15.9 (0.50)	16.7 (0.65)	0.40
Number of ANC visits at facility^‡^	1.6 (0.17)	1.4 (0.19)	0.54
Number of ANC visits by SK^†^	5.4 (0.12)	5.2 (0.17)	0.28
Total number of SK visits (ANC + PNC)**	7.7 (0.16)	6.9 (0.24)	0.02
Gestational age at delivery (weeks)			0.06
<34	5.4% (32)	4.2% (21)	
34 to <37	12.9% (76)	13.1% (66)	
37 to 42	74.1% (438)	69.6% (351)	
>42	7.6% (45)	13.1% (66)	
Proportion of women having SK visit in 7th–8th month of pregnancy	95.3% (594)	93.5% (506)	0.54
Proportion of women having SS visit in 7th–8th month of pregnancy	63.5% (595)	36.8% (506)	<0.01
Reported having birth plan	99.2% (594)	97.2% (506)	0.04
Planned site of delivery	(*n* = 591)	(*n* = 501)	0.34
Home	79.9%	82.8%	
Hospital/health facility	18.8%	13.8%	
Other	1.4%	3.4%	
Actual site of delivery	(*n* = 594)	(*n* = 506)	0.01
Home	57.2%	62.3%	
Hospital/health facility	40.2%	33.0%	
Other	2.6%	4.7%	
Delivery route	(*n* = 594)	(*n* = 506)	0.55
Vaginal	75.1%	75.9%	
Cesarean section	24.6%	22.9%	
Assisted vaginal	0.3%	1.2%	

Data given as mean (SE) or percent (*n*).

*Comparing intervention and control areas; accounts for clustering at *upazila* level.

^†^Abstraction form data from SK register: 595 intervention participants, 504 control participants.

^‡^Abstraction form data from MNCH handbook: 358 intervention participants, 316 control participants.

**Interview: 594 intervention participants, 506 control participants.

ANC, antenatal care; MNCH, maternal, newborn, and child health; PNC, postnatal care; SK, Shasthya Kormi; SS, Shasthya Shebika.

While only 16.5% of women planned to deliver at a health facility, 36.9% delivered at a facility, and a further 0.6% delivered en route to a facility. Women living in control areas were more likely to both plan and deliver at home, with intervention area participants significantly more likely to deliver in facilities (Table 3). Overall, labor lasted approximately 6 hours, with no significant difference between intervention and control areas (S1 Fig; *p* = 0.68). One-quarter (23.8%) of participants delivered by cesarean section, with half (55.6%) reporting medical indications as the reason and a further 35.7% reporting doctor recommendation as the reason. Cesarean section rates did not differ between intervention and control areas (Table 3). Skilled attendance at birth (58.2% for intervention group and 50.5% for control group; *p* = 0.28) and misoprostol receipt for PPH prevention (34.6% for intervention group and 27.6% for control group; *p* = 0.24) did not differ significantly, while postpartum vitamin A administration (42.1% for intervention group and 29.8% for control group; *p* = 0.03) significantly differed between women in intervention and control areas.

### Difference in intervention-specific process indicators

As part of the intervention, women received IFA and calcium supplements delivered by SKs through pregnancy, while women in control areas were encouraged to procure and use these supplements. Supplement use was recorded within the SK register and demonstrated a downward trajectory in recording levels longitudinally through the pregnancy and postpartum period, based on numbers contributing data (S2 and S3 Figs). This was particularly true in the control areas, where the number with recorded data went from 370 to 162 over the antepartum through postpartum periods. Mean supplement intake was consistently and significantly higher among participants in the intervention areas in all time periods (*p <* 0.01 for comparisons at all time points) (S3 and S4 Figs).

Women in intervention areas were queried about dietary intake using 24-hour food consumption recall at routine SK visits as part of intensified nutrition counseling, while women in control areas were asked about dietary intake only within this assessment. As such, data for each group come from separate sources (SK register for participants in the intervention group, and SK inquiry at last visit and direct inquiry by the RA for the control group). For women in intervention areas, reported animal-source protein and micronutrient-rich vegetable and fruit intake were labile, with highest intake noted around the time of delivery and with antepartum and postpartum levels roughly equivalent (Table 4). Starch consumption remained relatively consistent throughout the recording period and was similarly high among control area participants. By contrast, lower proportions of control area participants reported animal-source protein and vegetable/fruit consumption in the postpartum period, and these women had a lower mean dietary diversity score (*p <* 0.001). We did not record the date at which the antepartum and postpartum dietary diversity scores for the intervention group were recorded by the SK and thus cannot comment on seasonal variation. Most intervention participants had had only 1 postnatal visit by the SK at the time of study data collection, and we recorded the most recent postnatal dietary diversity score if more than 1 visit had been made at the time of data collection.

**Table 4:** Maternal dietary intake in pregnancy and postpartum period among postpartum women from intervention (SK register data) and control (participant interview data) areas in 4 Bangladesh districts.

Dietary intake	Intervention group (source: SK register)			Control group (source: postpartum interview)
	First ANC visit	Last ANC visit	Last recorded PNC entry*	
Food groups, *n* (percent)				
Fish/meat/liver	555 (96.2%)	562 (98.8%)	503 (92.0%)	357 (71.3%)
Egg	385 (66.7%)	543 (95.4%)	357 (65.4%)	154 (30.8%)
Milk/milk products	345 (59.8%)	486 (85.4%)	286 (52.3%)	91 (18.2%)
Orange/yellow vegetables/fruit	352 (61.0%)	462 (81.2%)	311 (56.9%)	200 (40.0%)
Rice/bread	573 (99.3%)	569 (100%)	542 (99.1%)	497 (99.4%)
Dark green vegetables	444 (76.9%)	517 (90.9%)	423 (77.3%)	276 (55.2%)
Thick lentils	302 (52.3%)	442 (77.8%)	298 (54.6%)	169 (33.9%)
Number of dietary groups consumed	(*n* = 577)	(*n* = 569)	(*n* = 547)	(*n* = 501)
Mean (SE)	5.1 (0.25)	6.3 (0.17)	5.0 (0.18)	3.5 (0.12)
Median (IQR)	4.6 (3.4–6.0)	6.2 (5.3–6.6)	4.6 (3.2–5.9)	3.0 (2.1–3.9)

*PNC visits by SKs for intervention participants where dietary diversity was measured generally occurred within 60 days of the same data collection conducted by study staff for the control group. We did not assess seasonal variation for ANC or PNC visit dietary diversity data.

ANC, antenatal care; IQR, interquartile range; PNC, postnatal care; SE, standard error; SK, Shasthya Kormi.

### Comparison of antepartum complications

Of 1,100 participants responding, 74% overall reported some type of antepartum pregnancy complication, with reported complications more common among women in control areas (Table 5). Vomiting, back pain, fever, preterm labor, and anemia were the most commonly reported antepartum complications; of these, preterm labor was significantly more likely among women in control areas. Antepartum complications resulted in hospital visits or hospitalization about half of the time, with no difference between the intervention and control groups (Table 5).

**Table 5:** Differences in proportion and type of antepartum complications reported in participant interviews of postpartum women from intervention and control areas in 4 districts of Bangladesh.

Antepartum complications	Intervention	Control	*p*-Value*
Women who had complications during pregnancy before going into labor	(*n* = 594)	(*n* = 506)	
Any complication	69.4 (56.6–82.1)	79.2 (72.4–86.1)	0.12
Anemia	21.0 (3.0–39.1)	19.2 (5.3–33.0)	0.86
Severe vomiting	47.0 (31.8–62.1)	49.4 (36.3–62.5)	0.80
Severe edema	15.3 (10.1–20.5)	18.0 (12.5–23.4)	0.45
High blood pressure	3.2 (1.5–4.9)	3.6 (1.7–5.4)	0.77
Vaginal bleeding	3.9 (0.8–6.9)	4.5 (2.1–7.0)	0.72
Pre-eclampsia	0.3 (0.0–0.8)	2.0 (0.0–4.4)	0.01
Diabetes	0.8 (0.0–1.9)	1.4 (0.0–2.7)	0.51
High fever	15.0 (5.3–24.6)	20.9 (7.7–34.2)	0.43
Preterm labor	16.4 (7.4–25.3)	30.6 (17.4–43.9)	0.03
Low weight gain	1.5 (0.6–2.5)	1.6 (0.2–3.0)	0.94
Fluid leaking	10.6 (6.6–14.6)	15.6 (9.8–21.4)	0.11
Back pain	24.2 (14.6–33.9)	34.8 (19.9–49.6)	0.19
Abdominal pain	4.2 (1.4–7.0)	4.7 (1.9–7.6)	0.78
Problem with uterine position	0.8 (0.0–1.7)	1.0 (0.0–2.1)	0.82
Malnutrition	0.2 (0.0–0.5)	1.6 (0.3–2.9)	<0.01
Other complication	6.6 (3.2–9.9)	8.1 (3.0–13.2)	0.58
Treatments due to severity of condition (of women reporting complications)	(*n* = 410)	(*n* = 397)	
Hospital visit/admission	50.0 (43.6–56.0)	45.1 (35.8–54.4)	0.37
Of those seeking care at facility	(*n* = 202)	(*n* = 179)	
Received transfusion	0.5 (0.0–1.5)	0	
Received medicine	95.0 (91.7–98.4)	94.4 (90.0–98.8)	0.80

Data given as percent (95% CI), unless otherwise indicated.

*Accounts for clustering at *upazila* level.

A hierarchical logistic regression model did not reveal a statistically significant association between reported antepartum complications of any type and study group, though the intervention group reported about 9 percentage points fewer complications of any type (S1 Table). No significant association between reported antepartum complications and study group emerged after controlling for key variables such as district, age, reported malnutrition, history of prior complications, and select socioeconomic variables.

### Comparison of intrapartum complications

The intrapartum complications described here refer to events occurring during labor and delivery, reflecting the question posed to participants. About 45% of all participants reported intrapartum complications. Referrals to a health facility likely occurred during labor, as about 65% of those experiencing complications reported being seen at and 60% delivered at a health facility due to the reported complication. While women living in control areas were more likely to report an intrapartum complication, they were significantly more likely to deliver at home and not have a skilled birth attendant (e.g., doctor, midwife) present for the delivery. Women in control areas were also less likely to go to a health facility or receive medication upon experiencing an intrapartum complication (Table 6).

**Table 6:** Differences in proportion and type of intrapartum complications reported in participant interviews of postpartum women from intervention and control areas in 4 districts of Bangladesh.

Intrapartum complications	Intervention	Control	*p*-Value*
Women who had complications during labor and delivery	(*n* = 594)	(*n* = 506)	
Any complication	41.4 (34.1–48.7)	48.5 (39.6–57.4)	0.18
Pre-eclampsia	2.9 (0.6–5.1)	2.4 (0.8–3.9)	0.70
Eclampsia	0.5 (0.0–1.3)	1.4 (0.3–2.4)	0.16
Heavy bleeding	4.2 (1.0–7.5)	6.7 (0.0–15.0)	0.49
Retained placenta	3.0 (1.5–4.5)	7.5 (4.1–11.0)	<0.01
Prolonged labor	23.2 (14.2–32.2)	24.8 (18.6–31.0)	0.77
Obstructed labor	10.1 (4.9–15.3)	11.7 (6.1–17.2)	0.66
Fever/Infections	1.5 (0.3–2.8)	5.1 (2.2–8.1)	<0.01
Breech/non-cephalic presentation	3.0 (1.4–4.7)	4.6 (1.6–7.5)	0.29
Fluid leak	4.0 (2.2–5.8)	6.5 (3.3–9.8)	0.10
Other complication	6.2 (2.6–9.8)	6.3 (3.0–9.6)	0.96
Treatments due to severity of condition (of women reporting complications)	(*n* = 246)	(*n* = 244)	
Went to hospital	69.9 (60.8–79.0)	59.8 (45.8–73.9)	0.18
Delivered in health facility	62.2 (52.5–71.9)	52.7 (38.7–66.6)	0.22
Of those seeking care at a facility	(*n* = 172)	(*n* = 146)	
Received transfusion	8.1 (2.3–14.0)	7.5 (3.2–11.9)	0.86
Received medicine	98.3 (95.5–100)	93.2 (85.2–100)	0.08

Data given as percent (95% CI).

*Accounts for clustering at *upazila* level.

While there were just 2 types of reported intrapartum complications (retained placenta and fever/infection) significantly higher among women in the control group, most complications were more prevalent among women in control areas but by a degree that did not attain statistical significance. We fit a series of hierarchical logistic regression models to further explore the association between the nutrition intervention and intrapartum complications overall and for retained placenta and fever/infection (S2–S4 Tables). Women in the intervention group (41.4% versus 48.5% in the control group) reported fewer intrapartum complications overall; however, the difference is not statistically significant in crude analysis or in adjusted analysis controlling for key variables. We found a statistically significant association between intervention exposure and reduced odds of reported retained placenta in both crude (odds ratio [OR] = 0.39, 95% CI 0.20–0.78) and adjusted (adjusted OR [AOR] = 0.35, 95% CI 0.19–0.67) models (S3 Table). We also found that hospital delivery statistically significantly decreased the odds of reporting retained placenta by 80%. We also a found significantly lower odds of reporting intrapartum fever or infection among women exposed to the intervention in crude (OR = 0.27, 95% CI 0.09–0.79) and adjusted (AOR = 0.27, 95% CI 0.11–0.65) models (S4 Table). For intrapartum fever, hospital delivery was not significantly associated with the outcome, but there were some statistically significant differences by district (S4 Table).

### Comparison of postpartum complications

Postpartum complications were reported by 40% of participants, with the most common complications being weakness (characterized as a complication by participants), abdominal pain, fever/infection, and bleeding after delivery (Table 7). These reported complications, both overall and for most specific conditions, were more common among women in control areas, with differences for multiple specific conditions reaching statistical significance in bivariate analysis. About one-third of participants reporting these complications sought care at a health facility, and about 10% received a transfusion as part of their treatment.

**Table 7:** Differences in proportion and type of postpartum complications reported in participant interviews of women from intervention and control areas in 4 districts of Bangladesh.

Postpartum complications	Intervention	Control	*p*-Value*
Women who had complications after delivery	(*n* = 594)	(*n* = 506)	
Any complications	33.5 (25.1–41.9)	48.2 (36.7–59.7)	0.02
Eclampsia	1.7 (0.0–3.4)	2.2 (1.5–2.9)	0.61
Bleeding after delivery	7.9 (3.9–11.9)	17.0 (5.7–28.2)	0.04
Fever/infection	13.3 (7.8–18.9)	19.8 (9.1–30.4)	0.22
Abdominal pain	15.5 (9.8–21.2)	26.5 (13.7–39.3)	0.05
Weakness	24.5 (16.4–32.5)	36.4 (24.8–48.0)	0.06
Edema	0.5 (0.0–1.2)	2.0 (0.6–3.3)	0.03
High blood pressure	1.0 (0.0–2.1)	0.6 (0.0–1.5)	0.53
Other complication	2.7 (0.0–5.7)	2.6 (0.6–4.6)	0.94
Treatments due to severity of condition (of women reporting complications)	(*n* = 199)	(*n* = 244)	
Went to hospital	36.2 (23.6–48.7)	30.7 (15.9–45.5)	0.49
Of those seeking care at a facility	(*n* = 72)	(*n* = 75)	
Received transfusion	9.7 (0.0–21.9)	8.0 (0.0–18.5)	0.83
Received medicine	98.6 (95.7–100)	96.0 (93.0–99.0)	0.71

Data given as percent (95% CI).

*Accounts for clustering at *upazila* level.

In hierarchical logistic regression modeling, we found that women in the intervention group had half the odds of reporting postpartum complications overall in crude (OR = 0.50, 95% CI 0.29–0.88) and adjusted analyses (AOR = 0.51, 95% CI 0.32–0.82; controlling for district, age, reported malnutrition, prior pregnancy complications, and select socioeconomic variables) (S5 Table). Among specific complications, reported PPH was lower for women exposed to the intervention in the crude model (OR = 0.41, 95% CI 0.14–1.25) and was significantly lower in the model adjusted for multiple factors, including retained placenta (AOR = 0.37, 95% CI 0.15–0.92) (S6 Table). Of note, individuals reporting any intrapartum complication were more than 5 times more likely to also report PPH.

There were 3 cases of maternal mortality reported by the BRAC field offices overall, with 1 reported during labor and delivery (control group) and 2 occurring within 60 days postpartum (1 each in intervention and control groups). Cause and date of death for the postpartum events were not recorded; the recorded cause of the intrapartum death was PPH.

### Comparison of participant report and provider records

Overall, there were high levels of agreement between participant report and SK register sources for key event dates and specifics, like whether a delivery was vaginal or cesarean section, with Kappa statistics larger than 0.8 (Table 8). However, complications were recorded by SKs much less frequently than the level reflected by participant report; this was particularly true for antepartum and postpartum complications. Complications were recorded based on presence in the SK register or MNCH handbook, and study staff did not query presence of additional complications from the SK. Table 8 provides the full list of recorded complications (from the named documents) and stated complications (unprompted from participants during interview). Lower condition recording by providers appeared to be consistent for both non-life-threatening (e.g., low weight gain) and potentially life-threatening (e.g., severe bleeding) complications, though Kappa statistics were higher for conditions that could be objectively measured (e.g., blood pressure) or may have been perceived to be more serious (e.g., vaginal bleeding). For type of delivery, Kappa statistics were both above 0.90 (close to perfect agreement), and for postpartum weakness they were both below 0.1 (close to what would be expected by chance alone). For intrapartum eclampsia, there was only 1 person in the intervention group with this complication reported in the provider record, and none in the participant report.

**Table 8:** Comparison between community health provider record and participant report validation for key pregnancy event details and maternal complications among postpartum women from pooled intervention and control clusters in 4 districts of Bangladesh.

Variable	Source: Participant report (direct interview)	Source: SK register or MNCH handbook	Kappa statistic (95% CI)
Date of delivery			1.00 (0.99, 1.00)
Number of pregnancies (*n* = 1,096 matched records)			
Mean (SE)	2.3 (0.06)	2.3 (0.07)	0.95 (0.93, 0.96)
Median (IQR)	2	2	
Range	1–9	1–9	
Type of delivery (*n* = 1,076 matched records)			0.95 (0.92, 0.97)
Normal vaginal	806 (74.9%)	804 (74.7%)	
Cesarean	262 (24.3%)	267 (24.8%)	
Assisted vaginal	8 (0.7%)	5 (0.5%)	
Breastfeeding within 1 hour of delivery (*n* = 1,008 matched records)	(*n* = 1,014)	(*n* = 1,008)	
Yes	733 (72.7%)	930 (92.3%)	0.30 (0.24, 0.36)
Antepartum complications	(*n* = 1,100)	(*n* = 1,101)	(*n* = 1,098)
Any complication	812 (74.0%)	322 (29.3%)	0.19 (0.16, 0.23)
Severe nausea/vomiting	528 (48.1%)	160 (14.6%)	0.23 (0.19, 0.27)
High blood pressure	37 (3.4%)	23 (2.1%)	0.62 (0.48, 0.77)
Severe edema	182 (16.6%)	70 (6.4%)	0.47 (0.29, 0.54)
Low weight gain	17 (1.5%)	6 (0.5%)	0.25 (0.02, 0.49)
Gestational diabetes	12 (1.1%)	3 (0.3%)	0.40 (0.08, 0.71)
Fever/infection	194 (17.7%)	61 (5.6%)	0.33 (0.25, 0.40)
Vaginal bleeding	45 (4.1%)	21 (1.9%)	0.60 (0.45, 0.74)
Preterm labor	252 (23.0%)	40 (3.6%)	0.14 (0.08, 0.19)
Leakage of fluid/rupture of membranes	142 (12.9%)	32 (2.9%)	0.25 (0.17, 0.34)
Anemia	221 (20.1%)	36 (3.3%)	0.18 (0.12, 0.24)
Abdominal pain	49 (4.5%)	25 (2.3%)	0.08 (−0.02, 0.18)
Intrapartum complications	(*n* = 1,099)	(*n* = 1,070)	(*n* = 1,066)
Any complication	480 (45.0%)	307 (28.8%)	0.58 (0.53, 0.62)
Pre-eclampsia	29 (2.7%)	13 (1.2%)	0.56 (0.38, 0.74)
Eclampsia	9 (0.8%)	5 (0.5%)	0.57 (0.26, 0.88)
Heavy bleeding during labor	59 (5.5%)	21 (2.0%)	0.43 (0.30, 0.57)
Prolonged labor	257 (24.1%)	168 (15.8%)	0.53 (0.46, 0.59)
Obstructed labor	117 (11.0%)	39 (3.7%)	0.40 (0.31, 0.50)
Breech/non-cephalic presentation	40 (3.8%)	21 (2.0%)	0.61 (0.47, 0.76)
Fever/infection	34 (3.2%)	3 (0.3%)	0.10 (−0.03, 0.24)
Retained placenta	55 (5.2%)	27 (2.5%)	0.52 (0.39, 0.65)
Leakage of fluid/rupture of membranes	55 (5.2%)	37 (3.5%)	0.57 (0.45, 0.69)
Postpartum complications	(*n* = 1,099)	(*n* = 1,060)	(*n* = 1,057)
Any complication	421 (39.7%)	90 (8.5%)	0.20 (0.16, 0.25)
Eclampsia	21 (2.0%)	2 (0.2%)	0.17 (−0.04, 0.38)
Heavy bleeding	125 (11.8%) (of 1,100)	43 (4.1%) (of 1,060)	0.40 (0.31, 0.50)
Fever/infection	174 (16.5%)	20 (1.9%)	0.14 (0.07, 0.20)
Abdominal pain	214 (20.2%)	5 (0.5%)	0.03 (−0.001, 0.06)
Weakness	308 (29.1%)	14 (1.3%)	0.05 (0.03, 0.09)

Data given as number (percent), unless otherwise indicated.

MNCH, maternal, newborn, and child health; SK, Shasthya Kormi.

## Discussion

Maternal mortality has plateaued in Bangladesh over the last 6 years, and this may reflect maximal impact of current interventions to increase ANC and facility-based delivery [27], with further interventions needed to create momentum around reducing maternal morbidity and mortality. The key findings from this exploratory assessment of the impact of a maternal nutrition intervention on maternal complications are lower rates of reported antepartum, intrapartum, and postpartum complications among women from intervention areas, which emerged as a pattern for antepartum and intrapartum complications and achieved statistical significance for postpartum complications. Specific and potentially life-threatening complications of intrapartum retained placenta, postpartum infection, and PPH were also significantly lower among women from intervention areas in crude (for retained placenta and infection) and adjusted analyses. We note that supplement use and dietary diversity scores were higher among women from intervention areas, consistent with findings from the parent study [28]. However, complications and ensuing care and dietary inputs are based on participant report, and there were low rates of agreement between SK records and participant reports for many conditions. As such, we relied solely on participant report for all complication measures for uniformity and to guard against SK underreporting. However, some serious conditions that differed significantly by intervention exposure, like reported postpartum fever or heavy postpartum bleeding, had low agreement between sources, and the differences by study group could reflect differing perceptions of participant and care provider rather than actual event occurrence. The intervention, which included more frequent provider visits, may have improved women’s ability to discern true complications, leading to a lower complication reporting rate, rather than lower incidence of complications. This may be particularly true for nutrition-related complications or conditions, including malnutrition. This possibility is strengthened based on knowledge change surrounding maternal complications that might be prevented with supplement use, including anemia and hemorrhage with IFA supplements and pre-eclampsia/eclampsia with calcium supplements [33]. Conversely, heightened awareness of complications may have resulted in higher recall of complications among women in intervention areas. These findings related to a reported outcome measures indicate need for a more rigorous evaluation.

Reported antepartum complications were wide-ranging, and the perceived severity varied widely between the participants’ perspective and that of the SK, based on the low Kappa statistics for most reported conditions. We believe some of this disparity may be due to provider perceptions surrounding conditions severe enough to be recorded in written records compared to symptoms interpreted as complications by the patient. We tried to be as inclusive as possible in listing complications and note that the symptoms of the conditions reported were notable enough to be recalled in the postpartum period, but that SK visits occurring monthly or less may have reduced opportunities for both reporting and recording. The conditions with higher Kappa scores were those most likely to contribute to hospitalizations or to need further care, such as anemia, reflecting provider attention to potentially serious conditions. Anemia and malnutrition were reported more frequently by women in control areas, which is concerning since women from these areas also reported lower postpartum dietary diversity scores and antepartum supplement use. As the study’s nutrition intervention is taken to scale, we recommend adding objective measures to validate maternal nutrition status, such as MUAC and hematocrit point-of-care testing.

Reported intrapartum complications were lower among intervention participants, particularly retained placenta and fever/infection, and this association became significant in analysis adjusted for prior pregnancy complications. We believe these specific conditions may have a biological basis for being lower among women exposed to a nutrition intervention. First, immune function towards all pathogens is optimized by adequate nutrition and micronutrient intake and tissue oxygenation [34,35]. Next, abnormal placental vascularization and function have been linked to micronutrient deficiencies in animal and in vitro models and in 1 study in Nepal [15,34,35]. As supplement use and dietary diversity were by participant report, we recommend micronutrient level assessment in future evaluation of interventions with multivitamin and iron supplements, reflecting current recommendations [36]. The cesarean section rate was greater than 20% for both control and intervention participants, far higher than expected in this context, and also consistent with that reported in the parent study. Cesarean section rates did not reflect complications and raise the concern that cesarean sections may be performed for non-medical indications, with resultant increased morbidity, as noted in other contexts [37]. However, it is possible that doctors did not fully explain the complication precipitating the decision to proceed to cesarean or that the woman may have forgotten the indication or chose not to disclose the condition. We also note that this cohort had relatively low rates of facility-based delivery, potentially increasing both the number and severity of intrapartum complications, and suggesting the need for intensified messaging for skilled birth attendance within the community-based MNCH program.

Postpartum complications were lower among women from intervention areas, even when adjusted by antepartum and intrapartum complications, facility-based delivery, and prior pregnancy complications. Within postpartum complications, bleeding following delivery and weakness, possibly suggestive of PPH, were significantly higher among participants from control areas, even when analysis was controlled for ante- and intrapartum complications and delivery in a facility. PPH is the leading cause of maternal mortality and morbidity both globally and in Bangladesh, and prior studies suggest that antenatal anemia predisposes women to obstetric hemorrhage [3,4,19,27]. While the difference in all-cause antepartum complications between intervention and control groups did not achieve statistical significance, malnutrition by participant report and anemia by SK report were much more common among women in the control areas and may have predisposed this group to postpartum bleeding complications. This report should be treated with caution as the parent study did not detect any significant differences in the proportion of underweight women by BMI measures between intervention and control areas at either baseline or endline surveys [28].

Agreement between participant report and SK-recorded data was substantial (Kappa > 0.61) for key obstetric events, such as date and route of delivery. We acknowledge the high level of data recording accuracy demonstrated by BRAC community-based workers, who also were present at many of the deliveries and conducted immediate PNC. However, there were considerably lower levels of agreement surrounding maternal complication reporting, as SKs reported much lower levels of complications, potentially reflecting provider perceptions regarding normal symptoms of pregnancy (e.g., nausea) or the perception that vague complaints in the absence of examination findings (e.g., back pain) are not true complications and thus do not merit comment in a written record. Some complications (e.g., prolonged labor) may only have been recognized, been diagnosed, or occurred at a health facility and thus may not have been recorded in the SK register or women’s record as the SK was not present or the situation was an emergency precluding written documentation. Further investigation is recommended to better understand SK perceptions surrounding complications and the degree to which patients are queried by SKs about health encounters with other providers, in an effort to improve recording accuracy for serious conditions. There are some key data missing from many records, and in the control areas, some data were not part of the SK record and thus not captured, such as maternal weight and dietary recall in the last 24 hours. Greater oversight and periodic retraining/supportive supervision are needed as well as attention to whether there is too great a reporting burden on SKs. We did not specifically query whether completion of the MNCH handbook and the SK register was viewed as redundant or burdensome among SKs, but we often found that SKs chose not to take the register with them on their field visits, completing data entry later at home, with the attendant risk of mistakes or incomplete entry. At the time of developing and collecting data for this assessment, a mobile-phone-based data entry program was being developed and pilot-tested to eventually replace the written register. No data for this assessment was collected through this mobile-phone-based system, but adoption of this system may improve information collection in real time. The new data collection system should be similarly audited against patient report, as done here, to determine fidelity and completeness of SK reporting, and compared against the data from this assessment to determine whether the mobile phone system is an improvement over the paper-based system.

Maternal nutrition indicator measurement was conducted with 24-hour dietary recall and monthly supplement intake, both based on participant report and possibly on SK count of IFA and calcium pills in intervention areas. While dietary recall measures are used in national surveys and have been validated in Bangladesh [38], we recommend caution in comparing the dietary diversity measures in this assessment: Women in the intervention areas had information elicited by trained SKs and had experience in reporting these measures throughout their pregnancies, as the data were taken from the SK register. The higher mean dietary diversity score and higher mean supplement intake among women from intervention areas may stem from the intensive nutrition counseling and supplement provision. However, socially desirable response among intervention participants or having highly trained SKs elicit the information, compared to study staff who were not trained health providers, may also have produced this difference. Study staff were trained extensively, with satisfactory practical demonstration of dietary diversity questionnaire administration on several mock “patients,” before they were permitted to collect data in the field. However, it is possible that the greater familiarity of the SKs and the intervention participants with the instrument and process and the specialized training of the SK, with understanding to probe for forgotten foods, like milk in tea, may have contributed to the dietary diversity score difference between the intervention and control groups. Differences noted in dietary diversity score among intervention participants, who had food recall measured by SKs during visits, were pronounced between first and last ANC visits, but the PNC visit dietary diversity scores approached those of the first ANC visit. For the intervention group, this lability in scores based on SK register records may reflect the impact of seasonality, but could also reflect patient “learning” of the socially desirable responses to characterize optimal food intake through the pregnancy that dissipates in the longer between-visit interval after delivery. Alternately, nutrition habits that resulted in lower diversity scores may have been resumed after delivery if the need for better diet was solely associated with pregnancy for women and/or key influencers in the household. There are also prevalent traditional practices and taboos associated with certain foods for their impact on breastmilk or the general well-being of the mother or infant, and these foods are thus removed from the diet postpartum. Unfortunately, lack of an objective measure of maternal nutrition status prevents informed speculation about the cause; future assessments should include maternal postpartum weight and MUAC measures.

### Limitations

These results should be interpreted while considering several key limitations. As already noted, outcome measures were based on participant report, which may introduce both recall and reporting bias or simply reflect differential levels of knowledge about complications rather than the occurrence of the complications themselves. As less than half of deliveries occurred in health facilities, it was not possible to check the presence and severity of reported complications against facility records or with other means of medical verification. The complication rate recorded by SKs was lower than that from participant report and may be a better measure of complications as conditions were likely only recorded if they resulted in referral to a facility or some other treatment action, assuming SKs reliably recorded these data. Our field staff carefully queried reported events and then gently probed whether the participant believed the event was a complication, but during training, field staff were also counseled to establish rapport with participants and may not have probed intensively due to concerns about projected mistrust.

Next, the 20 SKs from whose catchment areas participants were recruited were not randomly selected. SKs were chosen based on advanced training and quality performance within the BRAC MNCH program, and no data were collected on the chosen SKs to determine whether they differed significantly from SKs included within the parent study overall. Additionally, because SK-specific data were not collected, the cluster stage within the hierarchical models was constrained to the sub-district level, potentially obscuring differences attributable to care provision by specific SKs. Due to time and resource constraints and the relatively brief window of eligibility in the postpartum period, mapping was not conducted, and a non-probabilistic sample of all eligible postpartum women in the SK catchment area was recruited. However, we believe that using the same randomized clusters from the parent trial affords a degree of comparability, corroborated by the similarities between most intervention and control group demographic characteristics within this sample and with the parent study sample.

There were also varying amounts of data missing within SK reporting forms. During the study preparation phase, we sought to reduce respondent and field staff burden to the degree possible by minimizing duplication of measures between questionnaire and data abstraction forms. However, abstraction forms were often missing large amounts of information, including critical measures, such as hospitalizations. Thus, some measures must be interpreted with caution due to the amount of missing data.

It is possible that some women eligible for entry to this study were not accessed due to death or leaving the area. Deaths are rigorously monitored through BRAC field reporting, and we believe additional maternal mortality cases, though possible, are unlikely. Women experiencing pregnancy loss or fetal demise were also not included in the study, potentially reducing representation of cases where these events resulted from a maternal complication. Inaccessibility of participants due to leaving the area after delivery is possible, as women traditionally return to their parent’s home for delivery and the early postpartum period. Due to this cultural norm, we extended the eligibility period to 60 days postpartum, as the consensus among our research team and BRAC field staff was that women return to their husband’s family between 40 and 49 days postpartum.

Last, reported dietary intake and supplement use are subject to recall bias and may also be influenced by socially desirable response in intervention areas. Because there were no objective maternal nutrition measures (such as MUAC or body mass index) equally applied to participants from both intervention and control areas, we cannot definitively comment on whether the higher supplement intake and dietary diversity reported by intervention area participants are valid, particularly as both of these outcomes were impacted by social desirability in reporting [28]. Further, we acknowledge that dietary diversity scores vary by season due to the availability of different foods during the year. We did not record the dates at which antenatal and postnatal dietary diversity scores were abstracted from the SK register for intervention group participants, and the antenatal scores, taken at first and last ANC visits, were most likely from different seasons, assuming that ANC was initiated before 20 weeks gestation and that the last visit was at 38–39 weeks gestation. The PNC visit would have been within a few weeks of data collection for this study as the window of eligibility for this study was only 60 days postpartum, minimizing seasonal variation between dietary diversity scores for control group participants (collected at time of interview) and the PNC visit dietary diversity scores for intervention group participants. However, we did not collect dates for the PNC visit dietary diversity scores and thus cannot say with certainty that this information was collected during the same season as the scores taken during the study interviews for control participants.

### Conclusions

Reported postpartum complications, particularly postpartum bleeding, were significantly more prevalent among women in control areas in this exploratory analysis. This finding may be attributable to intervention exposure via direct impact on actual complications, intervention exposure via indirect impact on maternal ability to define a complication, or other causes not detected by this assessment. We are confident that the intervention did not impact care-seeking in the antepartum or intrapartum period based on similar skilled/facility-based care statistics for the intervention and control groups. Community-based care providers recorded key obstetric events accurately and at high rates, but greater understanding of defining maternal complications and ensuring provision of appropriate care is needed and should be pursued in formative studies prior to the next large-scale intervention. While dietary diversity and supplement intake measures were significantly higher among women from intervention areas, lack of objective nutrition measures and non-random selection of participants reduce our ability to measure the impact of the parent intervention on maternal outcomes. We intend the results to guide planning and communications around large-scale intensive nutrition counseling and micronutrient supplementation within community-based MNCH programs, and recommend that future maternal nutrition interventions incorporate a randomized prospective cohort of pregnant women to assess causal differences in maternal morbidity, and use objective, sufficiently rigorous measures for nutrition factors.

## Supporting information

S1 Data Analysis Plan (DOCX)

S1 Fig Length of time in labor among postpartum women in intervention and control areas in 4 districts of Bangladesh. (TIF)

S2 Fig Maternal iron/folic acid (IFA) and calcium (Ca) supplement intake recorded by Shasthya Kormi at first antenatal care visit among women in nutrition intervention compared to women in control areas in 4 districts of Bangladesh. (TIF)

S3 Fig Maternal iron/folic acid (IFA) and calcium (Ca) supplement intake recorded by Shasthya Kormi at last antenatal care visit among women in nutrition intervention and control areas in 4 districts of Bangladesh. (TIF)

S4 Fig Maternal iron/folic acid (IFA) and calcium (Ca) supplement intake recorded by Shathya Kormi at last recorded postnatal care visit among women in intervention and control areas in 4 districts of Bangladesh. (TIF)

S1 STROBE Checklist STROBE checklist of items that should be included in reports of cross-sectional studies. (DOC)

S1 Study Instruments SK data abstraction form and recently delivered women interview form. (PDF)

S1 Table Hierarchical logistic regression models assessing association of reported antepartum complications with exposure to a maternal nutrition intervention in 4 districts of Bangladesh. (DOCX)

S2 Table Hierarchical logistic regression models assessing association of reported overall intrapartum complications with exposure to a maternal nutrition intervention in 4 districts of Bangladesh. (DOCX)

S3 Table Hierarchical logistic regression model for differences in reported retained placenta between women exposed to a maternal nutrition intervention and those in control areas in 4 districts of Bangladesh. (DOCX)

S4 Table Hierarchical logistic regression model for differences in reported intrapartum fever or infection between women exposed to a maternal nutrition intervention and those in control areas in 4 districts of Bangladesh. (DOCX)

S5 Table Hierarchical logistic regression model assessing association of overall postpartum complications with exposure to a maternal nutrition intervention in 4 districts of Bangladesh. (DOCX)

S6 Table Hierarchical logistic regression model assessing association of reported postpartum hemorrhage with exposure to a maternal nutrition intervention in 4 districts of Bangladesh. (DOCX)

## Abbreviations

ANC: antenatal care
AOR: adjusted odds ratio
IFA: iron/folic acid
MMR: maternal mortality ratio
MNCH: maternal, newborn, and child health
MUAC: mid-upper arm circumference
OR: odds ratio
PNC: postnatal care
PPH: postpartum hemorrhage
RA: research assistant
SK: Shasthya Kormi
SS: Shasthya Shebika
