# Supplementary material for: Maternal nutrition intervention and maternal complications in 4 districts of Bangladesh: A nested cross-sectional study
Source: PLoS Med. 2019 Oct 4;16(10):e1002927. doi: 10.1371/journal.pmed.1002927 (PMC6777761; doi:10.1371/journal.pmed.1002927)

**Cross-sectional assessment of impact of nutrition intervention on maternal health outcomes among postpartum women in Mymensingh, Rangpur, Lalmonirhat, and Kurirgram districts in Bangladesh**

**DATA ANALYSIS PLAN**

**Last revised**: April 21, 2017

**Study Summary**

Cross-sectional assessment of impact of nutrition intervention on maternal health outcomes among postpartum women in Mymensingh, Rangpur, Lalmonirhat, and Kurirgram districts in Bangladesh.

*Design*

Cross-sectional quantitative comparison nested within existing cluster-randomized nutrition survey.

*Population*

Approximately 1000 post-partum women between 42 and 60 days post-delivery

*Study Duration*

Approximately 9 months in total, 7 months for instrument testing and data collection, 2 months for analysis and results synthesis

*Study Sites*

Twenty sub districts/upazilas in Rangpur, Lalmonirhat, Kurirgram, and Mymensingh districts, randomly selected by the larger evaluation.

**Analysis background**

We start by noting the sample for this study has been purposively selected from the intervention and control groups in each of the four districts. Specifically,

- the 20 upazilas in this study have been purposively selected from both intervention and control upazilas, and
- the SKs were purposively selected based on personnel who have received additional training in record-keeping and advanced patient monitoring, including skills such as hemoglobin measurement.

This study is embedded within a larger intervention study. That larger intervention study is a CRT with women nested within SKs, SKs nested within Upazilas, and Upazilas nested within districts. Randomization into the treatment and control conditions was at the Upazila level.

For this study, a total of 40 SKs (2 of approximately 50 per Upazila) were purposively selected from both the intervention and control Upazilas across the 4 districts. Of the SKs selected for this study, 20 were selected from 10 Upazilas in the intervention group and 20 from 10 Upazilas in the control group. Thus, we have women within SKs, SKs within Upazilas, Upazilas within districts, and districts within regional areas.

**Primary Objective, Outcome Variables and Analysis**

Primary Objective: To compare rates of reported peripartum and postpartum maternal morbidities between women residing in areas exposed to the intervention focusing on dietary content and micronutrient consumption and those residing in control areas.

We propose comparing the women in the treatment and control groups grouped by stage using the following endpoints and outcome variables:

*Stage 1: Antepartum morbidities:*

1. Percentage of women who had any complication during her pregnancy before going into labor (Q400)
2. Percentage of women who had (Q401a through Q401l):
   1. Anemia
   2. Severe vomiting
   3. Severe edema
   4. High blood pressure
   5. Vaginal bleeding
   6. Pre eclampsia
   7. Diabetes
   8. High fever
   9. Preterm labor
   10. Low weight gain
   11. Leakage of fluid/preterm rupture of membranes
   12. Back pain
3. Percentage of women who went to the hospital due to complications (Q402)
4. Percentage of women who received a blood transfusion at the hospital due to these complications (Q403b)

*Stage 2: Peripartum morbidities:*

1. Percentage of women who had any complications during labor and delivery (Q406)
2. Percentage of women who had (Q407a through Q407j):
3. Pre-eclampsia
4. Eclampsia/convulsions
5. Heavy bleeding during labor
6. Heavy bleeding after delivery
7. Retained placenta
8. Prolonged labor
9. Obstructed labor
10. Fever/infections
11. Breech/non-cephalic presentation
12. Fever/infection in labor
13. Percentage of women who went to the hospital due to these complications (Q408)
14. Percentage of women who received a blood transfusion at the hospital due to these complications (Q409b)

*Stage 3: Post-partum morbidities:*

1. Percentage of women who had any complications after the delivery (Q410)
2. Percentage of women who had (Q411):
3. Eclampsia/convulsions
4. Heavy bleeding after delivery
5. Fever
6. Severe lower abdominal pain
7. Infection
8. Severe weakness
9. Percentage of women who went to the hospital due to these complications (Q412)
10. Percentage of women who received a blood transfusion at the hospital due to these complications (Q413b)

## Descriptive analysis

We will present the results of these comparisons using a separate table for each stage (see the Appendix for a table shell example). We will assess the difference in the percentages of the treatment and control groups after taking into account the clustering of women within Upazilas. For this we plan to use the SAS SURVEYFREQ procedure with the CLUSTER statement. For each comparison, we will report a Chi-square statistic. For each stage, we will consider whether there is a difference in the percentages of women who had any complications to be the main comparison. The main primary endpoints are thus 1, 5 and 9 above; we will be using a 0.05 level of significance for these comparisons. For each stage, if there is a statistically significant difference in the main comparison, we will then assess the specific complications and whether the women went to the hospital and received a blood transfusion due to those complications. For these we will use a 0.01 level of significance to account for the multiple hypotheses testing. We will not report Chi-square statistics for sparse cross-tabulations (i.e., less than 5 expected counts in any cell).

## Regression analysis

To further study the association between any complication and the intervention, we will fit a series of hierarchical generalized linear regression models (HGLMs), weighted for clustering at the upazila level. Specifically, for each stage, we will fit a HGLM on any complication (coded 1=Yes, 0=No), controlling for selected variables reflecting the respondent’s household socio-economic (SES; Q100 - Q133b), obstetric history / medical history (OMH; Q301 – Q305), and other variables from the abstraction form (e.g., age). The model will account for the clustering of women within Upazilas and for the assignment into either the treatment or control group at the Upazila level. We will control for the districts using fixed effects.

For the household SES control variables, we propose conducting an exploratory factor analysis (EFA) to combine into a smaller, more manageable set of variables to be included in the model. For this EFA, we would take into consideration that some of those variables are continuous, some are ordinal, and some are unordered categorical. For the unordered categorical, we would create indicators for each response category. We will account for missing data and for non-viable response options (e.g., a response category of “Other” in a categorical variable) by fitting the EFA on the variance-covariance matrix.

For the OMH control variables, we plan to initially use those derived from items Q301-Q305 in the questionnaire. Specifically:

- Number of prior miscarriages (301)
- Number of prior preterm deliveries (Q302)
- Number of prior term deliveries (Q303)
- Number of living children
- Indicators for each of the following:
  - Diabetes
  - High blood pressure
  - Malnutrition when not pregnant
  - Under-weight
  - Asthma
  - Tuberculosis
  - Anemia, pallor
  - Heart condition/rheumatic heart disease
  - Kidney disease

Final decisions regarding the number of factors and/or variables to be included in the model can be revised slightly based on substantive knowledge and data characteristics, including amount of missing data, sparseness, variability, and multiple correlation amongst the selected variables.

For the stage 2 model (Morbidities at delivery and postpartum) we will also include an indicator for any complications during stage 1 (peri-partum morbidities), and for the stage 3 model (morbidities post partum) we will include indicators for any complications during stages 1 and 2. We suggest reporting the results of these models in separate tables, including in each case selected model summary and diagnostic statistics.

**Secondary Objective, Outcome Variables and Analysis**

The secondary objectives of the study are:

- To assess the current accuracy and level of detail of maternal and child health data collected within surveillance forms by BRAC community health workers.
- To determine feasibility of measuring nutrition indicators within maternal health programs.
- To explore the value added of various innovative programs (husband’s forum, community events, etc.)

The endpoints proposed for this are:

- Rate at which peripartum complications and other key indicators, such as birth weight and breastfeeding status, are recorded and specified in existing Shasthya Kormi (SK) records (red book, register) as compared to those elicited in participant interview.
- Rate of correctly completed daily food intake and supplement use captured in SK forms and correlation to that reported in participant interview (intervention areas).
- Rates of reported awareness, attendance, and behaviors attributed to community-based maternal/child nutrition programs (intervention areas) (Q213, Q214, Q800, Q801, Q802)

More specifically, we will compare the response rates for the pairs of variables as listed in the table below. We will for each pair compute a Cohen’s Kappa statistic.

| Variable | Number in  questionnaire | Number in  abstract |
| --- | --- | --- |
| Prior pregnancies | 300 | 101 |
| Delivery date | 309 | 300 |
| Complications during pregnancy |  |  |
| Any complications | 400 | 120.1 |
| Severe nausea/vomiting | 401b | 120.2 |
| High blood pressure | 401d | 120.3 |
| Severe edema | 401c | 120.4 |
| Low weight gain | 401j | 120.5 |
| Gestational diabetes | 401g | 120.6 |
| Fever/infections | 401h | 120.7 |
| Vaginal bleeding | 401e | 120.8 |
| Preterm labor | 401i | 120.9 |
| Leakage of fluid/preterm rupture of membranes | 401k | 120.10 |
| Anemia | 401a | 120.11 |
| Complications during delivery |  |  |
| Any complications | 406 | 306 |
| Pre-eclampsia | 407a | 307.1 |
| Eclampsia/convulsions | 407b | 307.2 |
| Heavy bleeding during labor | 407c | 307.3 |
| Heavy bleeding after delivery | 407d | 307.4 |
| Prolonged labor/insufficient cervical dilation | 407f | 307.5 |
| Prolonged labor, pushing more than 2 hours | 407f | 307.6 |
| Obstructed labor | 407g | 307.7 |
| Breech/non-cephalic | 407i | 307.8 |
| Fever/infection in labor | 407h, 407j | 307.9 |
| Retained placenta | 407e | 307.11 |
| Post-partum complications |  |  |
| Any complications | 410 | 319 |
| Eclampsia | 411a | 320.2 |
| Heavy bleeding | 411b | 320.3 |
| Fever | 411c | 320.4 |
| How long after birth put baby to breastfed | 500 | 312 |
| Type of delivery | 310 | 303 |

**Appendix 1**: Example table for reporting descriptive statistics per stage. Example is for stage 1.


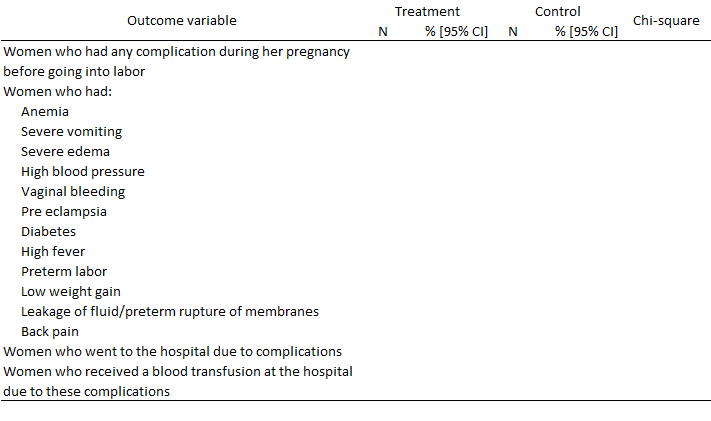

Supplement: S1 Data Analysis Plan — (DOCX) [file pmed.1002927.s001.docx]
