# Supplementary figures and images for: Maternal nutrition intervention and maternal complications in 4 districts of Bangladesh: A nested cross-sectional study
Source: PLoS Med. 2019 Oct 4;16(10):e1002927. doi: 10.1371/journal.pmed.1002927 (PMC6777761; doi:10.1371/journal.pmed.1002927)

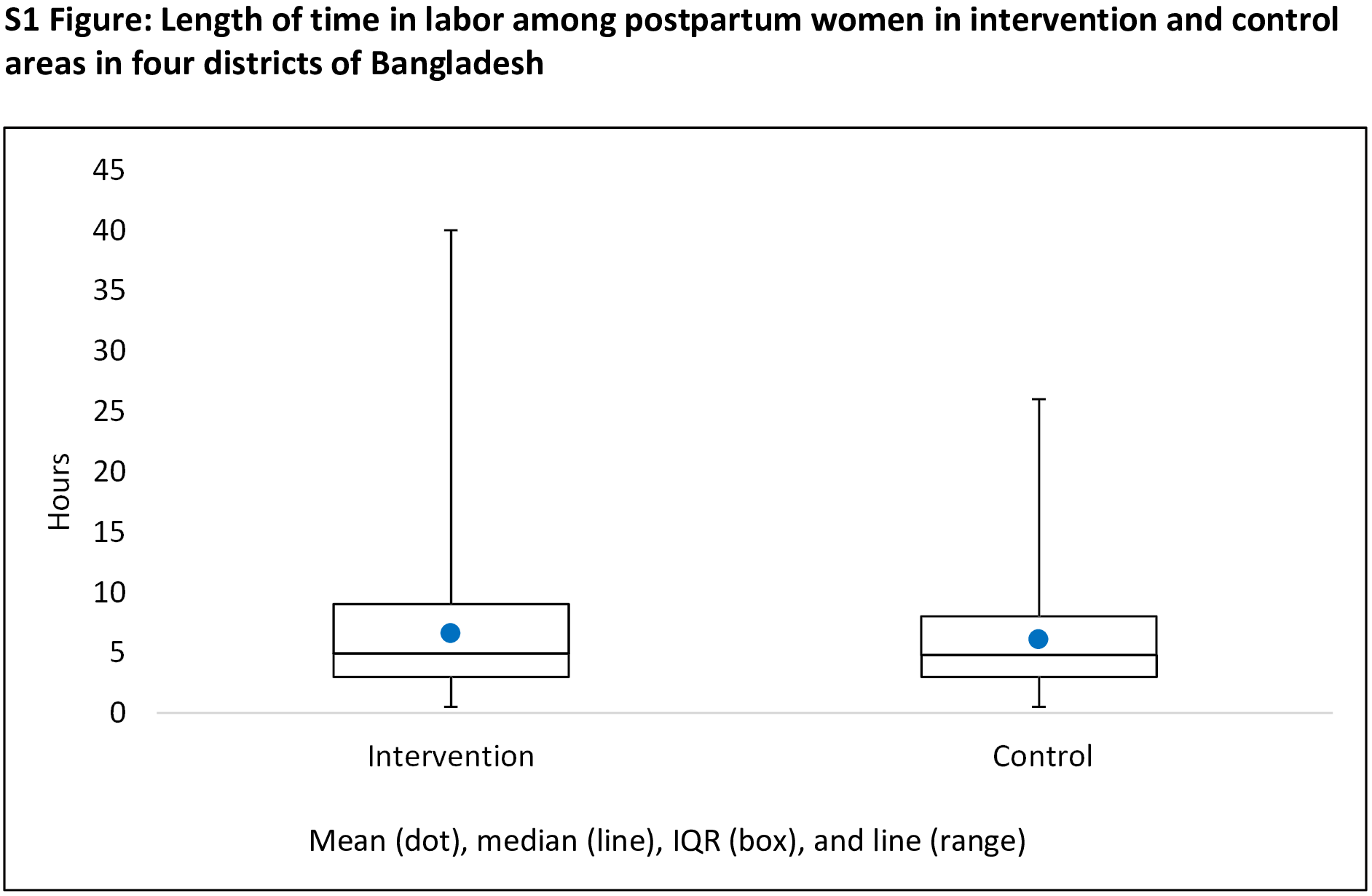

Supplement: S1 Fig — (TIF) [file pmed.1002927.s002.tif]

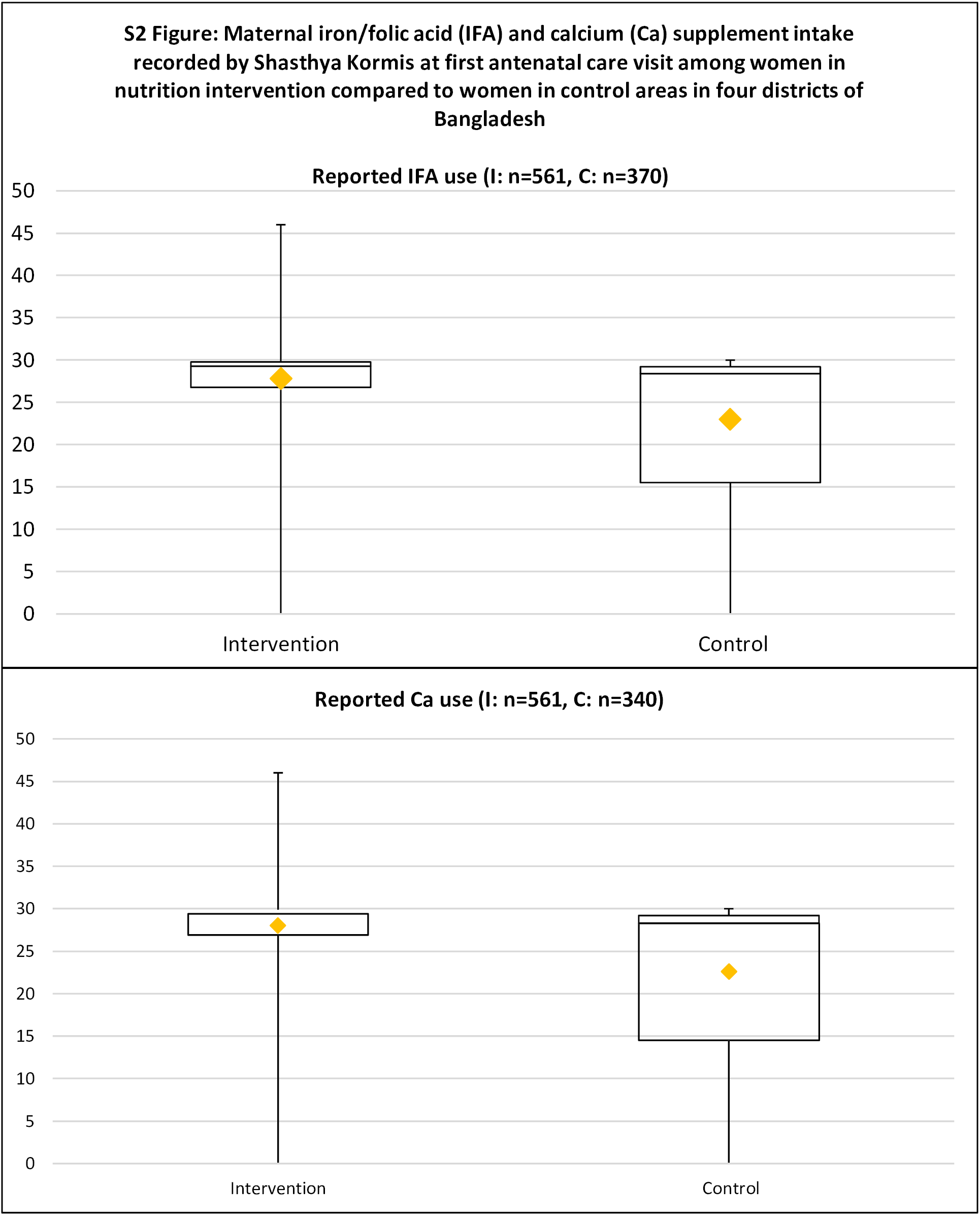

Supplement: S2 Fig — (TIF) [file pmed.1002927.s003.tif]

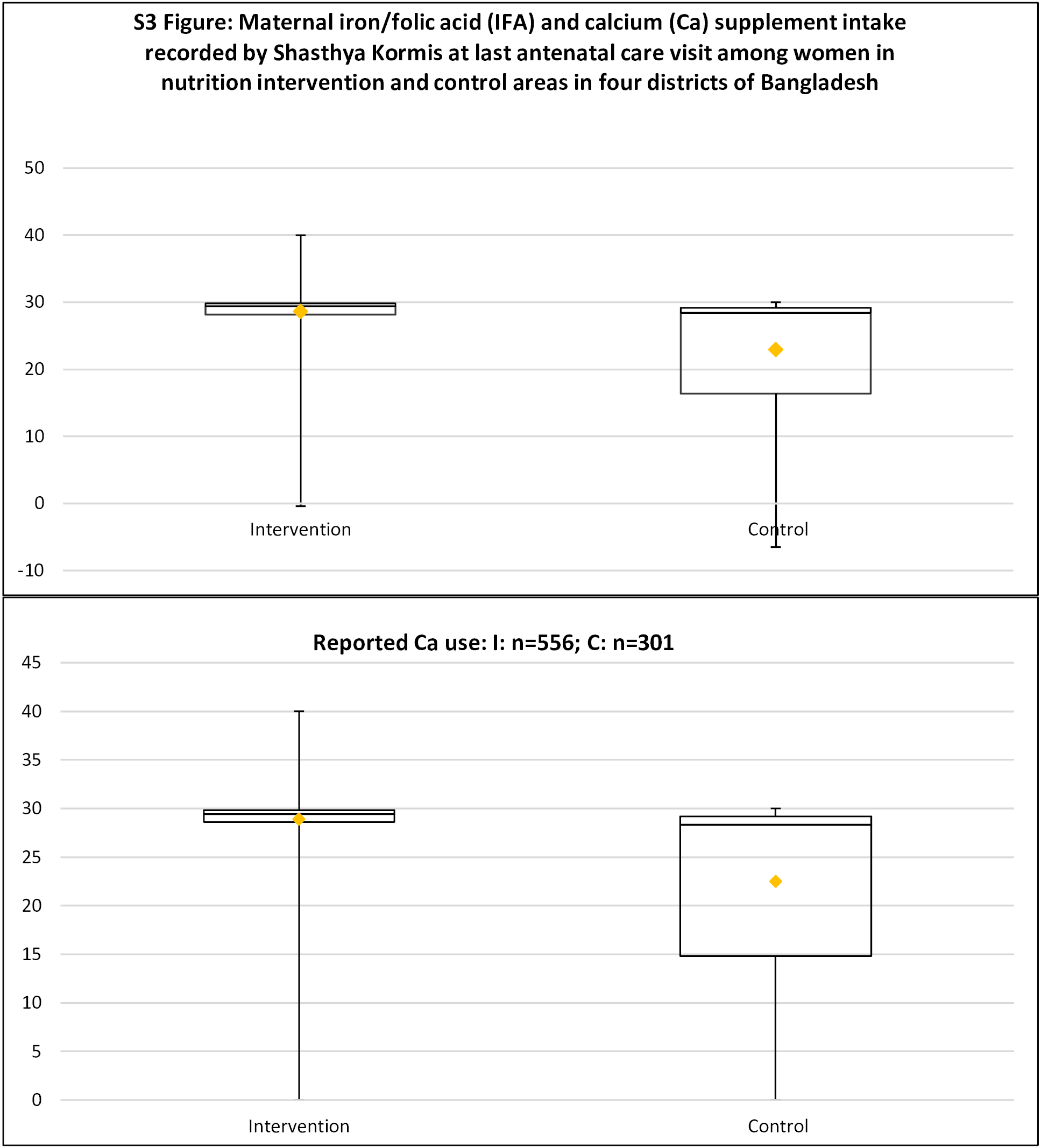

Supplement: S3 Fig — (TIF) [file pmed.1002927.s004.tif]

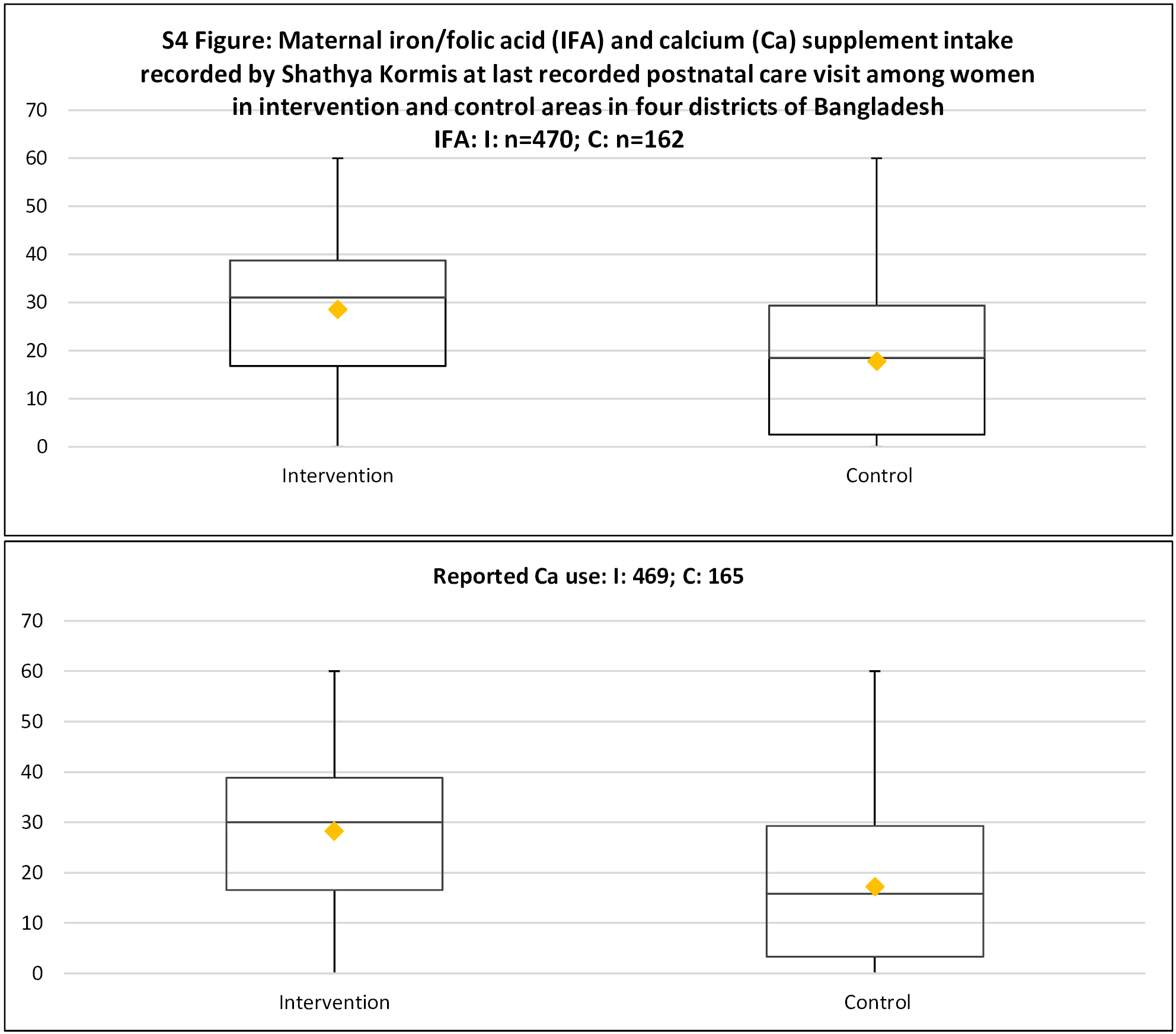

Supplement: S4 Fig — (TIF) [file pmed.1002927.s005.tif]
