# Supplementary material for: Maternal nutrition intervention and maternal complications in 4 districts of Bangladesh: A nested cross-sectional study
Source: PLoS Med. 2019 Oct 4;16(10):e1002927. doi: 10.1371/journal.pmed.1002927 (PMC6777761; doi:10.1371/journal.pmed.1002927)
Supplement: S1 STROBE Checklist — (DOC) [file pmed.1002927.s006.doc]

STROBE Statement—Checklist of items that should be included in reports of ***cross-sectional studies***

|  | Item No | Recommendation |
| --- | --- | --- |
| **Title and abstract** | 1 | (*a*) Indicate the study’s design with a commonly used term in the title or the abstract:  ***Design present in title and abstract as nested cross-sectional study.*** |
|  |  | (*b*) Provide in the abstract an informative and balanced summary of what was done and what was found: ***Abstract and Author Summary.*** |
| Introduction | | |
| Background/rationale | 2 | Explain the scientific background and rationale for the investigation being reported: ***Introduction, paragraphs 2-5.*** |
| Objectives | 3 | State specific objectives, including any prespecified hypotheses: ***Purpose statement: Introduction, paragraph 5.*** |
| Methods | | |
| Study design | 4 | Present key elements of study design early in the paper: ***Paragraph 1 of Study Design & Participants sub-section, Methods section.*** |
| Setting | 5 | Describe the setting, locations, and relevant dates, including periods of recruitment, exposure, follow-up, and data collection: ***Intervention exposure in Intervention sub-section; &*** s***etting & data collection period in Study Design & Participants sub-section of Methods section.*** |
| Participants | 6 | (*a*) Give the eligibility criteria, and the sources and methods of selection of participants. ***Eligibility criteria in Study Design & Participants sub-section; participant sources and selection described in Data Collection Procedures sub-section, Methods section.*** |
| Variables | 7 | Clearly define all outcomes, exposures, predictors, potential confounders, and effect modifiers. Give diagnostic criteria, if applicable. ***All paragraphs of*** ***Measures sub-section, Methods section.*** |
| Data sources/ measurement | 8* | For each variable of interest, give sources of data and details of methods of assessment (measurement). Describe comparability of assessment methods if there is more than one group. ***All paragraphs of Measures sub-section and pictorially displayed in Figure 1, Methods section.*** |
| Bias | 9 | Describe any efforts to address potential sources of bias. ***Paragraphs 3-6 of*** ***Measures sub-section, Methods section.*** |
| Study size | 10 | Explain how the study size was arrived at: ***Paragraph 1, Analysis sub-section, Methods section.*** |
| Quantitative variables | 11 | Explain how quantitative variables were handled in the analyses. If applicable, describe which groupings were chosen and why. ***Paragraph 3 of Analysis sub-section, Methods section.*** |
| Statistical methods | 12 | (*a*) Describe all statistical methods, including those used to control for confounding: ***Paragraph 3,*** ***Analysis sub-section, Methods section.*** |
|  |  | (*b*) Describe any methods used to examine subgroups and interactions: ***No sub-group analysis was conducted in this study and no interactions were detected.*** |
|  |  | (*c*) Explain how missing data were addressed: ***Paragraph 2, Analysis sub-section, Methods section.*** |
|  |  | (*d*) If applicable, describe analytical methods taking account of sampling strategy: ***Paragraphs 2 & 3, Analysis sub-section, Methods section..*** |
|  |  | (*e*) Describe any sensitivity analyses: ***Not done.*** |
| Results | | |
| Participants | 13* | (a) Report numbers of individuals at each stage of study—eg numbers potentially eligible, examined for eligibility, confirmed eligible, included in the study, completing follow-up, and analysed: ***We did not map participants and have stated this is a non-probabilistic sample. No participants declined entry and the number included in each segment of analysis are stated in the tables.*** |
|  |  | (b) Give reasons for non-participation at each stage: ***N/A*** |
|  |  | (c) Consider use of a flow diagram: ***We deferred this due to single time point encounter and no eligible women declining enrolment.*** |
| Descriptive data | 14* | (a) Give characteristics of study participants (eg demographic, clinical, social) and information on exposures and potential confounders: ***Tables 1 and 2, Results section.*** |
|  |  | (b) Indicate number of participants with missing data for each variable of interest: ***Tables 1 – 8, Results section.*** |
| Outcome data | 15* | Report numbers of outcome events or summary measures: ***Tables 5 – 7 & Supplementary Tables 1-6, Results section.*** |
| Main results | 16 | (*a*) Give unadjusted estimates and, if applicable, confounder-adjusted estimates and their precision (eg, 95% confidence interval). Make clear which confounders were adjusted for and why they were included: ***Supplemental tables 1-6.*** |
|  |  | (*b*) Report category boundaries when continuous variables were categorized: ***Tables 1 – 8, Supplemental Tables 1 – 6, Results section.*** |
|  |  | (*c*) If relevant, consider translating estimates of relative risk into absolute risk for a meaningful time period: ***N/A*** |
| Other analyses | 17 | Report other analyses done—eg analyses of subgroups and interactions, and sensitivity analyses. ***Secondary analyses assessing dietary diversity score and differences between participant report and data recorded by SKs are reported in Tables 4 and 8, Results section.*** |
| Discussion | | |
| Key results | 18 | Summarise key results with reference to study objectives: ***Paragraph 1, Discussion section.*** |
| Limitations | 19 | Discuss limitations of the study, taking into account sources of potential bias or imprecision. Discuss both direction and magnitude of any potential bias: ***Limitations sub-section, Discussion section.*** |
| Interpretation | 20 | Give a cautious overall interpretation of results considering objectives, limitations, multiplicity of analyses, results from similar studies, and other relevant evidence: ***Paragraph 1 of Discussion section and Conclusions sub-section of Discussion section; Author summary.*** |
| Generalisability | 21 | Discuss the generalisability (external validity) of the study results: ***Paragraph 1 of Study Design & Participants sub-section, Methods section, &*** ***Limitations sub-section, Discussion section.*** |
| Other information | | |
| Funding | 22 | Give the source of funding and the role of the funders for the present study and, if applicable, for the original study on which the present article is based. ***Funding source and role of donor provided with meta-data.*** |

*Give information separately for exposed and unexposed groups.

**Note:** An Explanation and Elaboration article discusses each checklist item and gives methodological background and published examples of transparent reporting. The STROBE checklist is best used in conjunction with this article (freely available on the Web sites of PLoS Medicine at http://www.plosmedicine.org/, Annals of Internal Medicine at http://www.annals.org/, and Epidemiology at http://www.epidem.com/). Information on the STROBE Initiative is available at www.strobe-statement.org.
