# Supplementary material for: Maternal nutrition intervention and maternal complications in 4 districts of Bangladesh: A nested cross-sectional study
Source: PLoS Med. 2019 Oct 4;16(10):e1002927. doi: 10.1371/journal.pmed.1002927 (PMC6777761; doi:10.1371/journal.pmed.1002927)
Supplement: S1 Study Instruments — (PDF) [file pmed.1002927.s007.pdf]

SK DATA ABSTRACTION FORM
(এসকে ডাটা এবসটাকশন ফরম)

Instructions: Please review the SK register, the patient red booklet, and (if delivered or referred to a hospital) the hospital certificate/record with the SK after the postnatal care visit or at the SK's home/BRAC office. If the record is reviewed while accompanied by the SK at the participant's home, please do so outside of the patient's home or in a private way during the visit while the SK engages the patient and her family. Record the information as noted in these sources. For missing data, please mark the data as missing – do not ask the SK or participant to try to recall the information. For data that seem contradictory to what the participant reported during the interview, ask the SK to clarify with the participant and if any change is needed for the written record, it must be done by the SK.

ভুমিকা:- স্বাস্থ্য কর্মীর রেজিষ্টার পূনরায় রিভিউ/যাচাই করুন। রোগীর লাল বুকলেট(যদি হাসপাতালে রেফার্ড করে) হাসপাতালের সার্টিফিকেট/তথ্য সহ স্বাস্থ্য কর্মীর পূর্বের পি এন সি ভিজিট যাচাই করুন। ভিজিট করার সময় রোগীর বাড়ীর প্রতিবেশী অথবা গোপনীয়ভাবে স্বাস্থ্যকর্মী,রোগী এবং রোগীর পরিবারের সাথে জড়িত আছে কিনা যাচাই করুন। কোথা থেকে এই তথ্য পেয়েছেন তা রেকর্ড রাখা।বাদ পড়া তথ্যের জন্য; বাদ পড়া তথ্য দাগ দিয়ে রাখা। তথ্য সংগ্রহের জন্য স্বাস্থ্যকর্মী বা রোগীকে প্রশ্ন না করা।

For maternal deaths, please complete this form based on the SK register and verbal autopsy report.

মাতৃ মৃত্যুর জন্য, স্বাস্থ্যকর্মীর রেজিস্টার এবং ভারভাল অটোপসী রিপোর্ট অনুযায়ী ফরম পূরন করুন।

No.	Question	Response Options	Response	Skip/Notes
001	District Name<br>(জেলার নাম)	1. Mymensingh<br>ময়মনসিংহ<br>2. Rangpur<br>রংপুর<br>3. Lalmonirhat<br>লালমনিরহাট<br>4. Kurirgam<br>কুড়িগ্রাম	____	
002	Upazila Name<br>(উপজেলার নাম)		____________________	
003	Upazila Condition<br>(উপজেলার ধরন)	1. Intervention<br>(ইন্টারভেনশন)<br>2. Control<br>(কন্ট্রোল)	____	
004	Data Collector Name<br>(তথ্য সংগ্রহকারীর নাম)		___________________	
005	Participant ID Number<br>(তথ্য প্রদানকারীর আই ডি নং)		____ ____ ____ ____	
010	Monthly income of family<br>(পারিবারিক মাসিক আয়)	Amount in taka[Enter 9999 if unknown<br>টাকার পরিমানে লিখুন (তথ্য অজানা থাকলে ৯৯৯ লিখুন)	____ ____ ____ ____<br>taka	

011	Number of household members<br>(পরিবারের সদস্য সংখ্যা)	Total living in the house<br>(বাড়িতে কতজন লোক বাস করে)	________ ________	
Obstetric History & ANC Course				
100	Patient's Age in years at 1st ANC Visit<br>(প্রথম এএনসি ভিজিটের সময় রোগীর বয়স কত ছিল)	Number of years<br>(Mark 99 if no data)<br>সংখ্যায় লিখুন (কোন তথ্য না থাকলে ৯৯ লিখুন)	___________ years	Check logic based on # deliveries
101	Number prior pregnancies (gravida) (including this pregnancy)<br>গর্ভ নং(বর্তমানটি সহ)	Number of pregnancies (Will be 01 if first pregnancy; include miscarriages)<br>গর্ভ সংখ্যা (যদি প্রথম গর্ভ হয়,তবে ০১ হবে;অপরিণত গর্ভ সহঅর্ন্তভুক্ত হবে)	_____ _____	
102	LMP (first date last menstrual period)<br>এল এম পি(শেষ মাসিকের তারিখ)	Day/month/year Gregorian; for single digit month, write 0 first (ex: July=07) If missing, write 99/99/99<br>(দিন/মাস/বছর<br>যদি সংখ্যা একটি হয় তবে তার আগে ০ লিখুন (যেমন জুলাই=০৭)<br>তথ্য না থাকলে ৯৯/৯৯/৯৯ লিখুন)	____ ____/____<br>____/ ____ ____	
103	Estimated Date Delivery (EDD)<br>প্রসবের সম্ভাব্য তারিখ(ইডিডি)	Day/month/year Gregorian; for single digit day or month, write 0 first (ex: July=07). If missing, write 99/99/99<br>(দিন/মাস/বছর<br>যদি সংখ্যা একটি হয় তবে তার আগে ০ লিখুন (যেমন জুলাই=০৭)<br>তথ্য না থাকলে ৯৯/৯৯/৯৯ লিখুন)	____ ____/____<br>____/ ____ ____	
104	Date First ANC Visit by SK<br>(স্বাস্থ্যকর্মীর প্রথম এএনসি ভিজিটের তারিখ)	Day/month/year Gregorian; for single digit day or month, write 0 first (ex: July=07). If missing, write 99/99/99<br>(দিন/মাস/বছর	____ ____/____<br>____/ ____ ____	

		যদি সংখ্যা একটি হয় তবে তার আগে ০ লিখুন (যেমন জুলাই=০৭) তথ্য না থাকলে ৯৯/৯৯/৯৯ লিখুন)		
105	Gestational age at first ANC<br>(প্রথম এএনসি ভিজিটে গর্ভের বয়স)	Use special calendar to calculate based on EDD and date of first visit.<br>(প্রথম এএনসি ভিজিট ও প্রসবের সম্ভাব্য তারিখগণনার মাধ্যমে করে বের করুন)	____ ____ (weeks),<br>____ ____ (days)	Use provided calendar and LMP
106	Blood pressure at first ANC visit<br>প্রথম এএনসি ভিজিটের সময় রক্তচাপ	To record double digit number, make first number 0; for example, 120/60 record as 120/ 060.<br>(দুই সংখ্যার নাম্বার এর জন্য প্রথমে ০ ব্যাবহার করুন যেমন ১২০/৬০ লেখার জন্য ১২০/০৬০ লিখুন)	____ _____ ____/<br>____ ____ ____<br>[Enter 999/999 if missing]	
107	Maternal weight at first ANC visit<br>(প্রথম এএনসি ভিজিটে মায়ের ওজন)	In kg<br>(কেজি তে লিখুন)	_____ _____ _____<br>[Enter 999 if missing]	
108	Hemoglobin number available for first ANC visit?<br>(প্রথম এএনসি ভিজিটে হিমোগ্লোবিন পরিমান)	1. Yes-হ্যাঁ<br>2. No-না	____	If no (2), go to 110.<br>(যদি না(২) হয় তবে ১১০ যেতে হবে ।)<br>.
109	Hemoglobin at first visit (red book)<br>(প্রথম এএনসি ভিজিটে হিমোগ্লোবিন (এমসিএইচএন হ্যান্ড বুক দেখুন)	In gm/dL, watch for decimal point<br>(গ্রাম/ ডিএল এ লিখুন,ডেসিম্যাল পয়েন্ট লক্ষ রাখুন)	_____ _____. _____	
110	Date Last ANC Visit by SK<br>(স্বাস্থ্যকর্মী কর্তৃক শেষ এএনসি ভিজিটের তারিখ)	Day/month/year Gregorian; for single digit day or month, write 0 first (ex: July=07). If missing, write 99/99/99<br>(দিন/মাস/বছর	____ ____/____<br>____/ ____ ____	

		যদি সংখ্যা একটি হয় তবে তার আগে ০ লিখুন (যেমন জুলাই=০৭) তথ্য না থাকলে ৯৯/৯৯/৯৯ লিখুন)		
111	Gestational age at last ANC visit (শেষ এএনসি ভিজিটে গর্ভের বয়স)	Use special calendar to calculate based on EDD and date of last visit (প্রথম এএনসি ভিজিট ও প্রসবের সম্ভাব্য তারিখগণনার মাধ্যমে করে বের করুন)	____ ____ (weeks), ____ ____ (days)	
112	Blood pressure at last ANC visit (শেষ এএনসি ভিজিটের সময় রক্তচাপ)	To record double digit number, make first number 0; for example, 120/60 record as 120/ 060 (দুই সংখ্যার নাম্বার এর জন্য প্রথমে ০ ব্যাবহার করুন যেমন ১২০/৬০ লেখার জন্য ১২০/০৬০ লিখুন)	____ _____ ____/ ____ ____ ____ [Enter 999/999 if missing]	
113	Maternal weight at last ANC visit (শেষ এএনসি ভিজিটে মায়ের ওজন)	In kg (কেজি তে লিখুন)	____ ____ ____ [Enter 999 if missing]	
114	Hemoglobin number available for last ANC visit(শেষ এএনসি ভিজিটে হিমোগ্লোবিন পরিমান)	1. Yes- হ্যাঁ 2. No- না	____	If no (2), go to 116. (যদি না হয়, তবে ১১৬ যেতে হবে ।)
115	Hemoglobin at last visit (red book) (শেষ এএনসি ভিজিটে হিমোগ্লোবিন (এমসিএইচ হ্যান্ড বুক দেখুন)	In gm/dL, watch for decimal point (গ্রাম/ ডিএল এ লিখুন,ডেসিম্যাল পয়েন্ট লক্ষ রাখুন)	_____ _____. _____	
116	Date last ANC hemoglobin (শেষ এএনসি ভিজিটে হিমোগ্লোবিন পরিমাপের তারিখ)	Day/month/year Gregorian; for single digit day or month, write 0 first (ex: July=07). If missing, write 99/99/99(দিন/মাস/বছর যদি সংখ্যা একটি হয় তবে তার আগে ০ লিখুন (যেমন জুলাই=০৭) তথ্য না থাকলে ৯৯/৯৯/৯৯ লিখুন)	____ ____/____ ____/ ____ ____	

117	Gestational age at last ANC hemoglobin<br>(শেষ এএনসি ভিজিটে হিমোগ্লোবিন পরিমাপেরতারিখের দিন গর্ভের বয়স)	Use special calendar to calculate based on EDD and date of Hgb measure<br>(প্রথম এএনসি ভিজিট ও প্রসবের সম্ভাব্য তারিখগণনার মাধ্যমে করে বের করুন)	____ ____ (weeks),<br>____ ____ (days)	
118	Was urine dipstick done at last ANC visit?<br>(শেষ এএনসি ভিজিটে পস্রাব পরীক্ষা করা হয়েছে কি?)	1. Yes- হ্যাঁ<br>2. No- না	____	If no, go to 120.<br>(যদি না হয়, তবে ১২০ যেতে হবে।)
119	Urine dip results at last ANC visit<br>(শেষ এএনসি ভিজিটে পস্রাব পরীক্ষার ফলাফল)	1.Negative for albumin and glucose<br>(এলবুমিন এবং সুগার নাই)<br>2. Positive for albumin only<br>(শুধুমাত্র এলবুমিন আছে)<br>3.Positive for glucose only<br>(শুধুমাত্র সুগার আছে)<br>4. Positive for both albumin and glucose(এলবুমিন এবং সুগার উভয়ই আছে)	____	
120	Complications reported during pregnancy: Review all ANC visits in SK register and red book before responding. Multiple answers permitted as applicable<br>(গর্ভকালীন সময়ে কোন জটিলতা থেকে থাকলে ঃ স্বাস্থ্যকর্মী কর্তৃক সকল এএনসি ভিজিট এবং এমসিএইচ হ্যান্ড বুক যাচাই করুন।)<br>(একাধিক উত্তর গ্রহন যোগ্য)	1.No complications<br>(কোন জটিলতা ছিল না)<br>2.Severe nausea/vomiting(বমি বমি ভাব/বমি হওয়া)<br>3.High blood pressure<br>(উচ্চ রক্তচাপ)<br>4.Severe edema<br>(হাত-পায়ের পাতায় পানি আসা)<br>5.Low weight gain<br>(ওজন কম বাড়া)<br>6.Gestational diabetes(গর্ভকালীন সময়ে ডায়াবেটিস)<br>7.Fever/infections(জ্বর/সংক্রমন)<br>8.Vaginal bleeding(যোনিপথে রক্তপাত)<br>9.Preterm labor<br>(নির্ধারিত সময়ের পূর্বে প্রশব ব্যাথা)	____ ____<br>____ ____<br>____ ____<br>____ ____<br>[If single digit, add 0 as first number. Ex: Severe edema=04]<br>If need to specify:<br>___________________	

		10. Leakage of fluid/preterm rupture of membranes<br>(পানি বের হয়ে যাওয়া/........)<br>11.Symptomatic anemia(রক্তাল্পতার লক্ষণ)<br>12.Other (specify):অন্যান্য(উল্লেখ্যকরুন)	____________________<br>_	
121	Was patient referred to hospital for any complication during pregnancy? [Consult red book]<br>(গর্ভ কালীন সময়ে কোন জটিলতার কারনে রোগীকে কি হাসপাতালে পাঠানো হয়েছিল?)<br>(এমসিএইচএন হ্যান্ড বুক দেখুন)	1. Yes- হ্যাঁ<br>2. No- না	____	If no (2), go to 123<br>(যদি না (২) হয়, তাহলে ১২৩ যেতে হবে)
122	If yes, dates of hospitalization/referral visit<br>(যদি হাঁ হয় তাহলে হাসপাতালে গমন/ভর্তির তারিখ)	Day/month/year Gregorian; for single digit month or day, write 0 first (ex: July=07). If missing, write 99/99/99<br>(দিন/মাস/বছর<br>যদি সংখ্যা একটি হয় তবে তার আগে ০ লিখুন (যেমন জুলাই=০৭)<br>তথ্য না থাকলে ৯৯/৯৯/৯৯ লিখুন)	_____ _____/_____<br>_____/ _____ _____	
123	Number doses tetanus toxoid vaccination received<br>(টিটি টীকার কততম ডোজ নিয়েছে)	Count in register or red book<br>(রেজিস্টার অথবা এমসিএইচএন হ্যান্ড বুক দেখুন)	____	
124	Total number of ANC visits at facility<br>(স্বাস্থ্য কেন্দ্রে কতবার এএনসি ভিজিটে যাওয়া হয়েছিলো)	Count in red book<br>(এমসিএইচএন হ্যান্ড বুক দেখে যোগ করুন)	____	
125	Total number ANC visits by SK<br>(স্বাস্থ্য কর্মী দ্বারা কতবার এএনসি ভিজিট করা হয়েছিলো)	Count in register<br>(রেজিস্টারদেখে যোগ করুন)	____ ____	

Nutrition Indicators				
200	At which month ANC visit were IFA tablets consumed first recorded?<br>(কোন মাসের এএনসি ভিজিট এ প্রথম আয়রন ট্যাবলেট দেয়া হয়েছিল)	Enter visit month where number of tablets eaten is first recorded.	____ ____	
201	Number IFA tablets reported consumed at first recorded ANC entry<br>(প্রথম রেকর্ডকৃত মাসে আয়রন ট্যাবলেট খাওয়ার সংখ্যা)	Enter total from register; enter 99 for missing data<br>(মোট সংখ্যা টি রেজিস্টার দেখে যোগ করুন। তথ্য না থাকলে ৯৯ লিখুন।)	____ ____	
202	Number IFA tablets reported consumed at last recorded ANC entry<br>(শেষ রেকর্ডকৃত মাসে আয়রন ট্যাবলেট খাওয়ার সংখ্যা)	Enter total from register; enter 99 for missing data<br>(মোট সংখ্যা টি রেজিস্টার দেখে যোগ করুন। তথ্য না থাকলে ৯৯ লিখুন।)	____ ____	
203	At which month of ANC visit were calcium tablets consumed first recorded?<br>(কোন মাসের এএনসি ভিজিট এ প্রথম ক্যালসিয়াম ট্যাবলেট দেয়া হয়েছিল)	At which month ANC visit were calcium tablets consumed first recorded?<br>(প্রথম রেকর্ডের কোন মাস থেকে খাওয়া শুরু করেছে তা লিখুন।)	____ ____	
204	Number calcium tablets reported consumed at first recorded ANC entry<br>(প্রথম রেকর্ডকৃত মাসে ক্যালসিয়াম ট্যাবলেট খাওয়ার সংখ্যা)	Enter total from register; enter 99 for missing data<br>(মোট সংখ্যা টি রেজিস্টার দেখে যোগ করুন। তথ্য না থাকলে ৯৯ লিখুন।)	____ ____	
205	Number calcium tablets reported consumed at last recorded ANC entry<br>(শেষ ক্যালসিয়াম মাসে ক্যালসিয়াম ট্যাবলেট খাওয়ার সংখ্যা)	Enter total from register; enter 99 for missing data<br>(মোট সংখ্যা টি রেজিস্টার দেখে যোগ করুন। তথ্য না থাকলে ৯৯ লিখুন।)	____ ____	If control upazila, go to 300.<br>(যদি কন্ট্রোল উপজেলা হয়, তবে ৩০০ যেতে হবে।)

For intervention upazilas only (see SK register) at first ANC visit:				
206	FishMeat/Liver taken within lhours (গত ২৪ ঘন্টায় মাছ, মাংস এবং কলিজা খেয়েছেন?)	1. Yes- হ্যাঁ<br>2. No- না<br>9. Data missing- (তথ্য বাদ পড়েছে)	____	If no, go to 208. (যদি না হয়, তবে ২০৮ যেতে হবে।)
207	Amount (bowl) {পরিমান(বাটিতে)}	Enter stated number of bowl(s), or 99 for missing data (পরিমান বোলে লিখুন, বাধ পড়া তথ্যের জন্য ৯৯ চাপুন)	_____ _____	
208	Egg taken within 24 hours (গত ২৪ ঘন্টায় ডিম খেয়েছেন?)	1. Yes- হ্যাঁ<br>2. No-না<br>9. Data missing (তথ্য বাদ পড়েছে)	____	If no, go to 210. (যদি না হয তবে ২১০ যেতে হবে্)
209	Amount (bowl) [পরিমান(বাটিতে)]	Enter stated number of bowl(s), or 99 for missing data (পরিমান বোলে লিখুন, বাধ পড়া তথ্যের জন্য ৯৯ লিখুন)	_____ _____	
210	Milk /Milk Products taken within last 24 hours (২৪ ঘন্টায় দুধ/ দুধের তৈরী খাবার খেয়েছেন?)	1. Yes- হ্যাঁ<br>2. No- না<br>9. Data missing (তথ্য বাদ পড়েছে)	____	If no/missing, go to 212. (যদি না হয়/বাদ পড়া তথ্যের জন্য ২১২ যেতে হবে।)
211	Amount (bowl) [পরিমান(বাটিতে)]	Enter stated number of bowl(s), or 99 for missing data (পরিমান বোলে লিখুন, বাধ পড়া তথ্যের জন্য ৯৯ লিখুন)	_____ _____	
212	Orange/yellow vegetable/fruit taken within last 24 hours (গত ২৪ ঘন্টায় কমলা /হলুদ সব্জি/ফল খেয়েছেন?)	1. Yes- হ্যাঁ<br>2. No- না<br>9. Data missing (তথ্য বাদ পড়েছে)	____	If no/ missing, go to 214. (যদি না হয়/বাদ পড়া তথ্যের জন্য

				২১৪ যেতে হবে।)
213	Amount (bowl)<br>[পরিমান(বাটিতে)]	Enter stated number of bowl(s), or 99 for missing data<br>(পরিমান বোলে লিখুন, বাধ পড়া তথ্যের জন্য ৯৯ চাপুন)	_____ _____	
214	Rice/ Bread taken within last 24 hours<br>(গত ২৪ ঘন্টায় ভাত/রুটি খেয়েছেন?)	1. Yes- হ্যাঁ<br>2. No- না<br>9. Data missing<br>(তথ্য বাদ পড়েছে)	____	If no/ missing, go to 216.<br>(যদি না হয়/ বাদ পড়া তথ্যের জন্য ২১৬ যেতে হবে।)
215	Amount (bowl)<br>[পরিমান(বাটিতে)]	Enter stated number of bowl(s), or 99 for missing data<br>(পরিমান বোলে লিখুন, বাধ পড়া তথ্যের জন্য ৯৯ লিখুন)	_____ _____	
216	Dark Green Vegetables taken within last 24 hours<br>(গত ২৪ ঘন্টায় গাঢ় সবুজ শাক খেয়েছেন?)	1. Yes- হ্যাঁ<br>2. No- না<br>9. Data missing<br>(তথ্য বাদ পড়েছে)	____	If no/ missing, go to 218.<br>(যদি না হয়/বাদ পড়া তথ্যের জন্য ২১৮ যেতে হবে।)
217	Amount (bowl)<br>[পরিমান(বাটিতে)]	Enter stated number of bowl(s), or 99 for missing data<br>(পরিমান বোলে লিখুন, বাধ পড়া তথ্যের জন্য ৯৯ লিখুন)	_____ _____	
218	Thick Lentils taken within last 24 hours<br>(গত ২৪ ঘন্টায় ঘন ডাল খেয়েছেন?)	1. Yes- হ্যাঁ<br>2. No- না<br>9. Data missing<br>(তথ্য বাদ পড়েছে)	____	If no/ missing, go to 220.<br>(যদি না হয়/বাদ পড়া তথ্যের জন্য ২২০ যেতে হবে।)

219	Amount (bowl)<br>[পরিমান(বাটিতে)]	Enter stated number of bowl(s), or 99 for missing data<br>(পরিমান বোলে লিখুন, বাধ পড়া তথ্যের জন্য ৯৯ লিখুন)	_____ _____	
For intervention upazilas only (see SK register) at last ANC visit:				
220	Fish/ Meat/Liver taken within last 24 hours<br>(গত২৪ ঘন্টায় মাছ/ মাংস/ কলিজা খেয়েছেন?)	1. Yes- হ্যাঁ<br>2. No- না<br>9. Data missing<br>(তথ্য বাদ পড়েছে)	____	If no/ missing, go to 222(যদি না হয়/বাদ পড়া তথ্যের জন্য ২২২ যেতে হবে।)
221	Amount (bowl)<br>[পরিমান(বাটিতে)]	Enter stated number of bowl(s), or 99 for missing data<br>(পরিমান বোলে লিখুন, বাধ পড়া তথ্যের জন্য ৯৯ লিখুন)	_____ _____	
222	Egg taken within 24 hours<br>(গত ২৪ ঘন্টায় ডিম খেয়েছেন?)	1. Yes- হ্যাঁ<br>2. No- না<br>9. Data missing<br>(তথ্য বাদ পড়েছে)	____	If no/ missing, go to 224.<br>(যদি না হয়/বাদ পড়া তথ্যের জন্য ২২৪ যেতে হবে।)
223	Amount (bowl)<br>[পরিমান(বাটিতে)]	Enter stated number of bowl(s), or 99 for missing data<br>(পরিমান বোলে লিখুন, বাধ পড়া তথ্যের জন্য ৯৯ লিখুন)	_____ _____	
224	Milk /Milk Products taken within last 24 hours<br>(গত ২৪ ঘন্টায় দুধ/ দুধের তৈরী খাবার খেয়েছেন?)	1. Yes- হ্যাঁ<br>2. No- না<br>9. Data missing<br>(তথ্য বাদ পড়েছে)	____	If no/ missing, go to 226.<br>(যদি না হয়/বাদ পড়া তথ্যের জন্য ২২৬ যেতে হবে।)

225	Amount (bowl)<br>[পরিমান(বাটিতে)]	Enter stated number of bowl(s), or 99 for missing data<br>(পরিমান বোলে লিখুন, বাধ পড়া তথ্যের জন্য ৯৯ লিখুন)	_____ _____	
226	Orange/yellow vegetable/fruit taken within last 24 hours<br>(গত ২৪ ঘন্টায় কমলা/হলুদ সব্জি/ফল খেয়েছেন?)	1. Yes- হ্যাঁ<br>2. No- না<br>9. Data missing<br>(তথ্য বাদ পড়েছে)	____	If no/ missing, go to 228.<br>(যদি না হয়/বাদ পড়া তথ্যের জন্য ২২৮ যেতে হবে।)
227	Amount (bowl)<br>[পরিমান(বাটিতে)]	Enter stated number of bowl(s), or 99 for missing data<br>(পরিমান বোলে লিখুন, বাধ পড়া তথ্যের জন্য ৯৯ লিখুন)	_____ _____	
228	Rice/ Bread taken within last 24 hours<br>(গত ২৪ ঘন্টায় ভাত/রুটি খেয়েছেন?)	1. Yes- হ্যাঁ<br>2. No- না<br>9. Data missing<br>(তথ্য বাদ পড়েছে)	____	If no/ missing, go to 230.<br>(যদি না হয়/বাদ পড়া তথ্যের জন্য ২৩০ যেতে হবে।)
229	Amount (bowl)<br>[পরিমান(বাটিতে)]	Enter stated number of bowl(s), or 99 for missing data<br>(পরিমান বোলে লিখুন, বাধ পড়া তথ্যের জন্য ৯৯ লিখুন)	_____ _____	
230	Dark Green Vegetables taken within last 24 hours<br>(গত ২৪ ঘন্টায় গাঢ় সবুজ শাক খেয়েছেন?)	1. Yes- হ্যাঁ<br>2. No- না<br>9. Data missing<br>(তথ্য বাদ পড়েছে)	____	If no/ missing, go to 232.(যদি না হয়/বাদ পড়া তথ্যের জন্য ২৩২ যেতে হবে।)
231	Amount (bowl)<br>[পরিমান(বাটিতে)]	Enter stated number of bowl(s), or 99 for missing data	_____ _____	

		(পরিমান বোলে লিখুন, বাধ পড়া তথ্যের জন্য ৯৯ লিখুন)		
232	Thick Lentils taken within last 24 hours (গত ২৪ ঘন্টায় ঘন যাল খেয়েছেন?)	1. Yes- হ্যাঁ<br>2. No- না<br>9. Data missing (তথ্য বাদ পড়েছে)	____	If no/ missing, go to 300. (যদি না হয়/বাদ পড়া তথ্যের জন্য ৩০০ যেতে হবে।)
233	Amount (bowl) [পরিমান(বাটিতে)]	Enter stated number of bowl(s), or 99 for missing data (পরিমান বোলে লিখুন, বাধ পড়া তথ্যের জন্য ৯৯ লিখুন)	_____ _____	
Delivery/Postpartum Period Information				
|	Date of delivery (প্রসবের তারিখ)	Day/month/year Gregorian; for single digit month or day, write 0 first (ex: July=07). If missing, write 99/99/99 (দিন/মাস/বছর। বাদ পড়া তথ্যের জন্য ৯৯/৯৯/৯৯ লিখুন)	_____ _____/_____ _____/ _____ _____	Logic check with EDD/LMP.(ইডিডি/এলএমপি চেক করতে হবে।)
301	Place of delivery (প্রসবের স্থান)	1. Patient's home (নিজের বাড়ী)<br>2. Patient's mother's home (বাবার বাড়ী)<br>3. Midwife's home (ধাত্রী বাড়ী)<br>4. Hospital (হাসপাতাল)<br>5. Other (specify): [অন্যান্য(নির্দিষ্ট করুন)]<br>6. Data missing (তথ্য বাদ পড়েছে)	_____<br>Specify:(নির্দিষ্টকরুন)_____ _______________	
302	Skilled birth attendant present? (এস বি এ উপস্থিত ছিল?)	1. Yes- হ্যাঁ<br>2. No- না<br>3. Data missing (তথ্য বাদ পড়েছে)	_____	
303	Type of delivery	1. Vaginal		

	(প্রসবের ধরন)	স্বাভাবিক<br>2. Cesarean section সিজার<br>3. Assisted vaginal (vacuum) ভেকুম	______	
304	Misoprostol received for PPH prevention? (প্রসবের সাথে সাথেই অতিরিক্ত রক্ত ক্ষরন বন্ধের জন্য মিসোপ্রস্টাল খেয়েছেন?)	1. Yes- হ্যাঁ<br>2. No- না<br>9. Data missing (তথ্য বাদ পড়েছে)	______	
305	Maternal outcome(মায়ের অবস্থা)	1. Living (জীবিত)<br>2. Death during labor/delivery (প্রসবের সময় মারা গেছে)<br>3. Death after delivery (প্রসবের পর মারা গেছে)	______	If 2 or 3, go to 500. (যদি ২ অথবা ৩ হয় তাহলে ৫০০ যেতে হবে।)
306	Complications during labor/delivery? (প্রসবের সময় কোন জটিলতা ছিল?)	1. Yes- হ্যাঁ<br>2. No- না<br>9. Data missing (তথ্য বাদ পড়েছে)	______	If no, go to 310. (যদি না হয়, তবে ৩১০ যেতে হবে।)
307	What complications are noted in the register? Multiple answers permitted as applicable. (রেজিষ্টারে কি ধরনের জটিলতা উল্লেখ আছে? একাধিক উত্তর প্রযোজ্য।)	1. Pre-eclampsia (প্রি-একলাম্পসিয়া)<br>2. Eclampsia/convulsions (একলাম্পশিয়া)<br>3. Heavy bleeding during labor (প্রসবের সময় অতিরিক্ত রক্ত ক্ষরন)<br>4. Heavy bleeding after delivery (প্রসবের পর অতিরিক্ত রক্ত ক্ষরন)<br>5. Prolonged labor/insufficient cervical dilation(দীর্ঘায়িত প্রসব ব্যথা/)<br>6. Prolonged labor, pushing more than 2 hours(দীর্ঘায়িত প্রসব	____ ____<br>____ ____<br>____ ____<br>____ ____<br>[If single digit, add 0 as first number. Ex: Obstructed labor=07]<br>If need to specify:<br>____________________<br>____________________	

		ব্যথা, ২ ঘন্টার অধিক সময় ধরেচেষ্টা)<br>7. Obstructed labor (বাধাঁ গ্রস্থ প্রসব)<br>8. Breech/non-cephalic (বাচ্চার মাথা উপরের দিকে)<br>9. Fever/infection in labor (প্রসবের সময় জ্বর/সংক্রামন)<br>10. Baby not breathing well (বাচ্চা শ্বাস প্রশ্বাস ঠিকমত নিচ্ছে না)<br>11. Retained placenta (গর্ভফুল আটকে যাওয়া)<br>12. Other (specify): [অন্যান্য(নির্দিষ্ট করুন)]		
308	Was patient referred to hospital for complications? (এই জটিলতার সমূহের কারনে মা হাসপাতালে গিয়েছিল?)	1. Yes- হ্যাঁ<br>2. No- না	____	
309	Did the patient go to the hospital when referred? (রেফার্ড করার পর হাসপাতালে গিয়েছিলেন?)	1. Yes- হ্যাঁ<br>2. No- না<br>[Ask for hospital certificate/record]	____	
310	Neonatal outcome (নবজাতকের অবস্থা)	1. Stillbirth - macerated (গলিত মৃত প্রসব)<br>2. Stillbirth – fresh(স্বাভাবিক মৃত প্রসব)<br>3. Live born, preterm (before 9th month) [অপরিনত জীবিত জন্ম(৯ মাসের পূর্বে)]<br>4. Live born, term (পরিনত জীবিত জন্ম)	____	If 1 or 2, go to 314. (যদি ১ অথবা ২ হয়, তবে ৩১৪ যেতে হবে।)
311	If live born, weight of baby at birth (যদি জীবিত জন্ম হয়, বাচ্চার জন্ম ওজন)	Enter weight in grams; enter 9999 if missing. (গ্রামে ওজন লিখুন, বাদ পড়া তথ্যের জন্য ৯৯৯৯ লিখুন).	____ ____ ____ ____grams	

312	Breastfeeding within 1 hour of delivery?<br>(প্রসবের ১ ঘন্টার মধ্যে বাচ্চাকে শাল দুধ খাওয়ানো হয়েছিল?)	1. Yes- হ্যাঁ<br>2. No- না<br>9. Data missing<br>(তথ্য বাদ পড়েছে)	____	
313	Exclusively breastfeeding at first PNC visit?(<br>প্রথম পি এন সিতে শুধুমাত্র বুকের দুধ খাওয়ানো হয়েছিল?)	1. Yes- হ্যাঁ<br>2. No- না<br>9. Data missing<br>(তথ্য বাদ পড়েছে)	____	
314	Date first PNC visit<br>(প্রথম পি এন সি পরিদর্শনে তারিখ)	Day/month/year Gregorian; for single digit month or day, write 0 first (ex: July=07). If missing, write 99/99/99<br>(দিন/মাস/। বাদ পড়া তথ্যের জন্য ৯৯/৯৯/৯৯ লিখুন)	_____ _____/_____ _____/ _____ _____	
315	Blood pressure at first PNC visit<br>(প্রথম পি এন সি পরিদর্শনে রক্ত চাপ?)	To record double digit number, make first number 0; for example, 120/60 record as 120/ 060	____ _____ ____/ ____ ____ ____<br>[Enter 999/999 for missing data]	
316	Hemoglobin number available for PNC visit?<br>(পি এন সি পরিদর্শনে হিমোগ্লোবিনের পরিমান লিখা রয়েছে কি না)	1. Yes- হ্যাঁ<br>2. No- না	____	
317	Hemoglobin at PNC visit<br>(পি এন সি পরিদর্শনে হিমোগ্লোবিনের পরিমান)	In gm/dL, watch for decimal point	_____ _____. _____	
318	Number PNC visits to date?<br>(এ পর্যন্ত পি এন সি পরিদর্শনের সংখ্য)া	Add total number from register, including today's visit.<br>(মোট সংখ্যা আজকের দিন সহ রেজিষ্টার থেকে লিখুন)	_____	
319	Any complications during the postpartum period?<br>(প্রসবের পরবর্তি সময়ে কোন জটিলতা ছিল?)	1. Yes- হ্যাঁ<br>2. No- না	_____	If no, go to 400.<br>(যদি না হয়, তবে ৪০০ যেতে হবে।)
320	What postpartum complications are noted in the register/red book;	1. Pre-eclampsia (প্রি-একলাম্পসিয়া)<br>2. Eclampsia/convulsions (একলাম্পসিয়া)	_____	

	multiple answers permitted as applicable? (প্রসব পরবর্তি সময়ে রেজিষ্টার/লাল বইয়ে কি ধরনের জটিলতা উল্লেখ ছিল? একাধিক উত্তর প্রযোজ্য,)	3. Heavy bleeding after delivery (প্রসবের পর অতিরিক্ত রক্ত ক্ষরন)<br>4. Fever/infection (জ্বর/সংক্রামন)<br>5. Baby not breathing well (বাচ্চা সঠিকভাবে শ্বাস-প্রশ্বাস নিচ্ছে না)<br>6. Baby not nursing well (বাচ্চার সঠিক যত্ন হয় নাই)<br>7. No milk production<br>8. (মায়ের দুধ তৈরী হচ্ছে না)<br>9. Other (specify): অন্যান্য (নির্দিষ্ট করুন)	_____<br>_____<br>If need to specify:<br>____________________<br>____________________	
321	Was patient referred to hospital for postpartum complications? (প্রসব পরবর্তি এই জটিলতার জন্য হাসপাতালে রেফার্ড করা হয়েছে?)	1. Yes- হ্যাঁ<br>2. No- না	_____	
322	Did the patient go to the hospital when referred? (রেফার্ড করার পর হাসপাতালে গিয়েছিল?)	1. Yes- হ্যাঁ<br>2. No- না	_____	
323	Selected /plans to use family planning method? (পরিবার পরিকল্পনা পদ্ধতি গ্রহনের পরিকল্পনা করেছে?)	1. Yes- হ্যাঁ<br>2. No- না<br>9. Data missing (তথ্য বাদ পড়েছে)	_____	
324	Status of infant at PNC visit (পি এন সি পরিদর্শনে শিশুর অবস্থা)	1. Alive and well (জীবিত এবং সুস্থ্য)<br>2. Alive and sick (জীবিত এবং অসুস্থ্য)<br>3. Death within 48 hours birth (জন্মের৪৮ ঘন্টার মধ্যে মৃত্যু)	_____	If 1 or 2, go to 400. (যদি ১ অথবা ২ হয়, তবে ৪০০ যেতে হবে।)

		4. Death between 48 hours of birth and PNC visit (জন্মের ৪৮ ঘন্টা পর মৃত্যু এবং পি এন সি পরিদর্শন)		
325	Cause of infant death [Consult verbal autopsy form if available; multiple answers permitted as applicable] (শিশু মৃত্যুর কারন। ভারভাল অটোপসি রিপোর্ট যদি থাকে, একাধিক উত্তর গ্রহনযোগ্য।)	1. Birth asphyxia (জন্ম গত শ্বাস কষ্ট)<br>2. Sepsis/infection from labor(পচন/সংক্রামক)<br>3. Cord infection (শ্বাস তন্ত্রের সংক্রামন)<br>4. Prematurity (অপরিনত)<br>5. Dehydration (পানি শুন্যতা)<br>6. Other (specify): অন্যান্য (নির্দিষ্ট করুন)<br>7. Unknown (অজানা)	_____<br>_____<br>_____	
326	Date of infant death (শিশু মৃতুর কারন)	Day/month/year Gregorian; for single digit month or day, write 0 first (ex: July=07). If missing, write 99/99/99 (দিন/মাস/বছর । বাদ পড়া তথ্যের জন্য ৯৯/৯৯/৯৯ লিখুন)	_____ _____/_____ _____/ _____ _____	
Nutrition Indicators- পুষ্টির নির্দেশক				
400	Number IFA tablets reported consumed at last recorded PNC entry(শেষ পি এন সি ভিজিটে কতটি আয়রন বড়ি খেয়েছে খেয়েছে বলে উলে-খ রয়েছে?)	Enter total from register; enter 99 for missing data	____ ____	
401	Number calcium tablets reported consumed at last recorded PNC entry (শেষ পি এন সি তথ্য লিপিবদ্ধে কতটা ক্যালসিয়াম বড়ি খেয়েছে খেয়েছে বলে উলে-খ রয়েছে?)	Enter total from register; enter 99 for missing data	____ ____	If control area or visit before 45 days for Intervention, FINISH
For Intervention Participants At 45 Day PNC Visit Only: Record responses for below questions from today's PNC visit (শুধুমাত্র ইন্টারভেনশন এরিয়ায় ৪৫ দিনের পি এন সি মায়ের জন)				
402	Fish/ Meat/Liver taken within last 24 hours	1. Yes- হ্যাঁ<br>2. No- না	____	If no/ missing,

	(গত২৪ ঘন্টায় মাছ/ মাংস/ কলিজা খেয়েছেন?)	9. Data missing<br>(তথ্য বাদ পড়েছে)		go to 404.(যদি না হয়/বাদ পড়া তথ্যের জন্য ৪০৪ যেতে হবে।)
403	Amount (bowl)<br>[পরিমান(বাটিতে)]	Enter stated number of bowl(s), or 99 for missing data<br>(পরিমান বোলে লিখুন, বাধ পড়া তথ্যের জন্য ৯৯ লিখুন)	_____ _____	
404	Egg taken within 24 hours<br>(গত ২৪ ঘন্টায় ডিম খেয়েছেন?)	1. Yes- হ্যাঁ<br>2. No- না<br>9. Data missing<br>(তথ্য বাদ পড়েছে)	____	If no/ missing, go to 406.(যদি না হয়/বাদ পড়া তথ্যের জন্য ৪০৬ যেতে হবে।)
405	Amount<br>[পরিমান]	Enter stated number of bowl(s), or 99 for missing data<br>(পরিমান লিখুন, বাদ পড়া তথ্যের জন্য ৯৯ লিখুন)	_____ _____	
406	Milk /Milk Products taken within last 24 hours<br>(গত ২৪ ঘন্টায় দুধ/দুধের তৈরী খাবার খেয়েছেন?)	1. Yes- হ্যাঁ<br>2. No- না<br>9. Data missing<br>(তথ্য বাদ পড়েছে)	____	If no/ missing, go to 408.(যদি না হয়/বাদ পড়া তথ্যের জন্য ৪০৮ যেতে হবে।)
407	Amount (bowl)<br>[পরিমান(বাটিতে)]	Enter stated number of bowl(s), or 99 for missing data<br>(পরিমান বোলে লিখুন, বাধ পড়া তথ্যের জন্য ৯৯ লিখুন)	_____ _____	
408	Orange/yellow vegetable/fruit taken within last 24 hours<br>(গত ২৪ ঘন্টায় কমলা/হলুদ সজ্বি/ফল খেয়েছেন?)	1. Yes- হ্যাঁ<br>2. No- না<br>9. Data missing<br>(তথ্য বাদ পড়েছে)	____	If no/ missing, go to 410.(যদি না হয়/বাদ পড়া তথ্যের জন্য

				৪১০ যেতে হবে।)
409	Amount (bowl)<br>[পরিমান(বাটিতে)]	Enter stated number of bowl(s), or 99 for missing data<br>(পরিমান বোলে লিখুন, বাধ পড়া তথ্যের জন্য ৯৯ লিখুন)	_____ _____	
410	Rice/ Bread taken within last 24 hours<br>(গত ২৪ ঘন্টায় ভাত/রুটি খেয়েছেন?)	1. Yes- হ্যাঁ<br>2. No- না<br>9. Data missing<br>(তথ্য বাদ পড়েছে)	____	If no/ missing, go to 412.(যদি না হয়/বাদ পড়া তথ্যের জন্য ৪১২ যেতে হবে।)
411	Amount (bowl)<br>[পরিমান(বাটিতে)]	Enter stated number of bowl(s), or 99 for missing data<br>(পরিমান বোলে লিখুন, বাধ পড়া তথ্যের জন্য ৯৯ লিখুন)	_____ _____	
412	Dark Green Vegetables taken within last 24 hours<br>(গত ২৪ ঘন্টায় গাঢ় সবুজ শাক সবজি খেয়েছেন?)	1. Yes- হ্যাঁ<br>2. No- না<br>9. Data missing<br>(তথ্য বাদ পড়েছে)	____	If no/ missing, go to 414 (যদি না হয়/বাদ পড়া তথ্যের জন্য ৪১৪ যেতে হবে।)
413	Amount (bowl)<br>[পরিমান(বাটিতে)]	Enter stated number of bowl(s), or 99 for missing data<br>(পরিমান বোলে লিখুন, বাধ পড়া তথ্যের জন্য ৯৯ লিখুন)	_____ _____	
414	Thick Lentils taken within last 24 hours<br>(গত ২৪ ঘন্টায় ঘন ডাল খেয়েছেন?)<br>Thick Lentils taken within last 24 hours	1. Yes- হ্যাঁ<br>2.No- না<br>9. Data missing<br>(তথ্য বাদ পড়েছে)	____	If no, FINISH and go to participant interview.
415	Amount (bowl)<br>[পরিমান(বাটিতে)]	Enter stated number of bowl(s), or 99 for missing data<br>(পরিমান বোলে লিখুন, বাধ পড়া তথ্যের জন্য ৯৯ লিখুন)	_____ _____	FINISH and go to participant interview.

Maternal Death Information – Complete only if 305 = 2 or 3.মাতৃ মৃত্যুর তথ্য				
500	Date of maternal death (মাতৃ মৃত্যুর তারিখ)	Day/month/year Gregorian; for single digit month or day, write 0 first (ex: July=07). If missing, write 99/99/99 (দিন/ মাস/বছর। বাদ পড়া তথ্যেও জন্য ৯৯/৯৯/৯৯ লিখুন)	_____ _____/_____ _____/ _____ _____	
501	Site of maternal death (মাতৃ মৃত্যুর স্থান)	1. Patient's home (নিজের বাড়ী)<br>2. Mother's home (মায়ের বাড়ী)<br>3. En route to facility (সেবা গ্রহনের পথে)<br>4. At hospital (হাসপাতালে)<br>9. Data missing (তথ্য বাদ পড়েছে)	____	
502	Cause of maternal death (take from SK register and confirm with verbal autopsy form; multiple answers permitted as applicable) (মাতৃ মৃত্যুর কারন (স্বাস্থ্য কর্মীর রেজিষ্টার এবং মৌখিক অটোপসি রিপোর্ট থেকে তথ্য নিতে হবে, একাধিক তথ্য গ্রহরযোগ্য)	1. Hemorrhage before delivery (প্রসবের পর্বে রক্ত ক্ষরন)<br>2. Hemorrhage after delivery (প্রসবের পর রক্ত ক্ষরন)<br>3. Uterine rupture (জরায়ু ফেটে যাওয়া)<br>4. Pre-eclampsia/eclampsia (প্রাথমিক খিচুনী/ খিচুনী)<br>5. Sepsis/infection (পচন/সংক্রামন)<br>6. Other (specify): [অন্যান্য(নির্দিষ্ট করুন)]	____<br>____<br>____<br>If need to specify:<br>___________________<br>___________________	
503	Neonatal outcome (নবজাতকের অবস্থা)	1. Stillbirth - macerated (গলিত মৃত জন্ম)<br>2. Stillbirth – fresh (স্বাভাবিক মৃত প্রসব)<br>3. Live born, preterm (অপরিনত জীতি জন্ম)<br>4. Live born, term (পরিনত জীবিত জন্ম)	_____	

RECENTLY DELIVERED WOMEN (Within 42-60 days of delivery) INTERVIEW FORM
(৪২-৬০ দিনের প্রসূতি মায়েদের সাক্ষাৎকার ফরম)

Instructions: Please accompany the SK to the recently delivered woman home for the planned PNC visit. Please have the SK introduce you and then ask the patient and her family if she would be willing to speak with you briefly and answer some questions about her medical history and pregnancy and birth experiences. If she agrees, please proceed with informed consent and then administer the following questionnaire – both of these activities should be done with just the woman present.

ভুমিকাঃ দয়া করে এসকের সাথে তার শিডিউল পি এ্যান সি ভিজিট এ প্রসূতি মায়ের বাড়িতে যান। এসকে কে বলুন আপনাকে প্রসূতির পরিবারের সাথে পরিচয় করিয়ে দিতে এবং আপুনি প্রসূতির গর্ভকালীন, প্রসব কালীন এবং প্রসব পরবর্তী সময়ে কোন সমস্যা থেকে থাকলে তা যানতে চাবার জন্য অনুমুতি নিন। যদি সে রাজি থাকে তাহলে, প্রথমে তার সম্মতি পত্র নিন তারপর নিন্মে লিখিত প্রশ্ন গুলো করুন।

No.	Question	Response Options	Response	Skip Pattern/Notes
001	District Name(জেলার নাম)	1. Mymensingh (ময়মনসিংহ)<br>2. Rangpur(রংপুর)<br>3. Lalmonirhat (লালমনিরহাট)<br>4. Kurirgam(কুড়িগ্রাম)	___	
002	Upazila Name(উপজেলার নাম)		___________	
003	Upazila Condition(উপজেলার ধরন)	1. Intervention (ইন্টারভেনশন)<br>2. Control(কন্ট্রোল)	___	
004	Data Collector Name (তথ্য সংগ্রহকারীর নাম)		___________	
005	Participant ID Number(তথ্য প্রদানকারীর আইডি নম্বর)		___ ___ ___ ___	
006	Maternal Height Measurement (মায়ের উচ্চতার পরিমাপ)	Measure height using wall or door and record in meters (ঘরের দেয়াল অথবা দরজা ব্যবহার করে উচ্চতা পরিমাপ করুনএবং মিটারে রেকর্ড করুন)	___ .___ ___	
Household SES (খানার আর্থ-সামাজিক অবস্থা)				
100	What is the main source of drinking water for members of your household?(আপনার পরিবারের খাবার পানির প্রধান উৎস কি)	1. Own tube well (নিজস্ব টিউবওয়েল)<br>2. Others' tube well(অন্যের টিউবওয়েল)<br>3. Community tube well(সামাজিক টিউবওয়েল)<br>4. Supply water (piped) (পাইপে সরবরাহকৃত পানি)	___	

		5. Ring well/indara(রিংকুয়া/ইনদারা)<br>6. Pond(পুকুর<br>7. River/canal/ নদী/খাল)<br>8. Other(specify) (অন্যান্য নিদির্ষ্ট করূন):		
101	How long does it take to go fetch water from the source and return to the home? (ব্যবহারকৃত উৎস হতে পানি সংগ্রহ করে বাড়ীতে ফেরত আসতে কতক্ষন সময় ব্যয় হয়/লাগে)	[#minutes] [মিনিট]	___ ___ ___	
102	Who usually goes to this source to fetch water for the household? (আপনার পরিবার থেকে কে সাধারনত পানি আনতে যায়)	1. Adult female (পূর্ণ বয়স্ক মহিলা)<br>2. Adult male (পূর্ণ বয়স্ক পুরুষ)<br>3. Female child<15 years of age (১৫ বছরের কম বয়সী মেয়ে)<br>4. Male child (<15 years) (১৫ বছরের কমবয়সী ছেলে)<br>5. No specified person (নিদির্ষ্ট ব্যক্তি নয়)<br>6. Other (specify)অন্যান্য (নিদির্ষ্ট করুন)	____<br>Specify: (নিদির্ষ্ট করুন)<br>______________	
103	What is the main source of water in your household for: (আপনার খানার সদস্যরা নিম্নলিখিত কাজের জন্যকোন পানির উৎসের উপর নির্ভরশীল?)<br>a. Cooking? (রান্নার জন্য)<br>b. Bathing? (গোসলের জন্য)<br>c. Washing? (ধোয়ার জন্য)	1. Own tube well (নিজস্ব টিউবওয়েল)<br>2. Others' tube well (অন্যের টিউবওয়েল)<br>3. Community tube well (যৌথ টিউবওয়েল)<br>4. Supply water (piped) (পাইপে সরবারহকৃত পানি)<br>5. Ring well/indara (রিং-কুয়া/ইন্দারা)<br>6. Pond(পুকুর)<br>7. River/canal(নদ/খাল)<br>8. Other (specify): অন্যান্য(নিদির্ষ্ট করুন):	a. ____<br>b. ____<br>c. ____	
104	What kind of toilet facility do members of your household usually use?	1. Sanitary with flush (water sealed)	___	

	(আপনার খানার সদস্যরা সাধারনত কোন থরনের পায়খানা ব্যবহার করে?)	(ফ্লাসযুক্ত স্যানিটারী টয়লেট (ওয়াটার সীল)<br>2. Sanitary without flush (water sealed) [ফ্লাসবিহীন স্যানিটারী টয়লেট (ওয়াটার সীল)]<br>3. Pucca/pit without water seal [পাকা/পিট ল্যাট্রিন (ওয়াটার সীল ছাড়া)]<br>4. Kutcha/hanging (fixed place) (কাঁচা/ঝুরন্ত (নিদির্ষ্ট করুন)<br>5. Open field (খোলা মাঠ)<br>6. Other(specify)অন্যান্য (নিদির্ষ্ট করুন)	Specify: (নিদির্ষ্টকরুন)<br>__________	
105	Do you share this toilet facility with other households? (আপনি কি আপনার পায়খানা অন্য কোন পরিবারের সাথে যৌথ ভাবে ব্যবহার করেন?)	1. Yes(হ্যাঁ)<br>2. No(না)	____	
106	Do you own the house that you live in? /আপনি আপনার নিজের বাড়ীতে বসবাস করেন?	1. Yes(হ্যাঁ)<br>2. No(না)	____	
107	Does any member of this household own agricultural land? (আপনার খানার কোন সদস্যের চাষাবাদের জমির /আবাদি জমির মালিকানা রয়েছে কি?)	1. Yes(হ্যাঁ)<br>2. No (না)	____	If no, go to 109/যদি না তাহলে ১০৯ যেতে হবে
108	How many decimals of agricultural land do members of this household own? (Add total amount reported by all adult members of household? (আপনার খানার সদস্যরা সর্বমোট কত শতাংশ আবাদী জমির মালিক (পূর্ণ বয়স্ক সকল সদস্যের মালিকানা)	[If don’t know, enter 999]/ (জানা না থাকলে ৯৯৯ লিখুন)	___ ___ ___	
109	Do you have a garden or trees where you grow vegetables and/or fruit, or a pond where you fish? (আপনার কি কোন বাগান/ফলের গাছ বা পুকুর আছে যেখানে সবজি, ফল বা মাছ চাষ করা হয়?)	1. Yes (হ্যাঁ)<br>2. No(না)	____	

110	Does your household have electricity? (আপনার বাড়ীতে কি বিদ্যুৎ আছে?)	1. Yes(হ্যাঁ)<br>2. No(না)	___	If yes, go to 113 (যদি না হয় তাহলে১১৩ যেতে হবে)
111	Do you have any other kind of electric power? (আপনার কি অন্য কোন ধরনের বৈদ্যুতিক শক্তি আছে?)	1. Yes (হ্যাঁ)<br>2. No (না)	___	If no, go to 113(যদি না হয় তাহলে১১৩ যেতে হবে)
112	If yes, what type? (যদি হ্যাঁ হয় তাহলে কোন ধরনের?)	1. Generator (জেনারেটর)<br>2. Solar (সোলার)<br>3. Other (specify)অন্যান্য (নির্দিষ্ট করুন)	___	
113	What type of fuel does your household use for cooking? (আপনার বাড়ীতে রান্নার জন্য কি ধরনের জ্বালানী ব্যবহার করা হয়?)	1. Electricity (বিদ্যুৎ)<br>2. LPG (এলপিজি গ্যাস)<br>3. Natural gas (প্রাকিতিক গ্যাস)<br>4. Biogas (বায়ো গ্যাস)<br>5. Kerosene(কেরোসিন)<br>6. Charcoal (কাঠের কয়লা)<br>7. Wood/straw/leaves (কাঠ/খর/পাতা)<br>8. Animal dung (পশু পাখির বিষ্ঠা)<br>9. Other (specify)অন্যান্য (নির্দিষ্ট করুন)	___	
114	Main floor material (মেঝের প্রধান উপকরন কি?)	1. Concrete(কংক্রিট)<br>2. Brick/cement (ইট/সিমেন্ট)<br>3. Tin/ CI sheet (টিন/সি আই সীট)<br>4. Dirt(মাটি)<br>5. Other (specify): অন্যান্য (নির্দিষ্ট করুন)	___	
115	Main exterior wall material (বাহিরের দেয়ালের প্রধান উপকরন কি?)	1. Brick/cement (ইট/সিমেন্ট)<br>2. Wood(কাঠ)<br>3. Smoothed mud (মাটি)<br>4. Tile (টালি)<br>5. Tin (টিন)<br>6. Other (specify): অন্যান্য (নির্দিষ্ট করুন)	___	
116	Main roof material (ছাদের প্রধান উপকরন কি?)	1. Brick/cement (ইট/সিমেন্ট)<br>2. Jute stick (পাট কাঠি)<br>3. Bamboo/grass/stra	___	

		(বাশঁ/ঘাস/খড়)<br>4. Tin (টিন)<br>5. Other (specify):/অন্যান্য (নির্দিষ্ট করুন)		
	Household Assets: For the following items, please ask how many functional items of each type are present in the household and who owns them: (খানার সম্পদের পরিমান: নিন্মলিখিত কার্যকরী উপকরণ সমূহের জন্য জিজ্ঞাসা করুন?)	How many are present in household? (insert number) (কতগুলো কার্যকরী উপকরণ রয়েছে?)	১. Who owns this/thes eitem সেগুলোর মালিকানা কার?<br>২. You /আপনার নিজের<br>Your husband/ আপনার স্বামীর<br>৩. Jointly owned /যৌথ মালিকানা<br>৪. Someone else inhouseh old/ পরিবারের অন্যকারো	
117	1.Stove/gas burner (স্টোভ/গ্যাস বার্নার)	ক.-------	খ.----	
118	Bed/khat/chowki (খাট/চকি)	ক.-------	খ.----	
119	Table/chair (টেবিল/চেয়াল)	ক.-------	খ.----	
120	Electric fan (ceiling or floor/tamble) (বৈদ্যুতিক পাখাঃ সিলিং/মেঝে/টেবিল)	ক.-------	খ.----	
121	Table lamp (টেবিল ল্যাম্প)	ক.-------	খ.----	
122	Radio(রেডিও)	ক.-------	খ.----	
123	Television/টেলিভিশন)	ক.-------	খ.----	
124	Refrigerator (রেফ্রিজারেটর)	ক.-------	খ.----	
125	Microwave oven (মাইক্রোওয়েভ ওভেন)	ক.-------	খ.----	
126	Sewing machine/সেলাই মেশিন	ক.-------	খ.----	
127	Bicycle/rickshaw(বাইসাইকেল /রিক্সা)	ক.-------	খ.----	
128	Motorcycle(মটরসাইকেল)	ক.-------	খ.----	

129	Car(গাড়ী)	ক.-------	খ.----	
130	Mobile phone (মোবাইল ফোন)	ক.-------	খ.----	
131	Cow/ buffalo (গরু/মহিষ)	ক.-------	খ.----	
132	Goat/ sheep (ছাগল/ভেড়া)	ক.-------	খ.----	
133	Chicken/duck (মুরগী/হাঁস)	ক.-------	খ.----	
Exposure to SK and SS Home Visits – Validation (can be checked against register/red book entries) স্বাস্থ্যকর্মীর বাড়ী পরিদর্শনের তথ্য - যাচাই করুন (রেজিঃ ও লাল বই দেখে)				
200	Were you visited by the SK during the 7-8 th month of your pregnancy period? (আপনার গর্ভাবস্থায় ৭মথেকে ৮ম মাসের সময় স্বাস্থ্যকর্মী পরিদর্শন করেছিলেন?)	1. Yes(হ্যাঁ)<br>2. No(না)	___	
201	How many times did the SK visit you during the 7-8th month of your pregnancy period? (আপনার গর্ভাবস্থায় ৭ম থেকে ৮ম মাসের সময় স্বাস্থ্যকর্মী কতবার পরিদর্শন করেছিলেন কি ?)	[# visits during 7-8th month pregnancy] (পরিদর্শনের নম্বর)	___ ___	
202	After giving birth to your new baby, how old was he/she when the SK first visited you after the delivery? (সন্তান প্রসব করার পর প্রথম যখন স্বাস্থ্যকর্মী আপনাকে পরিদর্শন করেন তখন আপনার বাচ্চার বয়স কত ছিল?)	[# days of age] (বাচ্চার বয়স কতদিন ছিল)	___ ___	
203	From the first visit, until now, how many times after that did the SK visit you? (স্বাস্থ্যকর্মী প্রথম পরিদর্শন থেকে আজকের পূর্বে পযর্ন্ত আপনাকে মোট কতবার পরিদর্শন করেছেন?)	[# visits during pregnancy/following delivery] (কতবার পরিদর্শন হয়েছে গর্ভকালীন ও প্রসবপরবর্তি সময়ে)	___ ___	
204	Before today, when was the last time the SK visited you at home? (আজকের পূর্বে স্বাস্থ্যকর্মী কবে আপনাকে পরিদর্শন করেছেন?)	[# days – if more than 1 month, add 30 days for each month] (দিনে উল্লেখ করুন)	___ ___ ___	
Exposure to SS and SK Home Visits – Non-validated measures<br>স্বাস্থ্যসেবিকা ও স্বাস্থ্যকর্মীর বাড়ী পরিদর্শনের তথ্য -				
205	During the last home visit, how much time did she spend with you?	[# minutes] (মিনিট উল্লেখ করুন)	___ ___ ___	

	(শেষবার আপনাকে পরিদর্শনের সময় স্বাস্থ্যকর্মী আপনার সাথে কতসময় ব্যয় করেছিলেন?)			
206	At the last home visit, did she talk to you about breastfeeding, child feeding, or nutrition? (শেষবার পরিদর্শনের সময় স্বাস্থ্যকর্মী কি আপনাকে বুকের দুধ খাওয়ানো, বাচ্চার খাবার ও পুষ্টি নিয়ে কথা বলেছিলেন?)	1. Yes (হ্যাঁ)<br>2. No(না)	_____	
207	Did she talk about breastfeeding at her last visit? (শেষবার পরিদর্শনের সময় তিনি কি বুকের দুধ খাওয়ানো নিয়ে কথা বলেছিলেন?)	1. Yes (হ্যাঁ)<br>2. No (না)	_____	
208	Did she talk about complementary feeding at her last visit? (শেষবার পরিদর্শনের সময় তিনি কি শিশুর বাড়তি খাবার নিয়ে কথা বলেছিলেন?)	1. Yes (হ্যাঁ)<br>2. No(না)	___	
209	What advice did you receive from the SK on nutrition and feeding your child during her last visit? [Multiple responses possible; do NOT read responses to participant] (শেষবার পরিদর্শনে আপনি স্বাস্থ্যকর্মীর নিকট হতে শিশুর পুষ্টি ও খাদ্য নিয়ে কি ধরনের পরামর্শ পেয়েছিলেন? (একাধিক উত্তর প্রযোজ্যঃ উত্তর দাতার কাছে উত্তর বলবেন না)	1. Putting baby to breast immediately after birth (জন্মের সাথে সাথে বাচ্চাকে বুকে লাগানো)<br>2. Giving only colostrum (বাচ্চার মুখে শুধুমাত্র শাল দুধ দেওয়া)<br>3. No pre- or post lacteals (honey/ mustard oil/ glucose water) (বুকের দুধ ছাড়া অন্য কোন তরল খাবার না খাওয়ানো) মধু/সরষের তেল/চিনির পানি)<br>4. Feed only breast milk up to 6 months (৬মাস পযর্ন্ত শুধু মাত্র বুকের দুধ খাওয়ানো)<br>5. Positioning & attachment for breast feeding (বুকের দুধ খাওয়ানোর	___<br>___<br>___<br>___<br>___	

		সংযোগ ও অবস্থান সংযোগ সম্পর্কে বলা)<br>6. Feeding mashed family food after 6 months (৬ মাস পর নরম করে বাড়তি/পারিবারিক খাবার খাওয়ানো)<br>7. Feeding animal source foods (প্রানিজ উৎসের খাবার খাওয়ানো)<br>8. Cooking/adding with oil (রান্নার তেল যোগ করা)<br>9. Adding MNP to child's food (বাচ্চার খাবারে পুষ্টিকনা মেশানো)<br>10. Washing hands with water and soap before prep/feeding child (খাদ্য তৈরীর আগে ও শিশুকে খাওয়ানোর আগে সাবান ও পানি দিয়ে হাত ধোয়া)<br>11. Feeding during illness/ extra after illness (অসুস্থ্য অবস্থায় শিশুকে খাওয়ানো/সুস্থ্য হওয়ার পর বেশী করে খাওয়ানো)<br>12. Other (specify) অন্যান্য: (নির্দিষ্ট করুন)<br>13. Don't know (জানি না)		
210	During the last home visit, how long did she spend talking with you about only nutrition and child feeding? (শেষবার পরিদর্শনের সময় স্বাস্থ্যকর্মী পুষ্টি বা বাচ্চার খাবার নিয়ে কতসময় ব্যয় করেছিলেন?)	[# minutes] (মিনিটে উল্লেখ করুন)	___ ___ ___	
211	The last time you saw the SK; did she give any advice to you about your own health? (শেষবার পরিদর্শনের সময় যখন স্বাস্থ্যকর্মী এসেছিলেন তখন কি	1. Yes (হ্যাঁ)<br>2. No (না)	_____	If 2 (No), go to 213. যদি না (২) হয় তবে ২১৩ যেতে হবে

	তিনি আপনার স্বাস্থ্য সম্পর্কিত কোন পরামর্শ দিয়েছিলেন?)			
212	In the last six months, what kinds of health information/services did you receive from the SK?/ [Multiple responses possible; do NOT read list to participant]<br>গত ৬ মাসে স্বাস্থ্যকর্মী কি ধরনের তথ্য/সেবা দিয়েছিল?<br>(একাধিক উত্তর প্রযোজ্য: উত্তর দাতার কাছে উত্তর বলবেন না)	1. Received advice about family planning (পরিবার পরিকল্পনা সম্পর্কিত পরামর্শ)<br>2. Received advice about pregnancy (গর্ভ সংক্রান্ত তথ্য পরামর্শ)<br>3. Took weight when pregnant (গর্ভ কালীন ওজন নেয়া)<br>4. Checked blood pressure (রক্তচাপ পরীক্ষা করা)<br>5. Tested for pregnancy (গর্ভ পরীক্ষা করা)<br>6. Told me about maternal nutrition (গর্ভকালীন পুষ্টি সম্পর্কে বলা)<br>7. Told me about EIBF after delivery (জন্মের পর যত তারাতারি সম্ভব বুকের দুধ খাওয়ানো সম্পর্কে বলা)<br>8. Told me about EBF for 6 months (৬ মাস পযর্ন্ত শুধুমাত্র বুকের দুধ খাওয়ানো)<br>9. Mother should eat well (মায়ের পুষ্টিকর খাবার খাওয়া)<br>10. Received no service in the last six months (গত ৬মাসে কোন সেবা গ্রহন করেনি?)<br>11. Received advice to take IFA and Caltium tablet	____<br>____<br>____<br>____<br>____<br>____<br>(Specify):/(নির্দিষ্ট করুন)<br>________________	

		evereday (প্রতিদিন আয়রন ও ক্যালসিয়াম ট্যাবলেট খেতে বলা)<br>12. Take rest for 2 hours/day (দিন কমপক্ষে ২ ঘন্টা বিশ্রাম নিতে বলা)<br>13. Did not let you lift heavy work load during pregnancy (গর্ভকালীন সময়ে ভারী কাজ করতে নিরুৎসাহিত করা)<br>14. Other (specify) অন্যান্য (নির্দিষ্ট করুন)<br>99. Don't know (জানি না)		
213	Have you ever attended a health education forum (Shasto Forum) organized by SK? (স্বাস্থ্যকর্মীর কোন স্বাস্থ্য শিক্ষা বিষয়ক সভায় অংশগ্রহন করেছেন কি?)	1. Yes (হ্যাঁ)<br>2. No (না)	___	If 2 (No), go to 217.<br>যদি না (২) হয়, তবে ২১৭ যেতে হবে
214	Have you attended a health education forum within the past 6 months organized by SK? (গত ৬মাস স্বাস্থ্যকর্মী আয়োজিত কোন স্বাস্থ্যশিক্ষা বিষয়ক সভায় অংশ গ্রহন করেছেন কি?)	1. Yes (হ্যাঁ)<br>2. No (না)	___	
215	During your last Health Forum with the SK, how much time did you spend there? (আপনি সর্বশেষ স্বাস্থ্যকর্মীর স্বাস্থ্য শিক্ষা বিষয়ক সভায় কতসময় অংশ গ্রহন করেছেন?)	[# minutes]<br>(মিনিট উল্লেখ করুন)	___ ___ ___	
216	Last time you attended a health forum, what did the SK talk about? [Multiple responses possible; do NOT read responses to participant] (সর্বশেষ স্বাস্থ্যকর্মীর স্বাস্থ্য শিক্ষা বিষয়ক সভায় কি নিয়ে আলোচনা হয়েছিল?)	1. Advice on family planning (পরিবার পরিকল্পনা সম্পর্কিত পরামর্শ)<br>2. Advice on water and sanitation (পানি ও পয়ঃ নিস্কাশন বিষয়ক পরামর্শ))<br>3. Immunization (শিশুর টিকা)	___ ___<br>___ ___<br>___ ___<br>___ ___	

		4. Maternal diet (5 types of food) গর্ভবতির খাবার (৫ ধরনের খাবার)<br>5. Taking IFA and calcium tablets (আয়রন ও ক্যালসিয়াম ট্যাবলেট সম্পর্কে আলোচনা)<br>6. Advice on TB (যক্ষা সংক্রান্ত পরামর্শ)<br>7. Advice on breastfeeding (বুকের দুধ সম্পর্কে পরামর্শ)<br>8. Advice on complementary feeding (শিশুর বাড়তি খাবার সম্পর্কে পরামর্শ)<br>9. Adding MNP (শিশুর খাবারে পুষ্টিকনা যোগ করা)<br>10. Washing hands with water and soap before prep/feeding child (শিশুকে খাবার খাওয়ানোর আগে ও পরে সাবান ও পানি দিয়ে হাত পরিস্কার করা)<br>11. Encouraging child to eat enough (শিশুকে খাবার খেতে উৎসাহ দেওয়া)<br>12. Feeding during illness/extra after illness (অসুস্থ্য অবস্থায় শিশুকে খাওয়ানো/ সুস্থ্য হওয়ার পর বেশি করে খাবার খাওয়ানো)<br>13. Other(specify) অন্যান্য (নির্দিষ্ট করুন)<br>99. Don't know (জানি না)		
217	Were you visited by the SSduring the 7-8 month of	1. Yes (হ্যাঁ)	___	

	your pregnancy period?<br>(আপনার গর্ভাবস্থায় ৭ম থেকে ৮ম মাসের সময় স্বাস্থ্যসেবিকা পরিদর্শন করেছিলেন?)	2. No (না)		
218	How many times did the SS visit you during the 7-8th month of your pregnancy period?<br>(আপনার গর্ভাবস্থায় ৭ম থেকে ৮ম মাসের সময় স্বাস্থ্য সেবিকা আপনাকে কতবার পরিদর্শন করেছিলেন?)	[# visits during 7-8th month pregnancy]<br>(৭ম থেকে ৮ম মাসে কতবার ভিজিট করেছে?)	___ ___	
219	After giving birth to your new baby, how old was he/she when the SS first visited you after the delivery?<br>(বাচ্চা/সন্তান প্রসব করার পর প্রথম যখন স্বাস্থ্য সেবিকা আপনাকে পরিদর্শন করেছিলেন তখন আপনার বাচ্চার বয়স কতছিল?)	[# days of age]<br>(কত দিন বয়স)	___ ___	
220	From the first visit, until now, how many times after that did the SS visit you?<br>(স্বাস্থ্যসেবিকা প্রথম পরিদর্শন থেকে বর্তমান সময় পযর্ন্ত মোট কতবার আপনাকে পরিদর্শন করেছিলেন?)	[# visits during pregnancy/following delivery]<br>(গর্ভাবস্থা থেকে প্রসব পরবর্তি সময় পযর্ন্ত)	___ ___	
221	Before today, when was the last time the SS visited you at home?<br>(আজকের পূর্বে স্বাস্থ্যসেবিকা কবে আপনাকে পরিদর্শন করেছিলেন?)	[# days – if more than 1 month, add 30 days for each month]<br>(দিন- ১মাসের উপরে হলে প্রতিমাসে ৩০ দিন হিসেবে ধরতে হবে)	___ ___ ___	
222	During the last home visit, how much time did she spend with you?<br>(শেষবার আপনাকে পরিদর্শনের সময় স্বাস্থ্যসেবিকা আপনার সাথে কতক্ষন/কতসময় ব্যয় করেছিলেন?)	[# minutes]/<br>(মিনিটে উল্লেখ করুন)	___ ___ ___	
223	At the last home visit, did she talk to you about breastfeeding, child feeding, or nutrition?<br>(শেষবার পরিদর্শনের সময় স্বাস্থ্যসেবিকা কি আপনাকে বুকের দুধ খাওয়ানো, বাচ্চার খাবার ও পুষ্টি নিয়ে কথা বলেছিলেন?)	1. Yes(হ্যাঁ)<br>2. No(না)	___	

224	Did she talk about breastfeeding at her last visit? (শেষবার পরিদর্শনের সময় তিনি কি বুকের দুধ খাওয়ানো নিয়ে কথা বলেছিলেন?)	1. Yes (হ্যাঁ)<br>2. No (না)	___	
225	Did she talk about complementary feeding at her last visit? (শেষবার পরিদর্শনের সময় তিনি কি শিশুকে বাড়তি খাবার নিয়ে কথা বলেছিলেন?)	1. Yes (হ্যাঁ)<br>2. No (না)	___	
226	What advice did you receive from the SS on nutrition and feeding your child during her last visit? [Multiple responses possible; do NOT read responses to participant] শেষবার পরিদর্শনে আপনি স্বাস্থ্যসেবিকার কাছ থেকে শিশুর পুষ্টি ও খাদ্য নিয়ে কি ধরনের পরামর্শ পেয়েছেন?<br>(একাধিক উত্তর প্রযোজ্য: উত্তর দাতার কাছে উত্তর বলবেন না)	1. Putting baby to breast immediately after birth (জন্মের সাথে সাথে বাচ্চাকে বুকে লাগানো)<br>2. Giving only colostrum (বাচ্চার মুখে শাল দুধ দেওয়া)<br>3. No pre- or post lacteals (honey/mustard oil/glucose water) (বুকের দুধ ছাড়া অন্য কোন তরল খাবার খাওয়ানো যাবে না (মধু/ সরষের তেল/ চিনির পানি)<br>4. Feed only breast milk up to 6 months (৬মাস পযর্ন্ত শুধুমাত্র বুকের দুধ খাওয়ানো)<br>5. Positioning & attachment for breastfeeding (বুকের দুধ খাওয়ানোর সংযোগ ও অবস্থান সংযোগ সম্পর্কে বলা)<br>6. Feeding mashed family food after 6 months (৬ মাস পর নরম করে বাড়তি/ পারিবারিক খাবার খাওয়ানো)<br>7. Feeding animal source foods	___<br>___<br>___<br>___<br>___<br>___<br>___<br>___<br>___	

		(প্রানিজ উৎসের খাবার খাওয়ানো)<br>8. Cooking/adding with oil (রান্নায় তেল যোগ করা)<br>9. Adding MNP to child's food (বাচ্চার খাবারে পুষ্টিকনা মেশানো)<br>10. Washing hands with water and soap before prep/ feeding child (খাদ্য তৈরীর আগে ও শিশুকে খাওয়ানোর আগে সাবান ও পানি দিয়ে হাত ধোয়া)<br>11. Feeding during illness/extra after illness (অসুস্থ্য অবস্থায় শিশুকে খাওয়ানো/ সুস্থ্য হওয়ার পরে বেশি করে খাওয়ানো)<br>12. Other (specify) (অন্যান্যঃ নির্দিষ্ট করুন)<br>99. Don't know (জানি না)	___<br>___<br>___<br>___<br>___	
227	During the last home visit, how long did she spend talking with you about only nutrition and child feeding? (শেষবার পরিদর্শনের সময় তিনি পুষ্টি ও বাচ্চার খাবার নিয়ে কতক্ষন/কতসময় ব্যয় করেছিলেন?)	[# minutes]<br>(মিনিটে উল্লেখ করুন)	___ ___ ___	
228	The last time you saw the SS, did you purchase any kind of medicine or health items from her? (শেষবার পরিদর্শনের সময় আপনি কি স্বাস্থ্য সেবিকার কাছ থেকে কোন স্বাস্থ্য সামগ্রী / ওষুদ ক্রয় করেছিলেন?)	1. Yes (হ্যাঁ)<br>2. No (না)	___	If 2 (No), go to 231/যদি না(২)হয়, তবে ২৩১ যেতে হবে
229	What kind of medicine or health items did you purchase from the SS? [Multiple responses possible; do NOT read list to participant] (আপনি কি ধরনের স্বস্থ্য সামগ্রী /ওষুধ ক্রয় করেছিলেন?)	1. IFA (আয়রন ট্যাবলেট)<br>2. Calcium (ক্যালসিয়াম ট্যাবলেট)<br>3. Paracetamol (প্যারাসিটামল)<br>4. Vitamin (ভিটামিন)<br>5. Anti-Histamine (এন্টি হিষ্টাসিন)	___<br>___<br>___<br>___	

		6. Family planning material (পরিবার পরিকল্পনা সামগ্রী)<br>7. Soap (সাবান)<br>8. Sanitary Napkin (স্যানিটারী ন্যাপকিন)<br>9. Delivery kits (ডেলিভারী কীট)<br>10. Salt (with iodine) (আয়োডিন যুক্ত লবন)<br>11. Syrup (সিরাপ)<br>12. Pushtikona (পুষ্টিকনা)<br>13. Monimix (মনিমিক্স)<br>14. MyMix (মাই মিক্স)<br>15. ORS (ওর স্যালাইন)<br>14. Didn't buy anything in last month (শেষ মাসে কোন ক্রয় করে নি)<br>15. Other (specify) অন্যান্য (নির্দিস্ট করুন)<br>99. Don't know (জানি না)	___<br>___<br>(Specify): __________<br>__________	
230	How much in total did you pay when you bought medicine and health items from SS/PS during her last visit??<br>(সর্বশেষ বার স্বাস্থ্য সেবিকা/পিএস এর কাছ থেকে স্বাস্থ্য সামগ্রী/ ওষুধ ক্রয় করার সময় কত টাকা ব্যয় করেছিলেন)	[Enter amount in Taka]<br>(টাকার পরিমান উল্লেখ করুন)	__ __ __ __ Tk.	
231	During her last visit, did you receive any advice from the SS on Pushtikona?<br>[Show the Pushtikona packet] (স্বাস্থ্য সেবিকার শেষ পরিদর্শনে আপনি পুষ্টিকনা সম্পর্কে কোন পরামর্শ পেয়েছিলেন কিনা?)	1. Yes (হ্যাঁ)<br>2. No (না)	___	If 2 (No), go to 300/যদি না(২)হয়, তবে ৩০০ যেতে হবে
232	During the last purchase, how many packets of		___ ___ ___	

	Pustikona did you buy?<br>(সর্বশেষ পরিদর্শনে কত প্যাকেট পুষ্টিকনা ক্রয় করেছিলেন?)	[# packets]<br>(কত প্যাকেট উল্লেখ করুন)		
Obstetric History & ANC Course				
300	Number of prior pregnancies<br>(কততম গর্ভ)	Number of pregnancies (Will be 01 if first pregnancy; include miscarriages)<br>(বর্তমান প্রসব সহ মোট গর্ভের সংখ্যা)	____ ____	
301	Number of prior miscarriages<br>(পূর্বের গর্ভ পাতের সংখ্যা)		____ ____	
302	Number of prior preterm deliveries (পূর্বের গর্ভের অপরিনত শিশু জন্মের সংখ্যা)	Delivery ≤8 months' gestation (<2500 kg)৮মাসের আগে প্রসব হলে (২৫০০গ্রাম এর নিচে হলে)	____ ____	
303	Number of prior term deliveries<br>(পূর্বের গর্ভের পরিনত শিশু জন্মের সংখ্যা)	Delivery 9 months or later gestation (≥2500 kg)<br>(৯মাস পূর্ণ অথবা ২৫০০গ্রামের উপরে)	____ ____	
304	Number of living children (includes child from this most recent pregnancy)<br>(পরিনত /জীবিত শিশুর সংখ্যা (বর্তমান শিশুটি সহ)		____ ____	
305	Have you ever been told ***before*** you were pregnant that you have any of the following conditions:<br>(গর্ভবতী হওয়ার পূর্বে আপনার কোন শারীরিক সমস্যা ছিল কি?)<br>a. Diabetes(ডায়াবেটিস)<br>b. High blood pressure (উচ্চ রক্ত চাপ)<br>c. when not pregnant/ Malnutrition – too (অতিরিক্ত-অপুষ্টি)<br>d. under-weight (কম ওজন)<br>e. Too short (অতিরিক্ত খাটো)<br>f. Asthma (হাঁপানি/শ্বাস কষ্ট)<br>g. Tuberculosis (যক্ষা)	1=Yes (হ্যাঁ)<br>2=No (না)	a. ___<br>b. ___<br>c. ___<br>d. ___<br>e. ___<br>f. ___<br>g. ___<br>h. ___<br>i. ___	

	h. Anemia, pallor (রক্ত স্বল্পতা)<br>i. Heart condition /rheumatic heart disease (হার্টের সমস্যা)<br>j. Kidney disease (কিডনীর সমস্যা)<br>k. Other (specify): অন্যান্য (নির্দিষ্ট করুন)		j. ___<br>k. ___<br><br>Specify:<br>____________	
306	Do you have birth planning during your last pregnancy?<br>(শেষ গর্ভাবস্থায় কোন আপনার প্রসব পরিকল্পনা ছিল কি?)	1. Yes/হ্যাঁ<br>2. No/না	___	If 2 (No), go to 308/যদি না(২)হয়, তবে ৩০৮ যেতে হবে
307	Where did you plan to go for delivery?(প্রসবের পরিকল্পনা কোথায় ছিল?)	1. Home(বাড়ীতে)<br>2. Hospital (হাসপাতালে)<br>3. Other(specify)অন্যান্য (নির্দিষ্ট করুন)	___<br>Specify:<br>____________	
308	Where did your delivery occur?(কোথায় প্রসব হয়েছিল?)	1. Home(বাড়ীতে)<br>2. Hospital (হাসপাতালে)<br>3. In vehicle on way to hospital (হাসপাতালে নেয়ার পথে)<br>4. Other (specify) অন্যান্য (উল্লেখ করুন)	___<br>Specify:<br>____________	
309	What date did you deliver?<br>(প্রসবের তারিখ)	Day/month/year Gregorian; for single digit day or month, write 0 first (ex: July=07)<br>(দিন/মাস/বছর)	___ ___/___<br>___/ ___ ___	
310	What type of delivery did you have?<br>(কি ধরনের প্রসব হয়েছিল?)	1. Normal vaginal(নরমাল)<br>2. Cesarean section(সিজার)<br>3. Assisted vaginal (vacuum) (ভেকুম)	___	If 1 or 3, go to 312.
311	If Cesarean, what was the reason?(কেন সিজার করেছিলেন?)	1. Medical/problem (মেডিকেল সমস্যা ছিল)<br>2. Familychoice/preference (পরিবারের ইচ্ছা ছিল)<br>3. Doctor recommended (ডাক্তারের পরামর্শ ছিল?)<br>4. Other (specify)	___<br>Specify:<br>____________	

		অন্যান্য (নির্দিষ্ট করুন)		
312	Have you received/are you using contraception postpartum?<br>(এখন কোন পরিবার পরিকল্পনা পদ্বতি ব্যবহার করছেন কি?)	১. Yes(হ্যাঁ)<br>2. No(না)	___	If 2 (No), go to 314.(যদি না হয় তাহলে৩১৪ যেতে হবে)
313	Which method are you using?<br>(কোন পদ্বতি ব্যবহার করছেন?)	1. Male condoms(কনডম)<br>2. Oral contraceptive pills (খাবার বড়ি)<br>3. DepoProvera Injection (ইনজেকশন)<br>4. Implant (Norplant) (ইমপ্লান্ট)<br>5. IUD(আই ইউ ডি)<br>6. Other (specify): অন্যান্য(নির্দিষ্ট করুন)	___<br>Specify: ___________	
314	Are you planning to use contraception postpartum?<br>(আপনার কোন পরিবার পরিকল্পনা পদ্বতি ব্যবহারের পরিকল্পনা আছে কি?)	1. Yes(হ্যাঁ)<br>2. No(না)	___	If 2 (No), go to 316/যদি না(২)হয়, তবে ৩১৬ যেতে হবে
315	What method do you plan to use?<br>(কোন পদ্বতি ব্যবহারের পরিকল্পনা আছে?	1. Male condoms/কনডম<br>2. Oral contraceptive pills/খাবার বড়ি<br>3. DepoProvera Injection/ইনজেকশন<br>4. Implant (Norplant)/ ইমপ্যান্ট<br>৫. IUD/আই ইউ ডি<br>6. Other (specify):/অন্যান্য (নির্দিষ্টকরুন)	___<br>Specify: ___________	
316	At birth or soon after, did you receive a dose of Vitamin A? (বাচ্চা হওয়ার সময় অথবা বাচ্চা হওয়ার পরে ভিটামিন এ খেয়েছিলেন কিনা?)	1. Yes/হ্যাঁ<br>2. No/না	___	

Pregnancy Complication Validation<br>(গর্ভকালীন জটিলতা যাচাই করুন)				
400	Did you have any complications during your pregnancy, that is, ***before***you went into labor? (গর্ভাবস্থায় আপনার কি কোন ধরনের জটিলতা ছিল?)	1. Yes (হ্যাঁ)<br>2. No (না)	___	
401	Which complications did you have: কি ধরনের জটিলতা ছিল?<br>a. Anemia / রক্ত স্বল্পতা<br>b. Severe vomiting/ অতিরিক্ত বমি বমি ভাব<br>c. Severe edema/ হাতেপায়ে পানি আসা<br>d. High blood pressure/উচ্চ রক্তচাপ<br>e. Vaginal bleeding জরায়ুর মুখ দিয়ে রক্ত আসা<br>f. Pre eclampsia/ খিচুনি<br>g. Diabetes/ ডায়াবেটিস<br>h. High fever/ ভীষন জ্বর<br>i. preterm labor/ আগে থেকে ব্যথা<br>j. Low weight gain/ কম ওজন<br>k. Leakage of fluid/preterm rupture of membranes/সময়ের আগে পানি ভাঙ্গা<br>l. Back pain/কোমর ব্যথা<br>m. Other (specify):/ অন্যান্য(নির্দিষ্ট করুন)	1=Yes (হ্যাঁ)<br>2=No (না)	a. ___<br>b. ___<br>c. ___<br>d. ___<br>e. ___<br>f. ___<br>g. ___<br>h. ___<br>i. ___<br>j. ___<br>k. ___<br>l. ___<br>m.<br>Specify:<br>___________	
402	Did you go to the hospital for these complication?/আপনার এই জটিলতার জন্য হাসপাতালে গিয়েছিলেন কি?	1. Yes/হ্যাঁ<br>2. No/না	___	If 2 (No), go to 404/যদি না(২)হয়, তবে ৪০৪ যেতে হবে
403	What treatment did you receive from hospital?/ হাসপাতাল থেকে আপনি কি ধরনের চিকিৎসা নিয়েছিলেন?			

	a. Medicine- ঔষুধ<br>b. Blood transfusion- রক্ত গ্রহণ<br>c. Other (specify):/ অন্যান্য (নিদির্ষ্ট করুন)	a. 1=Yes<br>b. 1=Yes<br>c. 1=Yes	a. 2=No<br>b. 2=No<br>c. 2=No	a. ___<br>b. ___<br>c. ___	
404	When did your labor start? কখন আপনার প্রসব ব্যথা উঠেছিল?	1. Early morning (4-7 AM) খুব সকালে(৪-৭)<br>2. Mid-morning (7-9 AM) সকালে (৭-৯)<br>3. Late morning (9AM – 12 PM)সকাল(৯-১২)<br>4. Early afternoon (12 – 3 PM)দুপুর ১২-৩)<br>5. Midafternoon (3-6 PM) দুপুর(৩-৬)<br>6. Evening (6-9 PM) সন্ধ্যা (৬-৯)<br>7. Night (9 PM – 12 AM) রাত(৯-১২)<br>8. Late night (1 – 4 AM) রাত(১-৪)<br>9. Not applicable (specify): (নিদির্ষ্টকরুন)		___	Please validate the question against time since sunrise/set. If (9) go to 406 যদি ৯ হয়, তবে ৪০৬এ যেতে হবে
405	For how many hours did you experience labor? (ব্যথা উঠার কত ঘন্টা পর প্রসব হয়েছিল)	Ask how much time in hours passed, using daylight if needed, between answer above and delivery. (ঘন্টায় লিখুন)		___ ___	
406	Did you have any complications ***during labor and delivery***? (প্রসবের সময় কোন জটিলতা ছিল কি?)	1. Yes/হ্যাঁ<br>2. No/না		___	If 2 (No), go to 410/যদি না(২)হয়, তবে ৪১০ যেতে হবে
407	Which complications did you have: / কি ধরনের জটিলতা ছিল?<br>a. Pre-eclampsia (প্রি-এক লামশিয়া)<br>b. Eclampsia/convulsions (একলামশিয়া)<br>c. Heavy bleeding during labor (প্রসবের সময় প্রচন্ড রক্ত ক্ষরন)	1=Yes (হ্যাঁ)<br>2=No (না)		a. ___<br>b. ___<br>c. ___<br>d. ___<br>e. ___	

	d. Heavy bleeding after delivery<br>(প্রসবের পর প্রচন্ড রক্ত ক্ষরন)<br>e. Retained placenta<br>গর্ভফুল আটকে থাকা<br>f. Prolonged labor/<br>দীর্ঘায়িত আটকে ব্যথা<br>g. Obstructed labor<br>বাধাগ্রস্থ ব্যথা<br>h. Fever/infections<br>জ্বর/ইনফেকশন<br>i. Breech/non-cephalic presentation<br>বচ্চার মাথা উপরের দিকে<br>j. Fever/infection in labor<br>জ্বর/ প্রসবের সময় ইনফেকশন<br>k. Other (specify):<br>.অন্যান্য(নিদির্ষ্ট করুন)			f. ___<br>g. ___<br>h. ___<br>i. ___<br>j. ___<br>k. ___<br>Specify:<br>__________	
408	Did you go to the hospital for these complications?<br>আপনি কি এই সমস্যার জন্য হাসপাতালে গিয়েছিলেন?	1. Yes/হ্যাঁ<br>2. No/না		___	If 2 (No), go to 410/যদি না (২) হয়, তবে ৪১০ যেতে হবে
409	What treatment did you receive from hospital?<br>হাসপাতালে কি ধরনের সেবা নিয়েছিলেন?<br>a. Medicine/ ঔষুধ<br>b. Blood transfusion/রক্ত গ্রহণ<br>c. Other (specify):<br>অন্যান্য(নিদির্ষ্ট করুন)	a. 1=Yes<br>b. 1=Yes<br>c. 1= Yes	a. 2=No<br>b. 2=No<br>c. 2=No	___<br>___<br>___	
410	Did you have any complications after the delivery? প্রসবের পর আপনার কোন ধরনের জটিলতা ছিল কি?	1. Yes/হ্যাঁ<br>2. No/না		___	If 2 (No), go to 414/যদি না(২)হয়, তবে ৪১৪ যেতে হবে
411	Which complications did you have: কি ধরনের জটিলতা ছিল?<br>a. Eclampsia/convulsion (খিচুনী)<br>b. Heavy bleeding after delivery<br>(প্রসবের পর প্রচন্ড রক্ত ক্ষরন)	1=Yes (হ্যাঁ)<br>2=No (না)		a. ___<br>b. ___<br>c. ___	

	c. Fever(জ্বর)<br>d. severe lower abdominal pain (তল পেটে প্রচন্ড ব্যথা)<br>e. infection (ইনফেকশন)<br>f. severe weakness অতিরিক্ত শারিরীক দুর্বলতা<br>g. Other (specify):/ অন্যান্য(নির্দিষ্ট করুন)			d. ___<br>e. ___<br>f. ___<br>g. ___<br>___<br>Specify	
412	Did you go to the hospital for these complications? (এই সমস্যার জন্য হাসপাতালে গিয়েছিলেন কি?)	1. Yes/হ্যাঁ<br>2. No/না		___	If no, go to 414./না হলে ৪১৪ যেতে হবে
413	What treatment did you receive from hospital? কি ধরনের সেবা নিয়েছিলেন?<br>a. Medicine/ ঔষুধ<br>b. Blood transfusion/ রক্ত গ্রহণ<br>c. Other (specify): অন্যান্য(নির্দিষ্ট করুন)	a. 1=Yes<br>b. 1=Yes<br>c. 1=Yes	a. 2=No<br>b. 2=No<br>c. 2=No	__<br>__<br>__	
414	Did your child face any complication after birth? (জন্মের পর আপনার শিশু কোন জটিলতা ছিল কি?)	1. Yes/হ্যাঁ<br>2. No/না		___	If no, go to 500/ না হলে ৫০০ যেতে হবে
415	What complication did your child face after birth? (জন্মের পর আপনার শিশু কোন ধরনের জটিলতায় পরেছিল?)<br>a. Child not sucking well/শিশু ঠিকমত বুকের দুধটানতে না পারা<br>b. Vomiting/diarrhea /বমি/ ডায়রিয়া<br>c. Umbilical cord infection /নাভীতে ইনফেকশন<br>d. Skin rash/infection/ত্বকের সমস্যা<br>e. Convulsion/খিচুনী<br>f. Lethargic or not respond	1=Yes (হ্যাঁ)<br>2=No (না)		a. ___<br>b. ___<br>c. ___<br>d. ___<br>e. ___<br>f. ___	

	properly/ঠিকমত নড়াচড়া না করা<br>g. Redness of eye or discharge/চোখ লাল হয়ে যাওয়া<br>h. Fever or hypothermia/অধিক তাপমাত্রা<br>i. Rapid Breathing /ঘন ঘন শ্বাস নেওয়া<br>j. Jaundice/জন্ডিস<br>k. Other (specify):/অন্যান্য(নিদির্ষ্ট করুন)		g. ___<br>h. ___<br>i. ___<br>j. ___<br>k. ___<br>Specify__________	
Current Breastfeeding Practices (বর্তমানে বুকের দুধ খাওয়ানোর তথ্য)				
500	How long after birth did you first put your baby to the breast? (জন্মের কতক্ষন পর শিশুকে বুকের দুধ খাওয়ানো হয়েছিল?)	[# hours]<br>[# minutes] (if more than 59 minutes, divide into hours and minutes) (ঘন্টা/মিনিটে উল্লেখ করুন)	___ hours/ঘন্টা<br>___ minutes/ মিনিট	
501	Did anyone help you put the baby to the breast after delivery? (বাচ্চাকে বুকের দুধ খাওয়ানো সময় কেউ আপনাকে সাহায্য করেছিল?)	1. Yes/হ্যাঁ<br>2. No/না	___	
502	Who helped you put the baby to the breast after delivery?<br>(বাচ্চাকে বুকের দুধ খা ওয়ানোর সময় কে আপনাকে সাহায্য করেছিল?)	1. Hospital/Upazila Health Center, Clinic /হাসপাতাল/উপজেলা স্বাস্থ্য কেন্দ্র/ক্লিনিক<br>2. Doctor /ডাক্তার<br>3. Midwife/nurse /ধাত্রী/নার্স<br>4. Govt. Heath Worker (FWA/HA)/সরকারী কর্মী<br>5. BRAC SS /ব্র্যাক স্বাস্থ্য সেবিকা<br>6. BRAC SK /ব্র্যাক স্বাস্থ্যকর্মী<br>7. PushtiKormi/পুষ্টিকর্মী<br>8. Others NGO Worker /অন্যান্য-এনজিও কর্মী<br>9. TTBA /প্রশিক্ষন প্রাপ্ত ধাত্রী	___ ___	

		10. TBA/ধাত্রী<br>11. Village Doctor /গ্রাম্য ডাক্তার<br>12. Homeopath Doctor /হোমিওপ্যাথিক ডাক্তার<br>13. Kabiraj/herbal healer/কবিরাজ<br>14. Spiritual healer/সর্পিল বৈদ্য<br>15. Husband /স্বামী<br>16. Mother/Mother-in-Law / মা/শাশুড়ী<br>17. Any other family member /পরিবারের অন্য কোন সদস্য<br>18. Relative/Friends /আত্নীয়/বন্ধু<br>19. Private clinic staff/বেসরকারী ক্লিনিকের স্টাফ<br>20. Community clinic staff/কমিউনিটি ক্লিনিকের স্টাফ<br>21. EPI center staff/টীকা কেন্দ্রের স্টাফ<br>22. Myself /নিজেই<br>23. Nobody/Never need advice /কেউ না/কখনো কেউ উপদেশ দেয়নি<br>24. Others (specify)/অন্যান্য (নির্দিষ্ট করুন)		
503	Was the baby put to the breast before or after someone cleaned the baby? /শিশুকে কখন মায়ের বুকে লাগানো হয়েছিল (পরিষ্কারের আগে /পরে)	1. Before someone cleaned the baby/শিশুকে পরিষ্কারের আগে<br>2. After someone cleaned the baby/শিশুকে পরিষ্কারের পরে<br>3. Don't know/remember/মনে নেই/জানা নেই	___	
504	Was the baby put to the breast before or after someone cleaned you? /	1. Before someone cleaned me/আপনাকে পরিষ্কারের আগে	___	

	কখন শিশুকে আপনার বুকে লাগিয়েছিল (পরিষ্কারের আগে/পরে)	2. After someone cleaned me (আপনাকে পরিষ্কারের পরে)<br>3. Don't know/remember/মনে নেই/জানি না		
505	Did you or anyone else check to see if the baby was suckling well? / আপনার শিশু ঠিকমত বুকের দুধ টানতে পারছে কিনা তা আপনি অথবা অন্য কেউ দেখে নিশ্চিত হয়েছিল কি?	1. Yes/হ্যাঁ<br>2. No/না	___	
506	Who checked if the baby was suckling well? /আপনার শিশু বুকের দুধ ঠিকমত টানতে পারছে কিনা তা কে পরীক্ষা করেছিলেন?	1. Hospital/Upazila Health Center, Clinic (হাসপাতাল/উপজেলা স্বাস্থ্যকেন্দ্র/ক্লিনিক)<br>2. Doctor /ডাক্তার<br>3. Midwife/nurse /ধাত্রী/নার্স<br>4. Govt. Heath Worker (FWA/HA) /সরকারী স্বাস্থ্যকর্মী(পরিবার কল্যান সহকারী , স্বাস্থ্য সহকারী)<br>5. BRAC SS /ব্র্যাক স্বাস্থ্য সেবিকা<br>6. BRAC SK /ব্র্যাক স্বাস্থ্যকর্মী<br>7. PushtiKormi/পুষ্টিকর্মী<br>8. Others NGO Worker/অন্যান্য এনজিও কর্মী<br>9. TTBA/প্রশিক্ষন প্রাপ্ত দাত্রী<br>10. TBA/ধাত্রী<br>11. Village Doctor /গ্রাম্য ডাক্তার<br>12. Homeopath Doctor/হেমিওপ্যাথিক ডাক্তার<br>13. Kabiraj/herbal healer /কবিরাজ<br>14. Spiritual healer/সর্পিল বৈদ্য<br>15. Husband/স্বামী<br>16. Mother/Mother-in-Law /মা /শাশুড়ী<br>17. Any other family member/পরিবারের অন্য কোন সদস্য	___ ___	

		18. Relative/Friends/ আত্মীয়/বন্ধু<br>19. Private clinic staff/বেসরকারী ক্লিনিকের স্টাফ<br>20. Community clinic staff/কমিউনিটি ক্লিনিকের স্টাফ<br>21. EPI center staff/টীকা কেন্দ্রের স্টাফ<br>22. Myself/নিজেই<br>23. Nobody/Never need advice /কেউ না /কখনোই না<br>24. Others (specify)/ অন্যান্য(নির্দিষ্ট করুন)		
507	Did anyone put anything inside the baby's mouth IMMEDIATELY after the birth? (জন্মের সাথে সাথেই শিশুর মুখে অন্য কিছু দেওয়া হয়েছিল কি?)	1. Yes/হ্যাঁ<br>2. No/না	___	If 2 (No), go to 510 যদি না(২)হয়, তবে ৫১০ যেতে হবে
508	What was put in the baby's mouth IMMEDIATELY after birth?(জন্মের পর পর আপনার শিশুর মুখে কি দেওয়া হয়েছিল?)<br><br>[Multiple responses allowed; do NOT read list to participant]<br>(একাধিক উত্তর প্রযোজ্য: উত্তর দাতার কাছে উত্তর বলবেন না)	1. Honey/মধু<br>2. Mustard oil /সরিষার তেল<br>3. Plain water /পানি<br>4. Sugar/glucose water/চিনি/গ্লুকোজ পানি<br>5. Tea/coffee/ চা/ কফি<br>6. Cow/goat milk /গরু/ছাগলের দুধ<br>7. Breast milk /বুকের দুধ<br>8. Other (specify)/অন্যান্য(নিদির্ষ্ট করুন)<br>99 Do not remember/মনে নেই	___<br>___<br>___<br>[Specify: ___________	
509	Who put this in the baby's mouth immediately after birth?(জন্মের পর পরই বুকের দুধ খাওয়ানো সময় কে আপনাকে সাহায্য করেছিল?)	1. Hospital/Upazila Health Center, Clinic /হাসপাতাল/উপজেলা স্বাস্থ্য কেন্দ্র/ক্লিনিক<br>2. Doctor /ডাক্তার<br>3. Midwife/nurse/ধাত্রী/ নার্স<br>4. Govt. Heath Worker (FWA/HA) /সরকারী স্বাস্থ্যকর্মী<br>5. BRAC SS /ব্র্যাক স্বাস্থ্য সেবিকা	___ ___	

		6. BRAC SK/ ব্র্যাক স্বাস্থ্যকর্মী<br>7. PushtiKormi/পুষ্টিকর্মী<br>8. Others NGO Worker /অন্যান্য এনজিও কর্মী<br>9. TTBA /প্রশিক্ষন প্রাপ্তধাত্রী<br>10. TBA /ধাত্রী<br>11. Village Doctor /গ্রাম্য ডাক্তার<br>12. Homeopath Doctor /হোমিওপ্যাথিক ডাক্তার<br>13. Kabiraj/herbal healer/কবিরাজ<br>14. Spiritual healer/সপির্ল বৈদ্য<br>15. Husband/স্বামী<br>16. Mother/Mother-in-Law /মা/শাশুড়ী<br>17. Any other family member /পবিারের অন্য কোন সদস্য<br>18. Relative/Friends /আত্মীয়/বন্ধু<br>19. Private clinic staff/বেসরকারী ক্লিনিকের স্টাফ<br>20. Community clinic staff/কমিউনিটি ক্লিনিকের স্টাফ<br>21. EPI center staff/টীকা কেন্দ্রের স্টাফ<br>22. Myself/নিজেই<br>23. Nobody/Never need advice/কেউ না/কখনো কেউ উপদেশ দেয়নি<br>24. Others (specify)/অন্যান্য (নির্দিষ্ট করুন)		
510	During the first 3 days after the baby was born, what was given to the baby to drink or swallow by you or anyone else? [Multiple responses allowed]<br>(জন্মের তিন দিনের মধ্যে শিশুর মুখে কি দেওয়া হয়েছিল?)	1. Honey/মধু<br>2. Mustard oil/সরিষার তেল<br>3. Plain water/ পানি<br>4. Sugar/glucose water /চিনি/গ্লুকোজ পানি<br>5. Tea/coffee/চা/কফি<br>6. Cow/goat milk (গরু/ছাগলের দুধ)<br>7. Breast milk /বুকের দুধ	___<br>___<br>___<br>[Specify: ________	

	(একাধিক উত্তর প্রযোজ্য: উত্তর দাতার কাছে উত্তর বলবেন না)	8. Other (specify)/অন্যান্য(নির্দিষ্ট করুন)<br>99 Do not remember /মনে নেই		
511	Did you give the baby colostrum? /আপনি শিশুকে শাল দুধ দিয়েছিলেন কি?	১. Yes/হ্যাঁ<br>2. No/না	___	
512	From birth until now, has this baby ever been given expressed breast milk (in a cup or bowl)?/জন্মের পর থেকে আজ পযর্ন্ত শিশুকে কখনো বুকের দুধ গালিয়ে খাওয়ানো হয়েছে কি?(কাপ/বাটি)	1. Yes/হ্যাঁ<br>2. No/না	___	
513	Is the baby still breast feeding (বাচ্চা কি এখনো বুকের দুধ খাচ্ছে?)	1. Yes/হ্যাঁ<br>2. No/না	___	If 1 (Yes), go to 516/যদি হাঁ হয় তবে ৫১৬ যেতে হবে.
514	If no, at what age did you stop breastfeeding? (যদি না হয় তাহলে কবে/কোন বয়সে বুকের দুধ খাওয়ানো বন্ধ করেছিলেন?)	[response in months; if stopped before child one month of age, enter 0]	___	Response in days (দিনে উল্লেখ করুন)
515	Why did you stop breastfeeding? [Multiple responses allowed] (কেন বুকের দুধ খাওয়া বন্ধ করেছিলেন?)	1. Problems with breast (pain)বুকের সমস্যা(ব্যথা)<br>2. Child not suck well /শিশু ঠিকমত বুকের দুধ টানতে পারে না<br>3. Not enough time to feed child/শিশুকে খাওয়ানোর জন্য পর্যাপ্ত সময়ের অভাব<br>4. Mother got pregnant /মা পুনরায় গর্ভবতি হওয়া<br>5. Cracked nipples /বোটায় সমস্যা/ঘা যুক্ত বোটা<br>6. Felt not enough breastmilk /বুকে পর্যাপ্ত দুধ না থাকা<br>7. Other (specify)/অন্যান্য (নির্দিষ্ট করুন)	___<br>___<br>___<br>Specify: ________<br>___	
516	How many times did you breastfeed your baby yesterday, during the day and night? (গতকাল সারাদিনে ও রাতে আপনি কতবার বাচ্চাকে বুকের দুধ খাইয়েছিলেন?)		___ ___	

517	Can you tell me what the baby was fed yesterday? /গতকাল সারাদিনে বাচ্চাকে কি খাইয়েছিলেন?	1. Breastmilk only/শুধুমাত্র বুকের দুধ<br>2. Breastmilk plus water/বুকের দুধ আর পানি<br>3. Breastmilk plus any other liquids or food/বুকের দুধ আর অন্য কোন তরল খাবার<br>4. Other (specify):_____/অন্যান্য (নির্দিষ্ট করুন)	___	
518	What was the baby fed yesterday besides breastmilk? /গতকাল শিশুকে বুকের দুধের পাশাপাশি আর কি খাওয়ানো হয়েছিল?<br>a. Breast milk/বুকের দুধ<br>b. Water/পানি<br>c. Baby formula (prepared food for child)/প্রক্রিয়াজাত শিশুর খাদ্য<br>d. Any other kind of milk (powder, cow/goat milk etc.)/অন্য কোন ধরনের দুধ(পাউডার/গরু/ছাগলের দুধ)<br>e. Fruit juice (made at home) ফলের জুস(বাসায় তৈরী)<br>f. Fruit j uice (purchased, packaged) (ফলের জুস প্যাকেটজাত)<br>g. Water-based /liquids, teas, sugar water, coffee/পানিযুক্ত তরল খাবার(চা/চিনির পানি/কফি)<br>h. Other (specify): /অন্যান্য (নির্দিষ্ট করুন)	1=Yes (হ্যাঁ)<br>2=No (না)	a. ___<br>b. ___<br>c. ___<br>d. ___<br>e. ___<br>f. ___<br>g. ___<br>h. ___<br>Specify:<br>___________	
519	Do you feel you are having any problems breastfeeding? /আপনি কি শিশুকে বুকের দুধ খাওয়াতে কোন সমস্যা অনুভব করেন?	1. Yes/হ্যাঁ<br>2. No/না	___	If 2 (No), go to 600 if recently-delivered from control area. Go to 700 if >60 days postpartum or living in intervention

				area./যদি না হয় তবে ৬০০ যেতে হবে
520	If yes, what problems do you currently face? [Multiple answers allowed]/যদি হ্যাঁ হয় আপনি বর্তমানে বুকের দুধ খাওয়াতে কি সমস্যা অনুভব করছেন?	1. Problems with breast (pain) /বুকের সমস্যা(ব্যথা)<br>2. Baby not sucking/latching well /শিশু ঠিকমত বুকের দুধ টানতে পারে না<br>3. Not able to breastfeed well /ঠিক মত খাওয়াতে না পারা<br>4. Not enough time to feed baby /শিশুকে খাওয়ানোর জন্য পর্যাপ্ত সময়ের অভাব<br>5. Cracked nipples /ঘাঁ যুক্ত বোটা<br>6. Feel not enough breastmilk/বুকে পর্যাপ্ত দুধ না থাকা<br>7. Poor appetite /শিশু খেতে অনিহা<br>8. Baby sick/শিশুর অসুখ<br>9. Other (specify)/অন্যান্য (নির্দিষ্ট করুন)	___<br>___<br>___<br>___<br>[Specify: /নির্দিষ্ট করুন	
521	Did you seek help from anyone for this/these problems?/আপনি কি উপরে উল্লেখিত সমস্যা সমূহের জন্য কারো সাহায্য নিয়েছিলেন?	1. Yes/হ্যাঁ<br>2. No/না	___	If no Go to 600 for control area.<br>যদি না হয় তবে ৬০০ যেতে হবে (কন্ট্রোল এলাকার জন্য)<br>Go to 700 living in both areas.<br>(উভয় এলাকার জন্য প্রশ্ন নাম্বার ৭০০ তে যান)
Recently Delivered Women (Within 0-60 days of delivery) Control Areas only<br>সদ্য প্রসুতি মহিলা (প্রসবের ০ থেকে ৬০ দিনের মধ্যে) কন্টোল এরিয়ার জন্য				
600	Number IFA tablets reported consumedin the last month/শেষ মাসে কতটা আয়রণ ট্যাবলেট খেয়েছেন?	Enter 99 for don't know	___ ___	
601	Number calcium tablets reported consumed in the	Enter 99 for don't know	___ ___	

	last month (শেষ মাসে কতটা ক্যালসিয়াম ট্যাবলেট খেয়েছেন?)			
602	Fish/ Meat/Liver taken within last 24 hours (গত ২৪ ঘন্টায় মাছ/মাংস/কলিজি খেয়েছেন কি?)	1. Yes/হ্যাঁ<br>2. No/না	___	If no, skip to 604./না হলে ৬০৪ যেতে হবে
603	Amount (bowl) পরিমান	Enter stated number of bowl(s), or 99 for missing data/বোলে পরিমান লিখতে হবে	______ ______	
604	Egg taken within 24 hours (গত ২৪ ঘন্টায় ডিম খেয়েছেন কি)	1. Yes/হ্যাঁ<br>2. No/না	___	If no, skip to 606./না হলে ৬০৬ যেতে হবে
605	Amount (bowl)/পরিমান	Enter stated number of bowl(s), or 99 for missing data/বোলে পরিমান লিখতে হবে/	____ ____	
606	Milk /Milk Products taken within last 24 hours/গত ২৪ ঘন্টায় দুধ/ দুধের তৈরী খাবার খেয়েছেন কি?	1. Yes/হ্যাঁ<br>2. No/না	___	If no, skip to 608./না হলে ৬০৮ যেতে হবে
607	Amount (bowl)/পরিমান	Enter stated number of bowl(s), or 99 for missing data/পরিমান বোলে লিখতে হবে	____ ____	
608	Orange/yellow vegetable/fruit taken within last 24 hours/গত ২৪ ঘন্টায় হলুদ/কমলা ফল/ শব্জি খেয়েছেন কি?	1. Yes/হ্যাঁ<br>2. No/না	___	If no, skip to 610./না হলে ৬১০ যেতে হবে
609	Amount (bowl)/পরিমান	Enter stated number of bowl(s), or 99 for missing data/পরিমান বোলে লিখতে হবে	____ ____	
610	Rice/ Bread taken within last 24 hours/গত ২৪ ঘন্টায় ভাত/রুটি খেয়েছেন কি?	1. Yes/হ্যাঁ<br>2. No/না	___	If no, skip to 612./না হলে ৬১২ যেতে হবে
611	Amount (bowl)/পরিমান	Enter stated number of bowl(s), or 99 for missing data/পরিমান বোলে লিখতে হবে	____ ____	
612	Dark Green Vegetables taken within last 24 hours/গত ২৪ ঘন্টায় গাঢ় সবুজ শাক খেয়েছেন কি?	1. Yes/হ্যাঁ<br>2. No/না	___	If no, skip to 614./না হলে ৬১৪ যেতে হবে

613	Amount (bowl)/পরিমান	Enter stated number of bowl(s), or 99 for missing data/ পরিমান বোলে লিখতে হবে	____ ____	If no, skip to 616./না হলে৬১৬ যেতে হবে
614	Thick Lentils taken within last 24 hours/গত ২৪ ঘন্টায় ঘন ডাল খেয়েছেন কিনা	1. Yes/হ্যাঁ<br>2. No/না	___	
615	Amount (bowl)/পরিমান	Enter stated number of bowl(s), or 99 for missing data/পরিমান বোলে লিখতে হবে	____ ____	
Current Functional Ability (বর্তমানে কার্যক্ষমতা)				
Please state whether you can currently do the following activities: (আপনি কি বর্তমানে নিম্নোক্ত কাজ সমূহ করতে পারেন?)				
700	Take care of the newborn baby? (আপনি কি সদ্য জন্মগ্রহন কৃত বাচ্চার যত্ন নিতে পারেন?)	1. Yes/হ্যাঁ<br>2. No/না	___	
701	Feed the baby(বাচ্চাকে খাওয়াতে পারেন?)	1. Yes/হ্যাঁ<br>2. No/না	___	
702	Wash the baby (শিশুকে পরিষ্কার করতে পারেন?)	1. Yes/হ্যাঁ<br>2. No/না	___	
703	Wash the babies clothes?/শিশুর কাপড় পরিষ্কার করতে পারেন?	1. Ye/হ্যাঁ<br>2. No/না	___	
704	Prepare meals?/খাবার তৈরী করতে পারেন?	1. Yes/হ্যাঁ<br>2. No/না	___	
705	Clean the house? (বাড়ী পরিষ্কার করতে পারেন?)	1. Yes/হ্যাঁ<br>2. No/না	___	
706	Get water? (পানি আনতে পারেন?)	1. Yes/হ্যাঁ<br>2. No/না	___	
707	Go to the facility? (স্বাস্থ্য কেন্দ্রে যেতে পারেন?)	1. Yes/হ্যাঁ<br>2. No/না	___	
708	Take care of yourself (নিজের যত্ন নিতে পারেন?)	1. Yes/হ্যাঁ<br>2. No/না	___	
709	Wash or bathe yourself (নিজে গোসল করতে পারেন?)	1. Yes/হাঁ<br>2. No/না	___	
710	Dress yourself (নিজে নিজে কাপড় পড়তে পারেন?)	1. Yes/হ্যাঁ<br>2. No/না	___	
711	Wash your own clothes(নিজের কাপড় পরিষ্কার করতে পারেন?)	1. Yes/হ্যাঁ<br>2. No/না	___	
If any of the above answers are 1 (Yes), go to the corresponding question/activity below:				

যদি উপররেে (৭০০-৭১১) এর উত্তর ১ হয় তাহলে উক্ত প্রশ্নের উত্তরের সাথে সামাজস্য পূর্ণ নিচের উত্তর দিন					
	Answer code: 1. Could do without difficulty (কোন সমস্যা ছাড়া কাজ করতে সক্ষম)<br>2. Could do with difficulty কিঞ্চিত সমস্যা হলেও কাজ করতে সক্ষম<br>3. Could not do at all /একেবারেই কাজ করতে অক্ষম<br>99. Not applicable/প্রযোজ্য নয়	A. Within 4-7 days of delivery প্রসবের ৪-৭ দিনের মধ্যে	B.Within 8-14 days of delivery প্রসবের ৮-১৪ দিনের মধ্যে	C.Within 2-4 weeks of delivery প্রসবের ২ থেকে ৪ সপ্তাহের মধ্যে	D.Within 4-6 weeks of delivery প্রসবের ৪-৬ সপ্তাহের মধ্যে
712	Were you able to take care of the newborn baby? আপনি কি সদ্য জন্মগ্রহন কৃত শিশুর যত্ন নিতে পারেন?	ক. _____	খ. _____	গ. _____	ঘ. _____
713	Were you able to feed the baby/আপনি কি শিশু কে খাওয়াতে পারেন?	ক. _____	খ. _____	গ. _____	ঘ. _____
714	Were you able to wash the baby/আপনি কি শিশুকে গোসল করাতে সক্ষম?	ক. _____	খ. _____	গ. _____	ঘ. _____
715	Were you able to wash the babies clothes?/আপনি কি শিশুর কাপড় পরিষ্কার করতে সক্ষম?	ক. _____	খ. _____	গ. _____	ঘ. _____
716	Were you able to prepare meals?/আপনি কি শিশুর খাবার তৈরী করতে সক্ষম?	ক. _____	খ. _____	গ. _____	ঘ. _____
717	Were you able to clean the house?/আপনি কি বাড়ী পরিষ্কার করতে সক্ষম?	ক. _____	খ. _____	গ. _____	ঘ. _____
718	Were you able to get water?/আপনি কি পানি আনতে সক্ষম?	ক. _____	খ. _____	গ. _____	ঘ. _____
719	Were you able to go to the facility?আপনি স্বাস্থ্য কেন্দ্রে যেতে সক্ষম?	ক. _____	খ. _____	গ. _____	ঘ. _____
720	Were you able to care of herself ./আপনি কি নিজের যত্ন নিজেই নিতে সক্ষম?	ক. _____	খ. _____	গ. _____	ঘ. _____
721	Were you able to wash or bathe herself/ আপনি কি নিজে গোসল করতে সক্ষম?	ক. _____	খ. _____	গ. _____	ঘ. _____
722	Were you able to get dressed./আপনি নিজে নিজে কাপড় পড়তে সক্ষম?	ক. _____	খ. _____	গ. _____	ঘ. _____

723	Were you able to wash clothesআপনি কি নিজের কাপড় পরিষ্কার করতে সক্ষম?	ক. _____	খ. _____	গ. _____	ঘ. _____
Community-based Activity Exposure					
800	Have you heard of any forums for husband's regarding maternal nutrition and health in your community?/আপনি কি আপনার এলাকায় স্বামীদের নিয়ে আয়োজিত গর্ভ কালীন পুষ্টি ও স্বাস্থ্য বিষয়ক সভা সম্পর্কে শুনেছেন?	1. Yes/হ্যাঁ<br>2. No/না		___	
801	Has your husband attended any of these forums?/আপনার স্বামী ডশ এমন কোন সভায় অংশগ্রহন করেছিলেন	1. Yes/হ্যাঁ<br>2. No/না		___	If 2 (No), thank the participant and end interview./ যদি না হয় তবে অংশগ্রহনকারীকে ধন্যবাদ দিয়ে ইন্টারভিউ শেষ করুন
802	Did your husband take any action after coming back from the forum?/আপনার স্বামী কি সভা থেকে আসার পর কোন ধরনের উদ্যোগ গ্রহন করেছিলেন?	1. Yes/হ্যাঁ<br>2. No/না		___	
803	If yes, what actions did he take?<br>যদি হ্যাঁ হয় তবে তিনি কি/কোন ধরনের উদ্যোগ গ্রহন করেছিলেন?<br>[More than one response permitted, DO NOT read list to participant.]<br>(একাধিক উত্তর প্রযোজ্য: উত্তর দাতার কাছে উত্তর বলবেন না)	1. Procure/ensure adequate food supply for you /পর্যাপ্ত খাবারের সরবরাহ নিশ্চিত করা/ব্যবস্থা করা<br>2. Reminds and encourages you to consume the recommended quantity of diversified foods daily/প্রত্যেক দিন বিভিন্ন ধরনের খাবার খাওয়াতে উৎসাহিত করা<br>3. Helps you to ensure that there are enough tablets of IFA and Calcium at home/বাসায় আয়রন ও		1. ___<br>2. ___<br>3. ___	Thank the participant and end interview./<br>অংশগ্রহনকারীকে ধন্যবাদ দিয়ে ইন্টারভিউ শেষ কর[illegible]ন ।

		ক্যালসিয়াম ট্যাবলেটের পর্যাপ্ততা নিশ্চিত করা<br>4. Remind you to take one tablet of IFA daily daily/প্রত্যেক দিন একটা করে আয়রন ট্যাবলেট খেতে উৎসাহিত করা<br>5. Remind you to take one tablet of Calcium daily/প্রত্যেক দিন একটা করে ক্যালসিয়াম ট্যাবলেট খেতে উৎসাহিত করা<br>6. Remind /helps you to take rest for 2 hours/day /প্রত্যেক দিন ২ ঘন্টা কওে বিশ্রাম নিতে সাহায্য/মনে করিয়ে দেওয়া<br>7. Did not let you lift heavy work load during pregnancy/গর্ভকালীন সময়ে ভারী কাজ করতে নিরুৎসাহিত করা/করতে না দেওয়া<br>8. Calls the health worker on mobile if you had any difficulties related to pregnancy /গর্ভকালীন সময়ে কোন জটিলতা হলে স্বাস্থ্য কর্মীকে ফোন করা<br>9. Did not do anything differently /আলাদা করে কিছু করেনি।	4. ___<br>5. ___<br>6. ___<br>7. ___<br>8. ___<br>9. ______	
