# Supplementary material for: Maternal nutrition intervention and maternal complications in 4 districts of Bangladesh: A nested cross-sectional study
Source: PLoS Med. 2019 Oct 4;16(10):e1002927. doi: 10.1371/journal.pmed.1002927 (PMC6777761; doi:10.1371/journal.pmed.1002927)
Supplement: S1 Table — (DOCX) [file pmed.1002927.s008.docx]

| **S1 Table. Hierarchical logistic regression models assessing association of reported antepartum complications between women exposed to a maternal nutrition intervention and those in control areas in four districts of Bangladesh.** | | |
| --- | --- | --- |
|  | ***(Crude Model, n=1100)*** | ***(Adjusted Model, n=1080)*** |
|  | ***(OR, 95% CI)*** | ***AOR, (95% CI)*** |
| Intervention exposure | 0.534 | 0.532 |
|  | [0.241,1.184] | [0.270,1.047] |
| Age |  | 1.003 |
|  |  | [0.976,1.032] |
| Reported malnutrition |  | 5.311^*^ |
|  |  | [1.218,23.16] |
| Prior pregnancy complication |  | 1.314 |
|  |  | [0.396,4.360] |
| Peri-partum complications in index pregnancy |  | 5.142^***^ |
|  |  | [3.143,8.410] |
| Owns house |  | 1.429 |
|  |  | [0.723,2.826] |
| Owns land |  | 0.703^*^ |
|  |  | [0.505,0.979] |
| Electricity |  | 1.248 |
|  |  | [0.858,1.815] |
| Number of TVs |  | 1.037 |
|  |  | [0.708,1.519] |
| Number of motorcycles |  | 0.945 |
|  |  | [0.519,1.722] |
| Number of phones |  | 1.043 |
|  |  | [0.874,1.245] |
| Income Quintiles |  |  |
| 1 |  | Ref |
| 2 |  | 0.498 |
|  |  | [0.219,1.134] |
| 3 |  | 1.036 |
|  |  | [0.502,2.140] |
| 4 |  | 0.941 |
|  |  | [0.471,1.882] |
| 5 |  | 1.523 |
|  |  | [0.678,3.424] |
| District indicators | No | Yes |
|  |  |  |
| Source of drinking water indicators | No | Yes |
| Exponentiated coefficients; 95% confidence intervals in brackets; ^*^ *p* < 0.05, ^**^ *p* < 0.01, ^***^ *p* < 0.001 | | |
| *AIC* | 723.2 | 667.9 |
| *BIC* | 738.2 | 777.6 |
