# Supplementary material for: Maternal nutrition intervention and maternal complications in 4 districts of Bangladesh: A nested cross-sectional study
Source: PLoS Med. 2019 Oct 4;16(10):e1002927. doi: 10.1371/journal.pmed.1002927 (PMC6777761; doi:10.1371/journal.pmed.1002927)
Supplement: S2 Table — (DOCX) [file pmed.1002927.s009.docx]

| **S2 Table. Hierarchical logistic regression models assessing association of reported overall intrapartum complications between women exposed to a maternal nutrition intervention and those in control areas in four districts of Bangladesh.** | | |
| --- | --- | --- |
|  | ***(Crude Model 1, n=1099)*** | ***(Adjusted Model 2, n=1094)*** |
|  | ***OR (95% CI)*** | ***AOR (95% CI)*** |
| Treatment | 0.728 | 0.897 |
|  | [0.469, 1.129] | [0.607, 1.327] |
| Antepartum complications |  | 2.493^***^ |
|  |  | [1.816, 3.423] |
| Age |  | 0.981 |
|  |  | [0.958, 1.006] |
| Malnutrition |  | 1.751 |
|  |  | [0.851, 3.603] |
| Prior pregnancy complication |  | 0.739 |
|  |  | [0.255, 2.139] |
| Owns house |  | 0.610 |
|  |  | [0.354, 1.053] |
| Owns land |  | 1.015 |
|  |  | [0.764, 1.349] |
| Electricity |  | 1.054 |
|  |  | [0.757, 1.469] |
| Number of TVs |  | 1.146 |
|  |  | [0.830, 1.580] |
| Number of motorcycles |  | 0.825 |
|  |  | [0.486, 1.403] |
| Number of phones |  | 1.090 |
|  |  | [0.936, 1.268] |
| Income Quintiles |  |  |
| 1 |  | Ref |
| 2 |  | 1.050 |
|  |  | [0.658,1.674] |
| 3 |  | 0.781 |
|  |  | [0.501,1.217] |
| 4 |  | 0.923 |
|  |  | [0.603,1.411] |
| 5 |  | 1.186 |
|  |  | [0.731,1.927] |
| District indicators | No | Yes |
| Source of drinking water indicators | No | Yes |
| Exponentiated coefficients; 95% confidence intervals in brackets; ^*^ *p* < 0.05, ^**^ *p* < 0.01, ^***^ *p* < 0.001 | | |
| *AIC* | 1491.6 | 1467.2 |
| *BIC* | 1506.6 | 1587.1 |
