# Supplementary material for: Maternal nutrition intervention and maternal complications in 4 districts of Bangladesh: A nested cross-sectional study
Source: PLoS Med. 2019 Oct 4;16(10):e1002927. doi: 10.1371/journal.pmed.1002927 (PMC6777761; doi:10.1371/journal.pmed.1002927)
Supplement: S3 Table — (DOCX) [file pmed.1002927.s010.docx]

| **S3 Table. Hierarchical logistic regression model for differences in reported retained placenta between women exposed to a maternal nutrition intervention and those in control areas in four districts of Bangladesh.** | | |
| --- | --- | --- |
|  | ***Crude Model (n=1099)*** | ***Adjusted Model (n=1068)*** |
|  | ***OR (95% CI)*** | ***AOR (95% CI)*** |
| Treatment | 0.391^**^ | 0.353^**^ |
|  | [0.197,0.775] | [0.186,0.671] |
| Age |  | 1.053 |
|  |  | [0.999,1.110] |
| Malnutrition |  | 1.867 |
|  |  | [0.516,6.754] |
| Hospital Delivery |  | 0.195^***^ |
|  |  | [0.0738,0.514] |
| Owns house |  | 0.351^*^ |
|  |  | [0.130,0.945] |
| Owns land |  | 0.620 |
|  |  | [0.320,1.201] |
| Electricity |  | 1.425 |
|  |  | [0.711,2.856] |
| Number of TVs |  | 1.202 |
|  |  | [0.567,2.546] |
| Number of motorcycles |  | 0.499 |
|  |  | [0.0610,4.077] |
| Number of phones |  | 0.865 |
|  |  | [0.566,1.321] |
| Income quintile indicators | No | Yes |
| District indicators | No | Yes |
| Source of drinking water indicators | No | Yes |
| Exponentiated coefficients; 95% confidence intervals in brackets; ^*^ *p* < 0.05, ^**^ *p* < 0.01, ^***^ *p* < 0.001 | | |
| *AIC* | 435.2 | 419.1 |
| *BIC* | 450.2 | 518.6 |
