# Supplementary material for: Maternal nutrition intervention and maternal complications in 4 districts of Bangladesh: A nested cross-sectional study
Source: PLoS Med. 2019 Oct 4;16(10):e1002927. doi: 10.1371/journal.pmed.1002927 (PMC6777761; doi:10.1371/journal.pmed.1002927)
Supplement: S4 Table — (DOCX) [file pmed.1002927.s011.docx]

| **S4 Table. Hierarchical logistic regression model for differences in reported intrapartum fever or infection between women exposed to a maternal nutrition intervention and those in control areas in four districts of Bangladesh.** | | |
| --- | --- | --- |
|  | ***Crude Model (n=1099)*** | ***Adjusted Model (n=1080)*** |
|  | ***OR (95% CI)*** | ***AOR (95% CI)*** |
| Treatment | 0.274^*^ | 0.271^**^ |
|  | [0.0949,0.789] | [0.113,0.653] |
| Age |  | 0.931 |
|  |  | [0.863,1.004] |
| Malnutrition |  | 0.704 |
|  |  | [0.0771,6.428] |
| Hospital Delivery |  | 1.973 |
|  |  | [0.897,4.340] |
| Owns house |  | 1.505 |
|  |  | [0.380,5.968] |
| Owns land |  | 0.329^*^ |
|  |  | [0.137,0.790] |
| Electricity |  | 0.970 |
|  |  | [0.366,2.566] |
| Number of TVs |  | 2.587^*^ |
|  |  | [1.011,6.617] |
| Number of motorcycles |  | 0.652 |
|  |  | [0.121,3.504] |
| Number of phones |  | 1.074 |
|  |  | [0.686,1.682] |
| Income quintile indicators | No | Yes |
| District indicators | No | Yes |
| Source of drinking water indicators | No | Yes |
| Exponentiated coefficients; 95% confidence intervals in brackets; ^*^ *p* < 0.05, ^**^ *p* < 0.01, ^***^ *p* < 0.001 | | |
| *AIC* | 300.3 | 307.6 |
| *BIC* | 315.3 | 412.3 |
