# Supplementary material for: Maternal nutrition intervention and maternal complications in 4 districts of Bangladesh: A nested cross-sectional study
Source: PLoS Med. 2019 Oct 4;16(10):e1002927. doi: 10.1371/journal.pmed.1002927 (PMC6777761; doi:10.1371/journal.pmed.1002927)
Supplement: S5 Table — (DOCX) [file pmed.1002927.s012.docx]

| **S5 Table. Hierarchical logistic regression models assessing association of overall postpartum complications between women exposed to a maternal nutrition intervention and those in control areas in four districts of Bangladesh.** | | |
| --- | --- | --- |
|  | ***Crude Model, n=1100*** | ***Adjusted Model, n=1091*** |
|  | ***OR (95% CI)*** | ***AOR (95% CI)*** |
| Treatment | 0.502^*^ | 0.511^**^ |
|  | [0.287, 0.881] | [0.318, 0.821] |
| Peripartum complications |  | 2.077^***^ |
|  |  | [1.572, 2.744] |
| Antepartum complications |  | 3.191^***^ |
|  |  | [2.219, 4.588] |
| Age |  | 1.018 |
|  |  | [0.992, 1.045] |
| Malnutrition |  | 3.682^**^ |
|  |  | [1.618, 8.379] |
| Prior pregnancy complication |  | 0.766 |
|  |  | [0.254, 2.307] |
| Owns house |  | 0.803 |
|  |  | [0.445, 1.450] |
| Owns land |  | 0.892 |
|  |  | [0.657, 1.212] |
| Electricity |  | 0.973 |
|  |  | [0.680, 1.394] |
| Number of TVs |  | 1.121 |
|  |  | [0.793, 1.586] |
| Number of motorcycles |  | 0.593 |
|  |  | [0.323, 1.087] |
| Number of phones |  | 0.907 |
|  |  | [0.770, 1.069] |
| Income |  |  |
| 1 |  | Ref |
| 2 |  | 1.140 |
|  |  | [0.686,1.894] |
| 3 |  | 0.879 |
|  |  | [0.543,1.422] |
| 4 |  | 0.907 |
|  |  | [0.570,1.444] |
| 5 |  | 1.068 |
|  |  | [0.630,1.811] |
| District indicators | No | Yes |
| Source of drinking water indicators | No | Yes |
| Exponentiated coefficients; 95% confidence intervals in brackets; ^*^ *p* < 0.05, ^**^ *p* < 0.01, ^***^ *p* < 0.001 | | |
| *AIC* | 1418.8 | 1321.2 |
| *BIC* | 1433.8 | 1441.1 |
