# Supplementary material for: Maternal nutrition intervention and maternal complications in 4 districts of Bangladesh: A nested cross-sectional study
Source: PLoS Med. 2019 Oct 4;16(10):e1002927. doi: 10.1371/journal.pmed.1002927 (PMC6777761; doi:10.1371/journal.pmed.1002927)
Supplement: S6 Table — (DOCX) [file pmed.1002927.s013.docx]

| **S6 Table. Hierarchical logistic regression models assessing association of reported postpartum hemorrhage between women exposed to a maternal nutrition intervention and those in control areas in four districts of Bangladesh.** | | |
| --- | --- | --- |
|  | ***Crude Model (n=1100)*** | ***Adjusted Model (n=1080)*** |
|  | ***(OR, 95% CI)*** | ***AOR, (95% CI)*** |
| Treatment | 0.412 | 0.374^*^ |
|  | [0.136,1.247] | [0.152,0.923] |
| Intrapartum complications |  | 5.142^***^ |
|  |  | [3.143,8.410] |
| Age |  | 0.981 |
|  |  | [0.942,1.023] |
| Malnutrition |  | 1.592 |
|  |  | [0.551,4.596] |
| Hospital Delivery |  | 0.548^*^ |
|  |  | [0.327,0.916] |
| Owns house |  | 1.070 |
|  |  | [0.460,2.489] |
| Owns land |  | 0.572^*^ |
|  |  | [0.346,0.944] |
| Electricity |  | 0.772 |
|  |  | [0.447,1.331] |
| Number of TVs |  | 0.606 |
|  |  | [0.340,1.081] |
| Number of motorcycles |  | 0.449 |
|  |  | [0.149,1.359] |
| Number of phones |  | 1.105 |
|  |  | [0.867,1.408] |
| Income quintile indicators | No | Yes |
| District indicators | No | Yes |
| Source of drinking water indicators | No | Yes |
| Exponentiated coefficients; 95% confidence intervals in brackets; ^*^ *p* < 0.05, ^**^ *p* < 0.01, ^***^ *p* < 0.001 | | |
| *AIC* | 723.2 | 667.9 |
| *BIC* | 738.2 | 777.6 |
